# Novel Tetracyclic Azaphenothiazines with the Quinoline Ring as New Anticancer and Antibacterial Derivatives of Chlorpromazine

**DOI:** 10.3390/ijms25084148

**Published:** 2024-04-09

**Authors:** Małgorzata Jeleń, Dagmara Otto-Ślusarczyk, Beata Morak-Młodawska, Marta Struga

**Affiliations:** 1 Department of Organic Chemistry, The Medical University of Silesia, Jagiellońska 4, 41-200 Sosnowiec, Poland; bmlodawska@sum.edu.pl; 2 Chair and Department of Biochemistry, Medical University of Warsaw, 02-097 Warsaw, Poland; dagmara.otto@wum.edu.pl (D.O.-Ś.); marta.struga@wum.edu.pl (M.S.)

**Keywords:** azaphenothiazine, chlorpromazine, quinobenzothiazine, anticancer activity, antibacterial activity, apoptotic activity, cell cycle arrest

## Abstract

Phenothiazine derivatives are widely studied in various fields such as biology, chemistry, and medicine research because of their pharmaceutical effects. The first compound used successfully in the treatment of psychosis was a phenthiazine derivative, chlorpromazine. Apart from its activity in neurons, chlorpromazine has also been reported to display anticancer and antibacterial properties. In this study, we present the synthesis and research on the activity of A549, MDA, MiaPaCa, PC3, and HCT116 cancer cell lines and of *S. aureus*, *S. epidermidis*, *E. coli*, and *P. aeruginosa* bacterial strains against a series of new tetracyclic chlorpromazine analogues containing a quinoline scaffold in their structure instead of the benzene ring and various substituents at the thiazine nitrogen. The structure of these novel molecules has been determined by ^1^H NMR, ^13^C NMR, and HRMS spectral techniques. The seven most active of the twenty-four new chlorpromazine analogues tested were selected to study the mechanism of cytotoxic action. Their ability to induce apoptosis or necrosis in cancer cells was assessed by flow cytometry analysis. The results obtained confirmed the proapoptotic activity of selected compounds, especially in terms of inducing late apoptosis or necrosis in cancer cell lines A549, MiaPaCa-2, and HCT-116. Furthermore, studies on the induction of cell cycle arrest suggest that the new chlorpromazine analogues exert antiproliferative effects by inducing cell cycle arrest in the S phase and, consequently, apoptosis.

## 1. Introduction

Heterocyclic compounds containing sulfur and nitrogen atoms occupy a special place in medicinal chemistry due to their wide range of biological activities. Such heterocycles include a large group of β-lactam antibiotics and drugs containing a thiazole ring (including ritonavir, cobicistat, nizatidine, famotidine, meloxicam, mirabegron, and dasatinib) [1,2,3]. This group includes also aromatic tricyclic compounds in which two benzene rings are linked by sulfur and nitrogen atoms—phenothiazines. These compounds were initially used in the dye and pigment industry and as insecticides, and quickly became the parent molecule of a multitude of drugs that have varied uses throughout medical and veterinary practice. For example, 10*H*-dibenzo-1,4-thiazine possesses insecticidal, antifungal, antibacterial, and anthelmintic properties [4].

One of the first important uses of phenothiazines, in addition to their use in the dye industry, was for their toxic effect on mosquito larvae and as anthelmintics (effective against swine roundworm) and antimalarials; however, they were not widely used for these purposes [5].

The history of these compounds goes back to the second half of the 19th century. Chlorpromazine, which belongs to this group and was initially used as an anesthetic, revolutionized the treatment of psychiatric disorders. It was originally synthesized by scientists at Rhone-Poulenc with the hopes that it would be an effective antimalarial [6,7]. Chlorpromazine has been one of the most widely used antipsychotic medications for the treatment of schizophrenia and other psychiatric disorders. Although chlorpromazine is a first-generation antipsychotic medication, it is still widely used in psychiatry [8]. Chlorpromazine demonstrates a high affinity for dopamine (D_1_–D_4_) receptors and acts as a receptor antagonist by inhibiting adenylatecyclase activity. Chlorpromazine also inhibits other receptors, including receptors for 5-HT, H_1_ histamine, α1 and α2 adrenaline, and M_1_ and M_2_ muscarinic acetylcholine receptors. N-Methyl-D-aspartate (NMDA) receptor inhibitory effects have also been described at high concentrations of chlorpromazine [9,10,11].

Several studies have reported on the potential anticancer activity of dopamine receptor antagonists. Among the drugs belonging to the neuroleptic phenothiazine, not only chlorpromazine, but also perphenazine, prochlorperazine, fluphenazine, and thioridazine have been tested for anticancer activity [12,13,14].

From the very beginning of the use of chlorpromazine in psychiatry, its impact on the course of cancer diseases has been observed. Among other things, studies conducted in Denmark in the second half of the twentieth century suggested a reduced risk of cancer in chlorpromazine-treated psychiatric patients. Significant inhibition of tumor growth has also been reported in a patient with laryngeal squamous cell carcinoma after direct injection of chlorpromazine into the tumor. Chlorpromazine was also shown to inhibit the growth of sarcoma tumors in mice. In line with the drug repurposing strategy, chlorpromazine was tested for its potential antitumorigenic effects, among others, against colorectal, breast, brain, lung, skin, pancreatic, and oral cancers, and also against leukemia, lymphoma, sarcoma, mastocytoma, and glioblastoma [15,16,17,18,19,20,21,22,23,24,25,26,27,28,29,30,31].

The good results obtained in studies on cancer cell lines coupled with animal studies drew attention to chlorpromazine as a potential antitumor medication, leading to follow-up studies focused on chlorpromazine’s anticancer mechanisms. Published research results have found that chlorpromazine inhibits cancer growth through multiple independent pathways, through various targets, ranging from histone deacetylases to ion channels. The overlap of molecular pathways between schizophrenia and cancer has been suspected for many years [32,33,34,35,36].

Therefore, in parallel with multidirectional research on neuroleptic phenothiazines, syntheses and research on the activity of new phenothiazine derivatives are carried out. New syntheses are realized in many ways, new substituents are introduced to the thiazine nitrogen atom and/or to the carbons of benzene rings. It is also increasingly common to replace one or both benzene rings with monocyclic or bicyclic azine systems (pyridine, pyrimidine, pyridazine, pyrazine, or quinoline) [37,38,39,40,41]. Among the many activities determined for new phenothiazine analogues, such as antibacterial, antifungal, anti-tubercular, antiviral, anti-inflammatory, anti-filarial, antimalarial, anti-parasitic, and multidrug resistance reversal, the anticancer properties are of particular interest [42].

**Figure 1:**
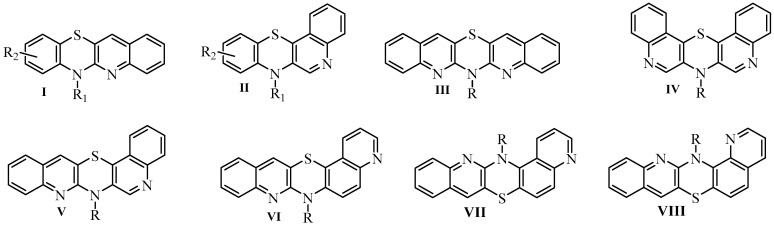
Linearly and angularly condensed quinobenzothiazines and diquinothiazines.

Phenothiazines containing one or two quinoline moieties instead of benzene rings are quinobenzothiazines and diquinothiazines. Selected compounds from substituted quinobenzothiazines **I** and **II** and diquinothiazines **III**–**VIII** (Figure 1) show significant anticancer activity against dozens of cancer cells derived from leukemia, melanoma, non-small cell lung, colon, CNS, ovary, prostate, breast, and skin cancers [37,40,41,43,44]. These compounds also show promising antioxidant effects on rat liver microsomal membranes to protect non-enzymatic lipid peroxidation, inhibitory effects on mitogen-induced proliferation of human peripheral blood mononuclear cells, production of tumor necrosis factor-alpha (TNFα) in human whole blood cultures, against butyrylcholinesterase, and free radical scavenging, antiglycation, and alpha-glucosidase and alpha-amylase inhibition [37,40,44]. They exerted a suppressive effect in in vivo models: delayed-type hypersensitivity to ovalbumin and cutaneous reaction to carrageenan, contact sensitivity to oxazolone, and experimental psoriasis in mice, and showed inhibitory effects on the expression of IFNβ and IFNβ-dependent further genes and proteins involved in the pathogenesis of autoimmune diseases [37,40]. Some of the phenothiazines modified with the quinoline ring have also shown therapeutic effects in mouse experimental colitis, and prolongation of skin graft survival in mice [45].

**Figure 2:**
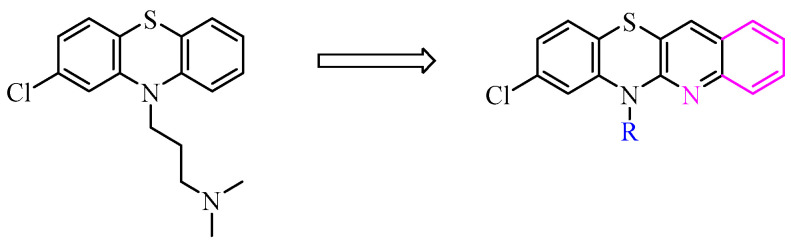
Chlorpromazine structure modification scheme.

In these studies, we developed an efficient synthesis of 6*H*-8-chloroquinobenzothiazine as a substrate to obtain a series of new chlorpromazine analogues, which have a quinoline scaffold in their structure instead of the benzene ring and various substituents at the thiazine nitrogen atom (Figure 2). The starting point for planning such a modification was the significant and promising in vitro and in vivo activities of previously obtained 9-chloroquinobenzothiazine derivatives [37,38,40] and the great importance of chlorpromazine as a leading structure in medicinal chemistry.

## 2. Results and Discussion

### 2.1. Synthesis

**Scheme 1:**
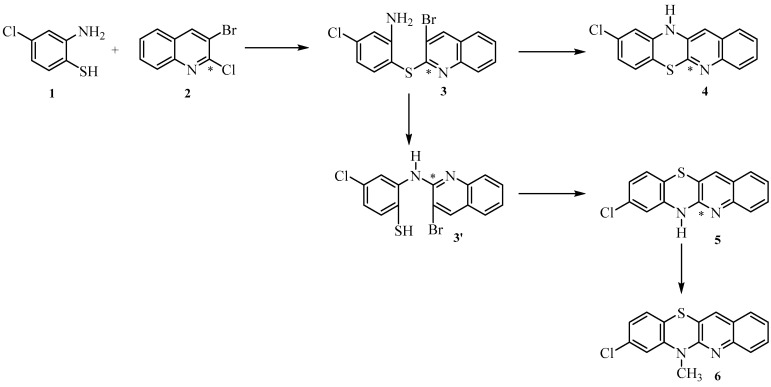
Possible directions of the reaction of 2-amino-4-chlrobenzenethiol **1** and 3-bromo-2-chloroquinoline **2**.

For the synthesis of 6*H*-8-chloroquinobenzothiazine **5**, 2-amino-4-chlorobenzenethiol **1** and 3-bromo-2-chloroquinoline **2** (Scheme 1) were used as starting materials. The reaction was carried out in boiling DMF for 1 h. Phenyl quinoline sulfide **3** is formed as an intermediate product in this type of phenothiazine ring synthesis reaction. Sulfide **3** can then undergo transformations in two directions. There is the possibility of direct cyclization (the Ullmann cyclization) toward quinobenzothiazine **4**. The second variant leads through the Smiles rearrangement reaction (the S→N type, quinolinyl part migrates from the sulfur atom to the nitrogen atom, not isolated) to an amine **3′**, which then undergoes cyclization to quinobenzothiazine **5**. The literature data show that the course of this type of reaction for the synthesis of phenothiazine systems most often depends on the conditions used [37,46,47,48,49]. Sometimes, it is impossible to state whether a reaction goes with or without the rearrangement because Ullmann’s and Smiles’s products are the same. The rearrangement proceeds under basic (most often) conditions, but also under acidic and neutral conditions. Using substituted *o*-aminobenzenethiol as a substrate, as in the reaction described, the possibility of creating two phenothiazines **4** and **5** should be considered.

**Scheme 2:**
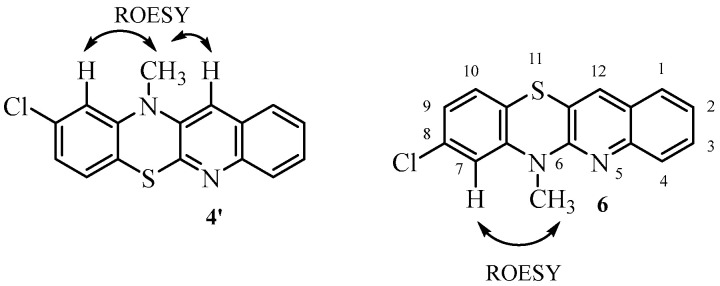
^1^H-^1^H ROESY connection in compounds **4′** and **6**.

6*H*-8-Chloroquinobenzothiazine **5** was obtained in our previous studies by reacting 2,2′-dichloro-3,3′-diquinolinyl disulfide with 2,5-dichloroaniline [50]. Comparison of the substances obtained in these reactions allowed the preliminary assumption that the reaction of 2-amino-4-chlrobenzenethiol **1** and 3-bromo-2-chloroquinoline **2** involves a Smiles rearrangement. In order to identify unequivocally the structure of quinobenzothiazine **5**, we transformed it into 6-methyl derivative **6** and carried out ^1^H NMR and two-dimensional NOESY and COSY spectra (Scheme 2, Table 1).

The ^1^H NMR spectra were very useful for identification of the product as 8-substituted quinobenzothiazine **6**. The proton signals of the quinoline part were found at low field (over 7.3 ppm) as a singlet (proton H12), doublet signal with one ortho-coupling (proton H4), triplet signal with two ortho-couplings (proton H2), doublet (proton H1), and triplet (proton H3). The proton signals of the benzene ring were found at a high field (below 7.1 ppm) and are observed as three doublets differing in shape and multiplicity depending on the proton to which they are assigned. The H10 and H9 (as double doublet signals) proton signals appear in the form of wide doublets with a coupling constant of 7, while the H7 proton signal appears in the form of a very narrow doublet with a coupling constant of 1.8.

In order to fully document the structure of derivative **6**, a ^13^C NMR spectrum and two-dimensional HSQC and HMBC spectra were also performed, which allowed for the assignment of appropriate C atoms to individual signals (Table 2). Therefore, the products were identified as 8-chloroquino[3,2-b]benzo[1,4]thiazines (8-chlorobenzo[b]-1-azaphenothiazine).

**Scheme 3:**
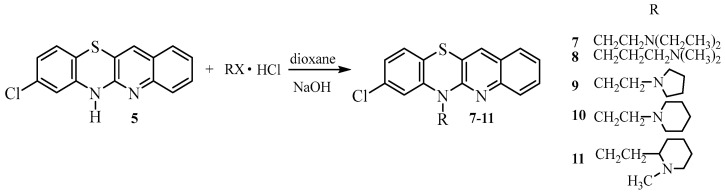
Synthesis of 6-dialkylaminoalkylquinobenzothiazines **7**–**11**.

The next step in the modification of the phenothiazine system was the introduction of N,N-dialkylaminoalkyl, N-acylaminoalkyl, N-sulfonylaminoalkyl groups, and 1,2,3-triazole substituents to the thiazine nitrogen atom in position 6. The N,N-dialkylaminoalkyl substituents were introduced in the N-alkylation reactions with hydrochlorides of selected acyclic and cyclic dialkylaminoalkyl chlorides in boiling dioxane in the presence of sodium hydroxide. As a result of such syntheses, the following was obtained five different 6-dialkylaminoalkyl derivatives **7**–**11** in 64–86% yield (Scheme 3).

**Scheme 4:**
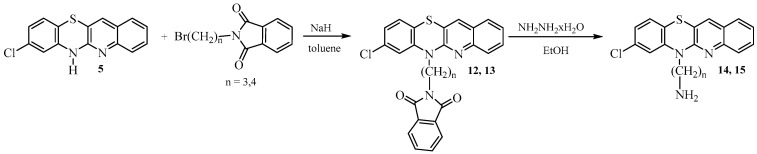
Synthesis of 6-aminoalkylquinobenzothiazines **14** and **15**.

Preparation of 6-substituted 8-chloroquinobenzothiazines with N-acylaminoalkyl and N-sulfonylaminoalkyl groups required a three-step synthesis. In the first stage, 6*H*-8-chloroquinobenzothiazine **5** was alkylated with phthalimidopropyl and phthalimidobutyl bromides in dry toluene in the presence of sodium hydride into the phthalimidoalkyl derivatives **12** and **13**. Next, these compounds underwent reactions with hydrazine hydrate in aqueous ethanol to give aminopropyl derivative **14** and aminobutyl derivative **15** with yields of 85 and 84%, respectively (Scheme 4).

**Scheme 5:**
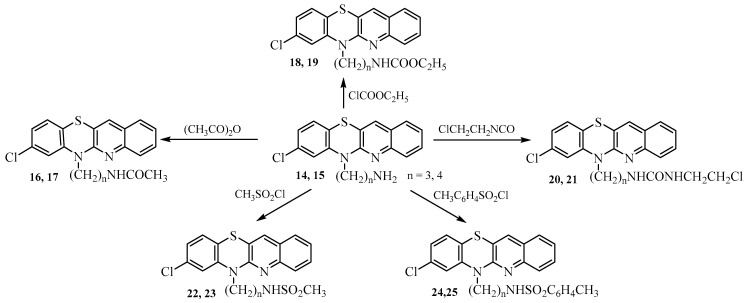
Scheme of the synthesis of N-acyl- (**16**–**21**) and N-sulfonyl- (**22**–**25**) 8-chloroquinobenzothiazines.

Aminoalkylquinobenzothiazines **14** and **15** were transformed into the N-acyl derivatives. The reactions with acetic anhydride, ethyl chloroformate, and 2-chloroethyl isocyanate gave two 8-chloro-6-acetylaminoalkylquinobenzothiazines **16**, **17**, two 8-chloro-6-ethoxycarbonylaminoalkylquinobenzothiazines **18** and **19**, and two 8-chloro-6-chloroethylureidoalkylquinobenzothiazines **20** and **21** (possessing a half-mustard unit) in 65–86% yield. Aminoalkylquinobenzothiazines **14** and **15** were also transformed into the N-sulfonyl derivatives. The reactions with methanesulfonyl and p-toluenesulfonyl chlorides led to the sulfonamide derivatives: two 8-chloro-6-methanesulfonylaminoalkyl- and two 8-chloro-6-p-toluenesulfonylaminoalkylquinobenzothiazines **22**–**25** in 74–77% yield (Scheme 5). The synthesis of these derivatives was based on the interesting potential of phenothiazines containing such substituents. As previous studies have shown, phenothiazines with a half-mustard unit have great biological potential. For 10-chloroethylureidoalkylphenothiazines, cytotoxic effects have been found against various 54–60 human cancer cell lines: leukemia, melanoma, small cell lung, colon, central nervous system, kidneys, breast, ovary, and prostate cancer. Significant anticancer activity against nine types of human cancer cells (leukemia, melanoma, small cell lung, colon, central nervous system, kidney, breast, ovarian, and prostate cancers) was also found for 6-chloroethylureidoethyldquinothiazine [38,40]. However, 9-chloro- and 9-methylthio-6-chloroethylureidoalkylquinobenzothiazines and 9-chloro-6-acetylaminopropylquinobenzothiazine showed very strong antiproliferative activity, no or low toxicity, and inhibited the production of TNF-α. They were also tested for anticancer activity against epidermoid carcinoma (A-431), lymphoma (L1210), and colorectal cell lines (SW-948). These compounds were as active as cisplatin [37,40]. However, quinobenzothiazine with a methanesulfone fragment showed cytotoxic activity against cancer cell lines A-431, L1210, SW-948, and CX-1, comparable to the reference cisplatin [37,40].

Due to the high pharmaceutical potential of substances containing the 1,2,3-triazole ring in their structure, we designed and synthesized a series of triazole derivatives of 8-chloroquinobenzothiazine [51,52,53,54,55,56]. These derivatives were obtained using 1,3-dipolar cycloaddition reactions between the 2-propynyl derivative of 8-chloroquinobenzothiazine **5** and some selected organic azides. Starting quinobenzothiazine **5** was transformed with 2-propynyl bromide into propynyl derivatives **26** according to the synthesis described in [37], and further using ‘click chemistry’ 1,3-dipolar cycloaddition (with selected azides, in toluene, in the presence of CuI as a catalyst) into substituted triazole derivatives of 8-chloroquinobenzothiazine **27**–**33**. Taking into account the significant biological activity of triazole systems with various phenyl and benzyl substituents [57,58,59], selected azides (phenyl azide, *p*-trifluoromethyphenyl azide, benzyl azide, *p*-fluorobenzyl azide, *p*-chlorobenzyl azide, p-cyanobenzyl azide, and phenylthiomethyl azide) were selected for 1,3-dipolar cycloaddition.

### 2.2. Biological Evaluation

#### 2.2.1. Cytotoxic Activity

**Scheme 6:**
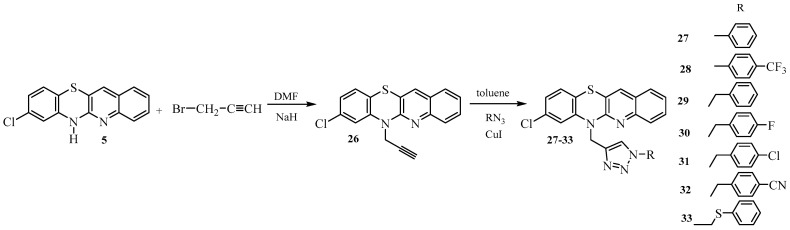
Scheme of the synthesis of 8-chloroquinobenzothiazines with 1,2,3-triazole substituents.

Our biological study aimed to assess the cytotoxicity of newly synthesized derivatives of chlorpromazine (Scheme 3, Scheme 4, Scheme 5 and Scheme 6) and their potential utility in cancer treatment. Initially, all derivatives were tested against two human carcinoma cell lines (A549—lung cancer, and MDA-MB-231—breast cancer) and a normal cell line HaCaT (immortalized human keratinocytes) to determine their IC_50_ values using the MTT (3-(4,5-dimethylthiazol-2-yl)-2,5 diphenyl tetrazolium bromide) method [60]. Comparisons to the IC_50_ values of the common chemotherapeutic agent, doxorubicin, are presented in Table 3.

Most derivatives exhibited moderate antiproliferative potency, with lower IC_50_ values observed in A549 cells compared to MDA-MB-231 cells. Among them, nine compounds (**5**, **8**, **9**, **11**, **20**, **21**, **23**, **25**, and **27**) showed promising activity against A549 cells without affecting HaCaT cells. Compounds **8**, **20**, **21**, and **25** demonstrated the highest cytotoxicity against A549 cells. The selectivity index (SI) for these compounds ranged from 0.15 to 10.7, which was higher or equal to that of doxorubicin (0.14–0.15). Based on these findings, nine compounds were further evaluated on three additional cancer cell lines (human pancreas cancer—MiaPaca-2, human prostate cancer—PC3, and human colon cancer—HCT116) using the MTT method (Table 4). Apart from the A549 cell line, HCT116 cells exhibited the highest sensitivity to the tested substances, while PC3 cells showed lower sensitivity.

It is important to note that while doxorubicin demonstrated high cytotoxicity against all cancer cell lines, it also affected normal cells. Compounds **8** and **23** displayed the highest selectivity index (39 and 143, respectively), with IC_50_ values of 1.6 ± 0.8 µM and 0.7 ± 0.08 µM, respectively, against HCT116 cells, while showing no cytotoxic effects on HaCaT cells.

Derivatives **5** and **11** showed moderate effectiveness against two tested cell lines in terms of antitumor activity (11.1 to 14.9 µM and 16.1 to 16.7 µM, respectively), but compound **11** exhibited notably higher cytotoxicity specifically against HCT-116 cells (7.7 µM) (Table 3 and Table 4).

Additionally, compound **21** was effective toward four cancerous cell lines (A549, MDA, MiaPaCa-2, and HCT116) at concentrations of 6.4–11.3 µM. However, despite relatively strong inhibitory effects on cancer cells, compound **21** also showed low selectivity (Table 3 and Table 4). All chlorpromazine derivatives tested against MiaPaCa-2, PC3, and HCT116 cell lines were less cytotoxic against normal cell lines than the reference doxorubicin (IC_50_ = 0.3). The lowest value of the IC_50_ parameter was determined for derivative **21** (IC_50_ = 1.1), while for quinobenzothiazines **9**, **20**, **23**, and **25**, IC_50_ > 100 was determined.

#### 2.2.2. In Vitro Antibacterial Activity

The antibacterial efficacy of newly synthesized derivatives **5**–**33** was assessed by initially screening them for their minimal inhibitory concentrations (MICs) [61].

The compounds were tested against standard Gram-positive bacteria, including various strains of *S. aureus* (NCTC 4163, ATCC 25923, ATCC 6538, and ATCC 29213) and *Staphylococcus epidermidis* (ATCC 12228 and ATCC 35984), as well as Gram-negative rods such as *Escherichia coli* (ATCC 25922) and *P. aeruginosa* (ATCC 15442).

The results showed that investigated compounds (**5**–**9**, **20**, **21**) exhibited the potential to moderate antibacterial potency, mainly against standard staphylococcal strains (Table 5).

In general, the most prominent activity against standard strains (except for P. aeruginosa) was observed for compound **21**, with MIC (minimum inhibitory concentration) values ranging from 2 to 8 µg/mL.

On the other hand, moderate activity (MIC range 8–16 µg/mL) against staphylococci was observed for compounds **5**–**9** and **20**. Moreover, compound **20** showed good activity against *S. epidermidis* strain ATCC 35984 (4 µg/mL). Additionally, compound **11** also exhibited moderate activity against one strain of *S. aureus*, NCTC 4163 (MIC 8 µg/mL), while its activity against the other three strains of *S. aureus* was very low (MIC >128 µg/mL). All tested compounds were inactive against the Gram-negative rods *P. aeruginosa* strain (ATCC 15442) and *E. coli*, except for compound **21**, which showed strong activity against *E. coli* (MIC 8 µg/mL) (Table 5).

The above results indicate that derivatives **8**, **20**, and **21** have both antibacterial and cytotoxic properties, which makes them promising compounds.

The most potent derivatives (**5**, **8**, **11**, **20**, **21**, **23**, and **25**) were selected for further investigations of their mechanisms of cytotoxic action.

#### 2.2.3. Mechanism of Cytotoxicity of Newly Synthesized Derivatives

**Figure 3:**
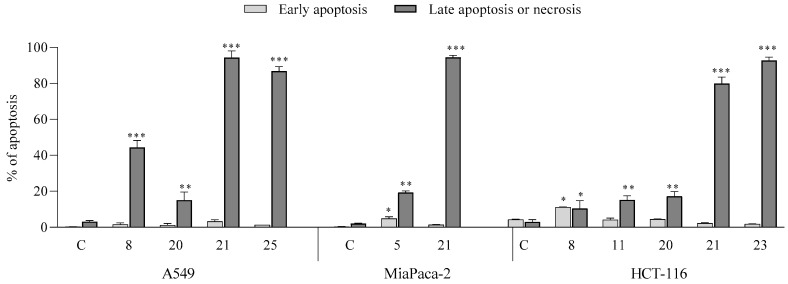
The effects of newly synthesized compounds **5**, **8**, **11**, **20**, **21**, **23**, and **25** on early and late apoptosis or necrosis were assessed in A549, MiaPaca-2, and HaCaT cell lines. Cells were treated with the compounds at their IC_50_ concentrations for 72 h, followed by staining with annexin V-FITC and PI, and analysis using flow cytometry. The results are presented as the percentage of cells in the early stage of apoptosis and the percentage of cells in the late stage of apoptosis or necrosis. *** *p* ≤ 0.0001, ** *p* ≤ 0.001, * *p* ≤ 0.01, as compared to the control (C).

**Figure 4:**
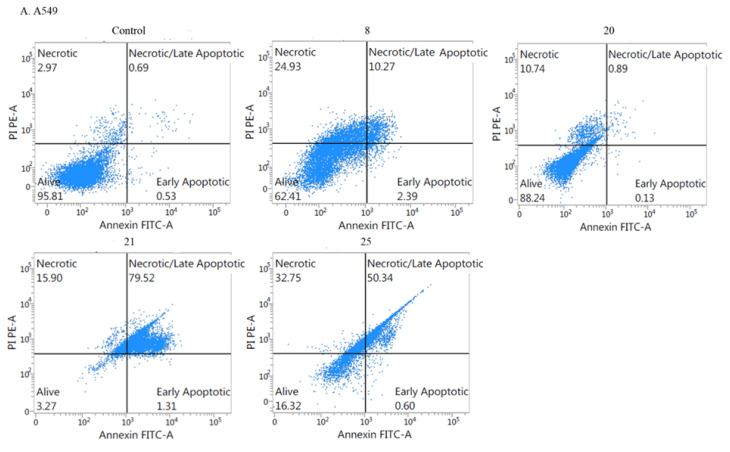

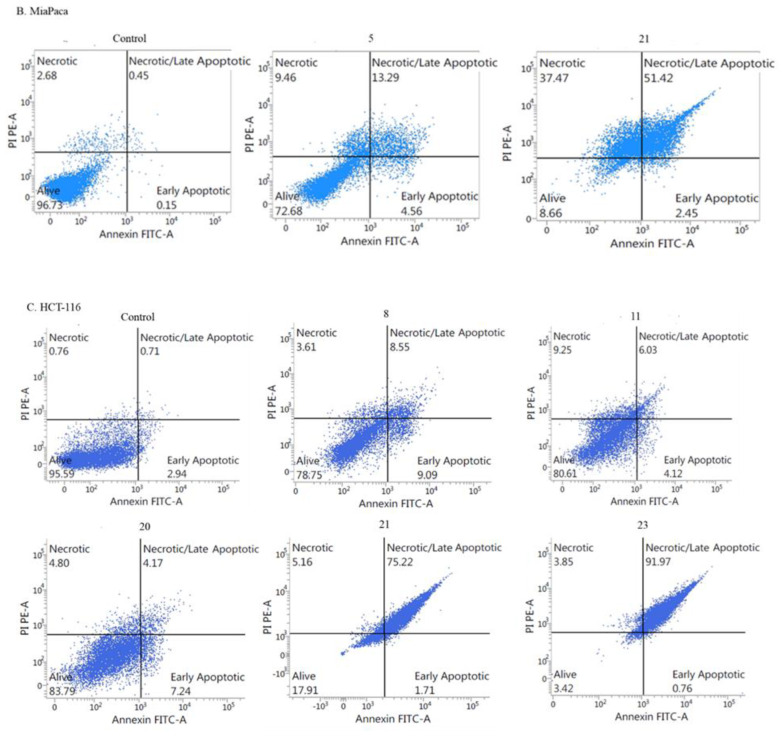
Representative results (%) as dot plots from apoptosis analysis of (**A**) A549, (**B**) MiaPaCa-2, and (**C**) HCT116 cancer cells treated with compounds **5**, **8**, **11**, **20**, **21**, **23**, and **25** determined by flow cytometry, using the Annexin VFITC/PI staining bioassay.

- Apoptotic activity:

Given the significant cytotoxic potential observed in the newly synthesized chlorpromazine-derived compounds, namely **5**, **8**, **11**, **20**, **21**, **23**, and **25**, against cancer cell lines such as A549 (lung cancer), MiaPaCa-2 (breast cancer), and HCT116 (colon cancer), with concentrations not exceeding 11 μM, these compounds were selected for further investigation to elucidate their mechanisms of biological action. Their ability to induce apoptosis or necrosis in cancer cells was assessed through flow cytometry analysis. As depicted in Figure 3 and Figure 4, the tested derivatives, when administered at their IC_50_ concentrations, exhibited proapoptotic properties in the selected cell lines compared to the untreated controls.

The obtained results indicated that the selected derivative **21** exhibited potent proapoptotic activity across all tested cancer cell lines (Figure 3 and Figure 4). For derivative **8**, a strong late apoptosis and necrosis-inducing effect was found in A549 (42% ± 0.76), whereas in HCT116 the same compound induced mainly early and late apoptosis/necrosis (9.09 ± 0.5 and 8.55 ± 0.33) (Figure 3). Furthermore, incubation with derivative **20** led to a significantly higher percentage of A549 and HCT116 cells in late apoptosis or necrosis (ranging from 15.2% to 17%) compared to the control. Additionally, a similar noticeable pro-apoptotic effect as compound **20** was observed with derivative **11** in HCT116 cells (15.64% in late apoptosis or necrosis). Compound **5** acted similarly, as it activated not only early but also late apoptosis/necrosis in MiaPaCa-2 cells, which accounted for 4.56% and 23%, respectively (Figure 3 and Figure 4B). The strongest late apoptosis and necrotic activity (82% ± 2.89) was found for **25** towards A549 cancer cells, while compound **23** exhibited a high percentage of late apoptosis/necrosis (93% ± 3.29) in HCT116 cells (Figure 3 and Figure 4C).

Our study confirmed the proapoptotic activity of selected compounds, especially in terms of inducing late apoptosis or necrosis in the A549, MiaPaCa-2, and HCT116 cancer cell lines (Figure 3).

- Induction of cell cycle arrest

The dysregulation of the cell cycle represents a hallmark of cancer cells, manifesting disruptions in various cellular pathways, particularly those governing the cell cycle and apoptosis. This dysregulation frequently enables cancer cells to evade crucial processes such as apoptosis or senescence, consequently leading to unchecked tumor proliferation and growth. Consequently, targeting this dysregulation has emerged as a promising therapeutic strategy in cancer treatment [62,63,64].

**Figure 5:**
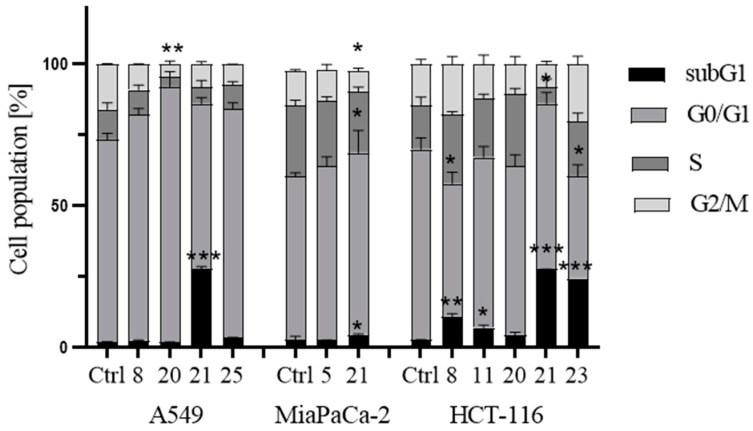
Flow cytometer analysis of cell cycle distribution in A549, MiaPaCa-2, and HCT116 cells after 24 h of incubation tested compounds (**5**, **8**, **11**, **20**, **21**, **23**, and **25**) with doses of IC_50_. The SubG1 fraction represents apoptotic and dead cells. Results are presented as the mean from four experiments with standard deviation (+/− SD). *** *p* < 0.001, ** *p* < 0.01, * *p* < 0.05, as compared to the control (Ctrl).

Therefore, the effects of new derivatives on the cell cycle were investigated to elucidate the inhibition mechanisms. The cells were exposed to IC_50_ concentrations of compounds **5**, **8**, **20**, **21**, **23**, and **25** for 24 h. It was found that compounds **5**, **8**, **20**, **21**, and **25** induced G0/G1 phase arrest in both cell lines (the A549 and MiaPaca-2, respectively) (Figure 3 and Figure 4A,B). A concomitant reduction in the number of cells in the S and G2/M phases was also observed (Figure 5).

**Figure 6:**
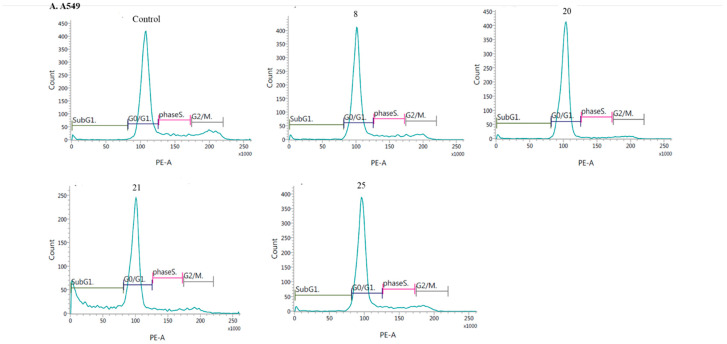

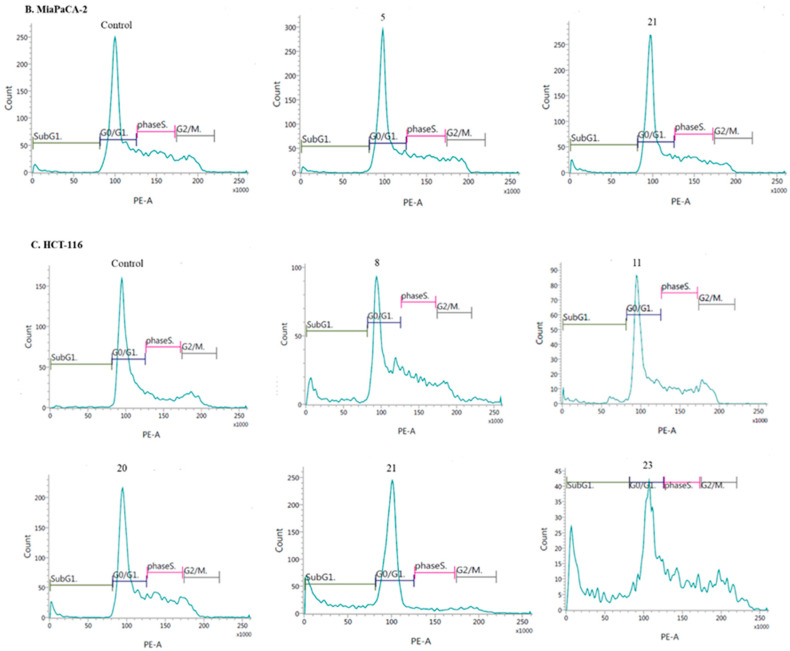
Flow cytometry analysis of cell cycle distribution after incubation of (**A**) A549, (**B**) MiaPaCa-2, and (**C**) HCT116 cancer cells with tested compounds (**5**, **8**, **11**, **20**, **21**, **23**, and **25**) at their IC_50_ concentration for 24 h. The SubG1 fraction represents apoptotic and dead cells. Results are presented as the mean from four experiments with standard deviation (+/− SD).

This increase in the G0/G1-phase cell population was mostly at the expense of G2/M cells. These results clearly suggest that the studied derivatives exert an antiproliferative effect by inducing cell cycle arrest at the S phase and, consequently, apoptosis. The addition of derivative **21** significantly increased the percentage of Sub G1 phase A549 cells when tested at IC_50_ (27.6%, *p* = 0.0001) and reduced the cell population in the G0/G1 and G2/M phases, compared to the cell cycle distribution monitored in untreated A459 cells (Figure 3 and Figure 4A). Upon treatment of HCT116 cells with compounds **8**, **21**, and **23**, a significant increase in the cell amount at the sub-G1 phase (5, 12, and 11-fold, respectively) was detected. Moreover, these three derivatives led to a significant increase in the number of cells accumulated in the S phase (approximately 2-fold) and in the G2/M phase (approximately 1.3-fold) as compared to the control. In addition, on exposure to compounds **11** and **20**, there was a small increase in the sub-G1 population (range 2- and 3-fold, respectively) and statistically significant growth at the S phase (range 20.4 to 25.81%). The compounds **11** and **20** decreased HCT116 cells at their G0/G1 phase, and arrested the cell population at the S phase (Figure 5 and Figure 6C). Furthermore, cell cycle distribution in HCT116 cells indicates proapoptotic action (Figure 3).

## 3. Methods and Materials

Melting points were determined in open capillary tubes on a Boetius melting point apparatus and were uncorrected. The standard NMR spectra were recorded on Bruker Avance spectrometers (Bruker, Billerica, MA, USA)(^1^H at 600 MHz, ^13^C at 150 MHz) in CDCl_3_ or DMSO-d_6_. Two-dimensional COSY, NOESY, HSQC, and HMBC spectra of selected compounds were recorded on a Bruker Avance spectrometer at 600 MHz, using COSYGPSW, NOESYGPPHSW, HSQCGPPH, and HMBCGP experiments. The HRMS spectra (EI—electroimpact ionization) were run on a Brucker Impact II (Bruker, Billerica, MA, USA). ^1^H NMR, ^13^C NMR and HRMS spectra are included in Supplementary Materials. Thin-layer chromatography was performed on aluminum oxide 60 F_254_ neutral (type E) (Merck 1.05581) with CH_2_Cl_2_ as eluents.

### 3.1. Synthesis of Compounds

#### General Procedure for the Synthesis of 8-Chloroquinobenzothiazine Derivatives

- Synthesis of 6*H*-8-chloroquino[3,2-b]benzo[1,4]thiazine **5**.

To a solution of 0.24 g (1 mmol) 3-bromo-2-chloroquinoline **2** in 5 mL dry DMF, 0.16 g (1 mmol) 2-amino-4-chlorothiophenol **1** was added. The reaction mixture was heated at reflux for 1 h. After cooling, the reaction mixture was poured into water (25 mL). The precipitate was filtered off, washed with water and, after drying, purified by crystallization from ethanol to give 6*H*-8-chloroquinobenzothiazine **5**. Yield: 79%. M.p.: 230–231 °C [50]. ^1^H NMR (DMSO-d_6_) δ: 6.86 (d, 1H, H-9, *J* = 6 Hz), 6.92 (d, 1H, H-7, *J* = 1.8 Hz), 7.04 (d, 1H, H-10, *J* = 8.4 Hz), 7.24–7.27 (m, 1H, H-2), 7.49–7.50 (m, 1H, H-1,H-3), 7.58 (d, 1H, H-4, *J* = 8.4 Hz), 7.84 (s, 1H, H-12), and 9.99 (s, 1H, NH). ^13^C NMR (75 MHz, DMSO-d_6_) δ: 114.66, 115.22, 115.25, 122.23, 124.48, 126.20, 126.64, 127.15, 127.55, 129.98, 132.04, 132.23, 140.87, 146.08, and 150.35. HR MS (ESI) calcd for C_15_H_10_N_2_S [M+H]^+^: 285.0253, found: 285.0256. R_f_ = 0.35 (Al_2_O_3_, CH_2_Cl_2_).

- Synthesis of 8-chloro-6-methylquino[3,2-b]benzo[1,4]thiazine **6**.

To a solution of 6*H*-8-chloroquino[3,2-b]benzo[1,4]thiazine **5** 0.14 g (0.5 mmol) **5** in dry DMF, (5 mL) NaH (0.12 g, 5 mmol, 60% NaH in mineral oil washed out with hexane) was added. The reaction mixture was stirred at room temperature for 0.5 h, then methyl iodide (0.05 mL, 0.75 mmol) was added and the stirring continued for 24 h. The reaction mixture was poured into water (25 mL). The resulting solid was filtered off, washed with water, and purified by column chromatography (aluminum oxide, CHCl_3_) to give 6-methyl-8-chloroquinobenzothiazine **2**.

Yield: 86%. M.p.: 120–121 °C. ^1^H NMR (600 MHz, CDCl_3_) δ: 3.61 (s, 3H, CH_3_), 6.94 (d, 1H, H7, *J* = 1.2 Hz), 6.96 (d, 1H, H9, *J* = 7.2 Hz), 7.05 (d, 1H, H10, *J* = 10.2 Hz), 7.32 (t, 1H, H2, *J* = 7.2 Hz), 7.55 (t, 1H, H3, *J* = 7.8 Hz), 7.56 (d, 1H, H1, *J* = 7.8 Hz), and 7.70 (s, 1H, H12), 7.79 (d, 1H, H4, *J* = 8.4 Hz). ^13^C NMR (75 MHz, CDCl_3_) δ: 29.73, 115.70, 118.19, 118.95, 122.48, 124.53, 125.99, 126.30, 127.23, 127.47, 129.33, 132.21, 133.60, 144.17, 145.74, and 152.77. HR MS (ESI) calcd for C_16_H_12_ClN_2_S [M+H]^+^: 299,0410, found: 299,0410. R_f_ = 0.77 (Al_2_O_3_, CH_2_Cl_2_).

- Synthesis of 8-chloro-6-dialkylaminoalkylquinobenzothiazines **7**–**11**.

A mixture of 6*H*-8-chloroquino[3,2-b]benzo[1,4]thiazine **5** (0.14 g, 0.5 mmol), sodium hydroxide (0.30 g, 7.5 mmol), and hydrochloride of dialkylaminoalkyl chloride (1.5 mmol, 2-diethylaminoethyl—0.26 g, 3-dimethylaminopropyl—0.24 g, 2-(1-pyrrolidyl)ethyl—0.26 g, 2-(1-piperidyl)ethyl—0.28 g, 2-(1-methyl-2-piperydinyl)ethyl—0.30 g) in dry dioxane (5 mL) was refluxed for 3 h. After cooling, the reaction mixture was poured into water (25 mL) and extracted with chloroform (3 × 10 mL). The combined extracts were washed with water to pH = 7 and dried over Na_2_SO_4_. Chloroform was evaporated in vacuo and the residue was purified by column chromatography (Al_2_O_3_, CHCl_3_) to give compounds **7**–**11**:

2-(8-Chloroquino[3,2-b]benzo[1,4]thiazin-6-yl)-N,N-diethylethan-1-amine (**7**):

Yield: 78%. Yellow oil. ^1^H NMR (CDCl_3_) δ: 1.16–1.20 (m, 6H, 2CH_3_), 2.68–2.72 (m, 4H, 2CH_2_), 2.79–2.82 (m, 2H, CH_2_), 4.22–4.24 (m, 2H, QbtNCH_2_), 6.80 (d, 1H, H-7, *J* = 7.8 Hz), 6.88 (d, 1H, H-9, *J* = 7.8 Hz), 7.06 (d, 1H, H-10, *J* = 8.4 Hz), 7.20 (t, 1H, H-2, *J* = 7.2 Hz), 7.42–7.46 (m, 2H, H-1, H-3), 7.51 (s, 1H, H-12), and 7.61 (d, 1H, H-4, *J* = 10.2 Hz). ^13^C NMR (75 MHz, CDCl_3_) δ: 12.07, 47.86, 48.11, 116.03, 117.73, 118.21, 122.25, 124.72, 125.94, 127.18, 127.39, 129.20, 129.51, 131.62, 133.52, 142.67, 145.66, and 151.23. HR MS (ESI) calcd for C_21_H_23_ClN_3_S [M+H]^+^: 384.1301, found: 384.1302. R_f_ = 0.24 (Al_2_O_3_, CH_2_Cl_2_).

3-(8-Chloroquino[3,2-b]benzo[1,4]thiazin-6-yl)-N,N-dimethylpropan-1-amine (**8**):

Yield: 86%. M.p.: 73–75 °C. ^1^H NMR (CDCl_3_) δ: 2.06–2.08 (m, 2H, CH_2_), 2.35 (s, 6H, 2CH_3_), 2.52 (t, 2H, CH_2_, *J* = 7.2 Hz), 4.22–4.24 (m, 2H, QbtNCH_2_), 6.88 (d, 1H, H-7, *J* = 8.4 Hz), 6.96 (d, 1H, H-9, *J* = 8.4 Hz), 7.00 (s, 1H, H-10), 7.27 (t, 1H, H-2, *J* = 7.8 Hz), 7.49 (d, 1H, H-1, *J* = 7.8 Hz), 7.50 (t, 1H, H-3, *J* = 7.8 Hz), 7.57 (s, 1H, H-12), and 7.71 (d, 1H, H-4, *J* = 8.4 Hz). ^13^C NMR (75 MHz, CDCl_3_) δ: 24.25, 44.09, 45.63, 57.36, 115.92, 117.95, 118.58, 122.23, 124.39, 126.04, 126.14, 127.26, 127.45, 129.16, 131.72, 133.45, 142.73, 145.68, and 151.60. HR MS (ESI) calcd for C_20_H_21_ClN_3_S [M+H]^+^: 370.1145, found: 370.1151. R_f_ = 0.15 (Al_2_O_3_, CH_2_Cl_2_).

8-Chloro-6-(2-(pyrrolidin-1-yl)ethyl)-quino[3,2-b]benzo[1,4]thiazine (**9**):

Yield: 79%. Yellow oil. ^1^HNMR (CDCl_3_) δ: 1.88–1.92 (m, 4H, 2CH_2_), 2.88–2.92 (m, 4H, 2CH_2_), 3.07–3.11 (m, 2H, CH_2_), 4.38–2.40 (m, 2H, QbtNCH_2_), 6.80 (d, 1H, H-7, *J* = 7.8 Hz), 6.88 (d, 1H, H-9, *J* = 8.4 Hz), 7.03 (d, 1H, H-10, *J* = 1.8 Hz), 7.20 (t, 1H, H-2, *J* = 7.8 Hz), 7.41- 7.43 (m, 2H, H-1, H-3), 7.50 (s, 1H, H-12), and 7.62 (d, 1H, H-4, *J* = 8.4 Hz). ^13^C NMR (75 MHz, CDCl_3_) δ: 23.53, 43.84, 51.54, 54.22, 115.95, 117.90, 118.35, 122.68, 124.68, 126.12, 126.22, 127.35, 127.35, 129.32, 131.89, 133.80, 142.36, 145.46, and 151.15. HR MS (ESI) calcd for C_21_H_21_ClN_3_S [M+H]^+^: 382.1145, found: 382.1145. R_f_ = 0.14 (Al_2_O_3_, CH_2_Cl_2_).

8-Chloro-6-(2-(piperidin-1-yl)ethyl)-quino[3,2-b]benzo[1,4]thiazine (**10**):

Yield: 76%. M.p.: 99–100 °C. ^1^HNMR (CDCl_3_) δ: 1,51 (m, 2H, CH_2_), 1.68–1.70 (m, 4H, 2CH_2_), 2.58–2.62 (m, 4H, 2CH_2_), 2.82–2.85 (m, 2H, CH_2_), 4.35 (m, 2H, QbtNCH_2_), 6.88 (d, 1H, H-7, *J* = 8.4 Hz), 6.96 (d, 1H, H-9, *J* = 8.4 Hz), 7.20 (d, 1H, H-10), 7.49–7.52 (m, 2H, H-1, H-3), 7.57 (s, 1H, H-12), and 7.71 (d, 1H, H-4, *J* = 7.8 Hz). ^13^C NMR (75 MHz, CDCl3) δ: 24.86, 26.07, 44.18, 55.10, 55.32, 116.26, 117.85, 118.41, 122.26, 124.42, 126.07, 126.19, 127.17, 127.47, 129.16, 131.63, 133.52, 142.79, 145.65, and 151.39. HR MS (ESI) calcd for C_22_H_22_ClN_3_S [M+H]^+^: 396.1310, found: 396.1310. R_f_ = 0.13 (Al_2_O_3_, CH_2_Cl_2_).

8-Chloro-6-(2-(1-methylpiperidin-2-yl)ethyl)-quino[3,2-b]benzo[1,4]thiazine (**11**):

Yield: 64%. M.p.: 143–144 °C. ^1^H NMR (CDCl_3_) d: 1.64–1.68 (m, 4H, 2CH_2_), 1.83–1.87 (m, 2H, CH_2_), 2.09–2.18 (m, 4H, 2CH_2_), 2.48 (s, 3H, CH_3_), 2.90–2.94 (m, 1H, CH), 4.19–4.23 (m, 2H, CH_2_), 6.87 (d, 1H, H-7, *J* = 8.4 Hz), 6.91 (d, 1H, H-9, *J* = 8.4 Hz), 6.96 (d, 1H, H-10), 7.27 (t, 1H, H-2, *J* = 8.4 Hz), 7.49–7.51 (m, 2H, H-1, H-3), 7.57 (s, 1H, H-12), and 7.69 (d, 1H, H-4, *J* = 8.4 Hz). ^13^C NMR (75 MHz, CDCl_3_) δ: 24.36, 25.86, 28.63, 31.03, 43.01, 43.44, 57.13, 62.59, 115.59, 117.76, 118.38, 122.11, 124.38, 126.03, 126.12, 127.24, 127.37, 129.15, 131.58, 133.38, 142.67, 145.72, and 151.24. HR MS (ESI) calcd for C_23_H_25_ClN_3_S [M+H]^+^: 410.1458, found: 410.1468. R_f_ = 0.11 (Al_2_O_3_, CH_2_Cl_2_).

- Synthesis of 8-chloro-6-phthalimidoalkylquinobenzothiazines **12** and **13**.

To a stirred solution of 6*H*-8-chloroquino[3,2-b]benzo[1,4]thiazine **5** (0.14 g, 0.5 mmol) in dry toluene, (5 mL) NaH (0.12 g, 10 mmol, washed out with hexane) was added. The mixture was refluxed for 30 min and a solution of N-(bromoalkyl)phthalimide [1.1 mmol, N-(3-bromopropyl)phthalimide 0.30 g, N-(4-bromobutyl)phthalimide 0.31 g] in dry toluene (5 mL) was added. The mixture was refluxed for 24 h. Next, toluene was evaporated in vacuo and the residue was extracted with CHCl_3_ (2.5 mL). The extract was concentrated and purified by column chromatography (silica gel, CHCl_3_) to give compounds **12** or **13**:

2-(3-(8-Chloroquino[3,2-b]benzo[1,4]thiazin-6-yl)propyl)isoindoline-1,3-dione (**12**):

Yield: 85%. M.p.: 168–169 °C. ^1^H NMR (CDCl_3_) δ: 2.33–2.35 (m, 2H, CH_2_), 3.94 (t, 2H, NCH_2_, *J* = 6.6 Hz), 4.32 (t, 2H, QbtNCH_2_, *J* = 6.6 Hz), 6.84 (s, 1H, H-7), 6.87 (d, 1H, H-9, *J* = 7.8 Hz), 6.95 (d, 1H, H-10, *J* = 8.4 Hz), 7.26 (t, 1H, H-2, *J* = 7.8 Hz), 7.45 (t, 1H, H-3, *J* = 7.8 Hz ), 7.47 (d, 1H, H-1, *J* = 7.8 Hz), 7.56–7.58 (m, 2H, H-4, H-12), 7.69–7.70 (m, 2H, 2H phthal.), and 7.79–7.80 (m, 2H, 2H phthal.). ^13^C NMR (75 MHz, CDCl_3_) δ: 25.35, 29.83, 35.92, 115.81, 119.09, 122.56, 123.19, 123.36, 125.99, 126.10, 127.29. 127.46, 129.28, 130.92, 132.00, 132.07, 133.50, 133.88, 142.44, 151.59, 168.29, and 168.39. HR MS (ESI) calcd for C_26_H_19_ClN_3_O_2_S [M+H]+: 472,0887, found: 472,0871. R_f_ = 0.68 (Al_2_O_3_, CH_2_Cl_2_).

2-(4-(8-Chloroquino[3,2-b]benzo[1,4]thiazin-6-yl)butyl)isoindoline-1,3-dione (**13**):

Yield: 84%. M.p.: 148–149 °C. ^1^H NMR (CDCl_3_) δ: 1.91–1.93 (m, 4H, 2CH_2_), 3.79–3.83 (m, 2H, NCH_2_), 4.31–4.33 (m, 2H, QBTNCH_2_), 6.89 (d, 1H, H-9, *J* = 7.8 Hz), 6.91 (s, 1H, H-7), 6.98 (d, 1H, H-10, *J* = 7.8 Hz), 7.29 (d, 1H, H-2, *J* = 5.4 Hz), 7.50–7.52 (m, 2H, H-1, H-3), 7.64 (s, 1H, H-12), 7.71–7.73 (m, 2H, H phthal.), 7.75 (d, 1H, H-4, *J* = 8.4 Hz), and 7,83–7.84 (m, 2H, H phthal.). ^13^C NMR (75 MHz, CDCl_3_) δ: 23.66. 26.00, 37.61, 45.45, 116.42, 119.31, 122.80, 123.24, 123.30, 124.74, 125.88, 126.18, 126.86, 127.51, 129.60, 132.05, 132.57, 133.60, 133.91, 134.03, 142.35, 151.91, and 168.45. HR MS (ESI) calcd for C_27_H_21_ClN_3_O_2_S [M+H]^+^: 486,1043, found: 486.1040. R_f_ = 0.74 (Al_2_O_3_, CH_2_Cl_2_).

- Synthesis of 8-chloro-6-aminoalkylquinobenzothiazines **14** and **15**.

To a boiling solution of compounds **12** or **13** (0.5 mmol) in EtOH, (25 mL) 80% aqueous solution of hydrazine (0.1 mL, 2.5 mmol) was added. The mixture was refluxed for 2 h. After cooling, the reaction mixture was acidified to pH ≈ 2 with conc. hydrochloric acid and evaporated. Water (10 mL) was added to the residue, and the resulting solid was filtered off and washed with 10% hydrochloric acid. The combined filtrates were alkalized with 20% aqueous NaOH solution to pH ≈ 10 and the resulting solid was filtered off, washed with water, dried, and purified by column chromatography (SiO_2_, CHCl_3_-EtOH 10:1) to give compounds **14** or **15**:

3-(8-Chloroquino[3,2-b]benzo[1,4]thiazin-6-yl)propan-1-amine (**14**):

Yield: 76%. M.p.: 89–90 °C. ^1^H NMR (CDCl_3_) δ: 2.10–2.12 (m, 2H, CH_2_), 3.00–3.02 (m, 2H, NCH_2_), 4.27–4.32 (m, 2H, NCH_2_), 6.90 (d, 1H, H-9, *J* = 7.8 Hz), 6.95 (s, 1H, H-7), 6.99 (d, 1H, H-10, *J* = 7.8 Hz), 7.26 (t, 1H, H-2, *J* = 6.6 Hz), 7.47–7.50 (m, 2H, H-1, H-3), 7.61 (s, 1H, H-12), and 7.73 (d, 1H, H-4, *J* = 8.4 Hz). HR MS (ESI) calcd for C_18_H_17_ClN_3_S [M+H]^+^: 342.0832, found: 342,0844. R_f_ = 0.01 (Al_2_O_3_, CH_2_Cl_2_).

4-(8-Chloroquino[3,2-b]benzo[1,4]thiazin-6-yl)butan-1-amine (**15**):

Yield: 70%. M.p.: 127–128 °C. ^1^H NMR (DMSO-d_6_) δ: 1.71–1.74 (m, 2H, CH_2_), 1.78–1.72 (m, 2H, CH_2_), 2.85–2.87 (m, 2H, NCH_2_), 4.22–4.24 (m, 2H, QbtNCH_2_), 7.05 (d,1H, H-9, *J* = 7.8 Hz), 7.16 (s, 1H, H-7), 7.22 (d, 1H, H-10, *J* = 7.8 Hz), 7.34 (t, 1H, H-2), 7.57 (t, 1H, H-3, *J* = 7.8), 7.69 (d, 1H, H-1, J = 7.8), 7.79 (s, 1H, H-12), and 8.02 (d,1H, H-4, *J* = 8.4). ^13^C NMR (75 MHz, CDCl_3_) δ: 23.65, 25.17, 39.07, 44.39, 116.48, 117.63, 118.96, 123.07, 125.14, 126.23, 127.05, 127.46, 128.44, 130.06, 132.97, 133.17, 142.58, 145.38, and 151.72. HR MS (ESI) calcd for C_19_H_19_ClN_3_S [M+H]^+^: 356,0988, found: 356.0987. R_f_ = 0.02 (Al_2_O_3_, CH_2_Cl_2_).

- Synthesis of 8-chloro-6-acetylaminoalkylquinobenzothiazines **16**–**25**.

To a suspension of aminoalkylquinothiazines **14** or **15** (0.5 mmol) in pyridine (5 mL), acetic anhydride (3 mL, 32 mmol) was added and the mixture was stirred at rt for 24 h. The reaction mixture was poured into water (10 mL) and the resulting solid was filtered off, washed with water, air dried, and purified by column chromatography (Al_2_O_3_, CHCl_3_) to give compounds **16** or **17**:

N-(3-(8-Chloroquino[3,2-b]benzo[1,4]thiazin-6-yl)propyl)acetamide (**16**):

Yield: 84%. M.p.: 179–180 °C. ^1^H NMR (CDCl_3_) δ: 2.02 (s, 3H, CH_3_), 2.07–2.11 (m, 2H, CH_2_), 3.44–3.47 (m, 2H, NCH_2_), 4.32–4.34 (m, 2H, QbtNCH_2_), 6.17 (s, 1H, NH), 6.92 (s, 1H, H-7), 6.99 (d, 1H, H-9, *J* = 6.6 Hz), 7.01 (d, 1H, H-10, *J* = 6.8 Hz), 7.31 (t, 1H, H-2, *J* = 7.8 Hz), 7.53–7.55 (m, 2H, H-1, H-3), 7.65 (s, 1H, H-12), and 7.71 (d, 1H, H-4, *J* = 8.4 Hz). ^13^C NMR (75 MHz, CDCl_3_) δ: 23.43, 26.38, 37.43, 42.58, 115.94, 117.98, 118.84, 122.73, 124.75, 126.14, 126.35, 126.93, 127.51, 129.54, 132.24, 133.60, 142.28, 145.34, 151.84, and 170.19. HR MS (ESI) calcd for C_20_H_19_ClN_3_OS [M+H]^+^: 384,0937, found: 384,0931. R_f_ = 0.25 (Al_2_O_3_, CH_2_Cl_2_).

N-(4-(8-Chloroquino[3,2-b]benzo[1,4]thiazin-6-yl)butyl)acetamide (**17**):

Yield: 86%. M.p.: 149–150 °C. ^1^H NMR (CDCl_3_) δ: 1.72–1.77 (m, 2H, CH_2_), 1.92–1.96 (m, 2H, CH_2_), 1.98 (s, 3H, CH_3_), 3.38–3.41 (m, 2H, NCH_2_), 4.35–4.36 (m, 2H, QbtNCH_2_), 6.95–6.98 (m, 2H, H-7, H-8), 7.04 (d, 1H, H-10, *J* = 8.4 Hz), 7.35 (t, 1H, H-2, *J* = 7.2 Hz), 7.57–7.58 (m, 2H, H-1, H-3), 7.72 (s, 1H, H-12), and 7.93 (m, 1H, H-4). ^13^C NMR (75 MHz, CDCl_3_) δ: 23.28, 23.91, 26.40, 29.72, 38.82, 117.79, 119.87, 122.47, 124.66, 125.31, 126.20, 126.56, 127.86, 128.40, 131.32, 132.83, 134.32, 141.50, 141.85, 151.37, and 170.57. HR MS (ESI) calcd for C_21_H_21_ClN_3_OS [M+H]^+^: 398,1094, found: 398.1092. R_f_ = 0.27 (Al_2_O_3_, CH_2_Cl_2_).

- Synthesis of 8-chloro-6-etoxycarbonylaminoalkylquinobenzothiazines **18** and **19**.

To a stirred solution of aminoalkylquinobenzothiazines **14** or **15** (0.5 mmol) in a mixture of CH_2_Cl_2_ (5 mL) and 10% Na_2_CO_3_ solution (5 mL), a solution of ethyl chloroformate (0.65 mL, 0.65 mmol) in CH_2_Cl_2_ (3 mL) was added. The mixture was stirred at rt for 24 h. The organic phase was separated and the aqueous phase was extracted with CH_2_Cl_2_ (2 × 5 mL). The combined extracts were washed with water (2 × 10 mL) and dried over Na_2_SO_4_. The drying agent was filtered off and the filtrate was evaporated. The resulting residue was purified by column chromatography (Al_2_O_3_, CHCl_3_) to give compounds **18** or **19**:

Ethyl(3-(8-chloroquino[3,2-b]benzo[1,4]thiazin-6-yl)propyl)carbamate (**18**):

Yield: 78%. M.p.: 130–131 °C. ^1^H NMR (CDCl_3_) δ: 1.29–1.31 (t, 3H, CH_3_), 2.09–2.11 (m, 2H, CH_2_), 3.40–4.42 (m, 2H, NCH_2_), 4.16–4.19 (m, 2H, CH_2_), 4.28–4.29 (m, 2H, NCH_2_), 5.75 (s, 1H, NH), 6.90–6.91 (m, 2H, H-7, H-9), 6.98 (d, 1H, H-10, *J* = 7.8 Hz), 7.30 (t, 1H, H-2, *J* = 8.4 Hz), 7.52–7.53 (m, 2H, H-1, H-3), 7.62 (s, 1H, H-12), and 7.85 (d, 1H, H-4, *J* = 7.8 Hz). ^13^C NMR (75 MHz, CDCl_3_) δ: 26.76, 27.34, 38.79, 42.69, 60.74, 115.70, 117.79, 118.56, 122.56, 124.66, 126.12, 126.20, 127.12, 127.37, 129.39, 132.05, 133.48, 142.34, 145.43, 151.54, and 156.84. HR MS (ESI) calcd for C_21_H_21_ClN_3_O_2_S [M+H]^+^: 414,1043, found: 414,1055. R_f_ = 0.56 (Al_2_O_3_, CH_2_Cl_2_).

Ethyl(4-(8-chloroquino[3,2-b]benzo[1,4]thiazin-6-yl)butyl)carbamate (**19**):

Yield: 81%. M.p.: 138–139 °C. ^1^H NMR (CDCl3) δ: 1.23–1.27 (d, 3H, CH_3_), 1.72–1.76 (m, 2H, CH_2_), 1.92–1.95 (m, 2H, CH_2_), 3.31–3.33 (m, 2H, NCH_2_), 4.10–4.114 (m, 2H, CH_2_), 4.32 (m, 2H, QbtNCH_2_), 4.91–4.93 (m, 1H, NH), 6.93–6.95 (m, 2H, H-7, H-9), 7.03 (d, 1H, H-10, J = 7.8 Hz), 7.33 (d, 1H, H-2), 7.55–7.56 (m, 2H, H-1, H-3), 7.69 (s, 1H, H-12), and 7,91 (d, 1H, H-4, *J* = 7.8 Hz). ^13^C NMR (75 MHz, CDCl_3_) δ: 14.66, 23.72, 27.07, 40.23, 40.36, 60.69, 116.01, 117.58, 119.82, 122.73, 123.16, 125.40, 125.86, 126.41, 127.61, 127.80, 130.07, 130.92, 134.16, 141.57, 151.58, and 156.75. HR MS (ESI) calcd for C_22_H_23_ClN_3_O_2_S^+^: 428.1200, found: 428.1198. R_f_ = 0.65 (Al_2_O_3_, CH_2_Cl_2_).

- Synthesis of 8-chloro-6-chloroethylureidoalkylquinobenzothiazines **20** and **21**.

To a stirred solution of 6-aminoalkyldiquinothiazines **14** or **15** (0.5 mmol) in ethanol (10 mL) at 0 °C, 2-chloroethyl isocyanate (0.08 mL, 1 mmol) was added. The mixture was stirred at 0 °C for 1 h and at rt for 24 h. After evaporation of EtOH in vaccuo, the residue was purified by column chromatography (Al_2_O_3_, CHCl_3_) to give compounds **20** or **21**:

1-(3-(8-Chloroquino[3,2-b]benzo[1,4]thiazin-6-yl)propyl)-3-(2-chloroethyl)urea (**20**):

Yield: 73%. M.p.: 165–166 °C. ^1^H NMR (CDCl_3_) δ: 2.09–2.13 (m, 2H, CH_2_), 3.41–3.42 (m, 2H, CH_2_), 3.53–3.56 (m, 2H, NCH_2_), 3.61–3.63 (m, 2H, NCH_2_), 4.33–4.36 (m, 2H, NCH_2_), 6.93–6.94 (m, 2H, H-7, H-9), 7.03 (d, 1H, H-10, *J* = 7.8 Hz), 7.32 (t, 1H, H-2, *J* = 7.2 Hz), 7.55–7.58 (m, 2H, H-1, H-3), 7.68 (s, 1H, H-12), and 7.80 (d, 1H, H-4, *J* = 8.4 Hz). ^13^C NMR (75 MHz, CDCl_3_) δ: 26.68, 26.96, 37.62, 43.43, 48.27, 119.38, 120.89, 123.09, 124.51, 125.80, 126.95, 127.11, 128.03, 128.39, 128.45, 133.30, 133.44, 135.35, 138.46, 139.73, and 159.21. HR MS (ESI) calcd for C_21_H_21_Cl_2_N_4_OS [M+H]^+^: 447.0831, found: 447,0827. R_f_ = 0.07 (Al_2_O_3_, CH_2_Cl_2_).

1-(4-(8-Chloroquino[3,2-b]benzo[1,4]thiazin-6-yl)butyl)-3-(2-chloroethyl)urea (**21**):

Yield: 65%. M.p.: 169–170 °C. ^1^H NMR (DMSO-d_6_) δ: 1.89 (t, 2H, CH_2_, *J* = 7.2 Hz), 3.18–3.19 (m, 2H, CH_2_), 3.30–3.32 (m, 4H, 2CH_2_), 3.55–3.57 (m, 2H, CH_2_), 4.20–4.22 (m, 2H, CH_2_), 6.18–6.23 (m, 1H, 1NH_2_), 6.24–6.25 (m, 1H, 1NH_2_), 7.04 (d, 1H, H-9, *J* = 6.6 Hz), 7.11 (s, 1H, H-7), 7.21 (d, 1H, H-10, *J* = 8.4 Hz), 7.34 (t, 1H, H-2, *J* = 7.2 Hz), 7.55 (t, 1H, H-3, *J* = 7.2 Hz), 7.68–7.69 (m, 2H, H-1, H-4), and 7.99 (s, 1H, H-12). ^13^C NMR (75 MHz, DMSO-d_6_) δ: 27.34, 37.69, 41.94, 43.12, 45.01, 55.39, 116.30, 117.60, 118.91, 122.94, 125.08, 126.21, 127.00, 127.49, 128.38, 130.00, 132.84, 133.09, 142.64, 145.43, 151.64, and 158.31. HR MS (ESI) calcd for C_22_H_23_Cl_2_N_4_OS [M+H]^+^: 461.0970, found: 461.0942. R_f_ = 0.10 (Al_2_O_3_, CH_2_Cl_2_).

- Synthesis of 8-chloro 6-methanesulfonylaminoalkylquinobenzothiazines **22** and **23**.

To a stirred solution of aminoalkyldiquinothiazines **14** or **15** (0.5 mmol) in a mixture of CH_2_Cl_2_ (5 mL) and 10% Na_2_CO_3_ solution (7 mL), a solution of methanesulfonyl chloride (0.06 mL, 0.75 mmol) was added. The mixture was stirred at rt for 24 h. The organic phase was separated and the aqueous phase was extracted with CH_2_Cl_2_ (2 × 5 mL). The combined extracts were washed with water (2 × 10 mL) and dried over Na_2_SO_4_. The drying agent was filtered off and the filtrate was evaporated. The resulting residue was purified by column chromatography (Al_2_O_3_, CHCl_3_) to give compounds **22** or **23**:

N-(3-(8-Chloroquino[3,2-b]benzo[1,4]thiazin-6-yl)propyl)methanesulfonamide (**22**):

Yield: 71%. M.p.: 138–139 °C. ^1^H NMR (CDCl_3_) δ: 2.16–2.20 (m, 2H, CH_2_), 2,89 (s, 3H, CH_3_), 3.32–3.34 (m, 2H, NCH_2_), 4.29–4.41 (m, 2H, NCH_2_), 5,91 (s, 1H, NH), 6.94–6.99 (m, 2H, H-9, H-10), 7.01 (s, 1H, H-7), 7.34 (t, 1H, H-2, *J* = 7.8 Hz), 7.54 (d, 1H, H-1, *J* = 7.8 Hz), 7.58 (t, 1H, H-3, *J* = 7.2 Hz), 7.66 (s, 1H, H-12), and 7.91 (d, 1H, H-4). ^13^C NMR (75 MHz, CDCl_3_) δ: 28.99, 30.57, 38.89, 67.77, 120.14, 121.06, 121.21, 122.11, 124.09, 124.54, 126.80, 126.99, 127.88, 128.38, 129.50, 133.41, 153.23, 138.51, and 151.51. HR MS (ESI) calcd for C_19_H_19_ClN_3_O_2_S_2_ [M+H]^+^: 420.0607, found: 420.0607. R_f_ = 0.13 (Al_2_O_3_, CH_2_Cl_2_).

N-(4-(8-Chloroquino[3,2-b]benzo[1,4]thiazin-6-yl)butyl)methanesulfonamide (**23**):

Yield: 76%. M.p.: 169–170 ⁰C. ^1^H NMR (CDCl^3^) δ: 1.78–1.80 (m, 2H, CH_2_), 1.94–1.96 (m, 2H, CH_2_), 2.96 (s, 3H, CH_3_), 3.23–3.27 (m, 2H, NCH_2_), 4.30–4.32 (m, 2H, QbtNCH_2_), 6.91 (s, 1H, H-7), 6.93 (d, 1H, H-9, J = 7.8 Hz), 7.00–7.01 (d, 1H, H-10), 7.32 (s, 1H, H-7), 7.52–7.55 (m, 2H, H-1, H-3), 7.70 (s, 1H, H-12), and 7.90–7.91 (m, 1H, H-4). ^13^C NMR (75 MHz, CDCl_3_) δ: 23.38, 27.43, 40.33, 42.67, 42.78, 116.43, 116.84, 118.57, 119.28, 123.13, 124.67, 125.07, 125.84, 126.31, 127.61, 127.96, 129.96, 133.72, 142.17, and 151.65. HR MS (ESI) calcd for C_20_H_21_ClN_3_O_2_S_2_ [M+H]^+^: 434.0764, found: 434.0763. R_f_ = 0.14 (Al_2_O_3_, CH_2_Cl_2_).

- Synthesis of 8-chloro-6-p-toluenesulfonylaminolkylquinobenzothiazines **24** and **25**.

To a stirred solution of aminoalkyldiquinothiazines **14** or **15** (0.5 mmol) in a mixture of CH_2_Cl_2_ (5 mL) and 10% Na_2_CO_3_ solution (7 mL), a solution of p-toluenesulfonyl chloride (0.14 g, 0.75 mmol) in CH_2_Cl_2_ (6 mL) was added. The mixture was stirred at rt for 24 h. The organic phase was separated and the aqueous phase was extracted with CH_2_Cl_2_ (2 × 5 mL). The combined extracts were washed with water (2x10 mL) and dried over Na_2_SO_4_. The drying agent was filtered off and the filtrate was evaporated. The resulting residue was purified by column chromatography (Al_2_O_3_, CHCl_3_) to give compounds **24** or **25**:

N-(3-(8-Chloroquino[3,2-b]benzo[1,4]thiazin-6-yl)propyl)-4-methylbenzenesulfonamide (**24**):

Yield: 68% M.p.: 121–122 °C. ^1^H NMR (CDCl_3_) δ: 2.02–2.05 (m, 2H, CH_2_), 2.39 (s, 3H, CH_3_), 3.14–3.15 (m, 2H, NCH_2_), 4.22–4.24 (m, 2H, NCH_2_), 6.09 (s, 1H, NH), 6.86 (s, 1H, H-7), 6.93 (d, 1H, H-9, *J* = 8.4 Hz), 6.99 (d, 1H, H-10, *J* = 8.4 Hz), 7.18 (d, 2H, 2H_ph_, *J* = 7.8 Hz), 7.37 (t, 1H, H-2, *J* = 7.8 Hz), 7.56 (d, 1H, H-1, *J* = 7.8 Hz), 7.61 (t, 1H, H-3, *J* = 7.8 Hz), and 7.64–7.65 (m, 4H, 2H_ph_, H-4, H-12). ^13^C NMR (75 MHz, CDCl_3_) δ: 21.52, 26.44, 40.78, 42.98, 116.23, 118.74, 123.28, 125.30, 125.87, 126.15, 126.27, 127.01, 127.47, 129.63, 130.32, 133.00, 133.67, 136.80, 141.50, 143.16, and 151.29. HR MS (ESI) calcd for C_25_H_23_ClN_3_O_2_S_2_ [M+H]^+^: 496.0920, found: 496.0924. R_f_ = 0.26 (Al_2_O_3_, CH_2_Cl_2_).

N-(4-(8-Chloroquino[3,2-b]benzo[1,4]thiazin-6-yl)butyl)-4-methylbenzenesulfonamide (**25**):

Yield: 73%. M.p.: 148–149 °C. ^1^H NMR (CDCl_3_) δ: 1.67–1.72 (m, 2H, CH_2_), 1.85–1.90 (m, 2H, CH_2_), 2,41 (s, 3H, CH_3_), 3.05–3.09 (m, 2H, NCH_2_), 4.28–4.29 (m, 2H, NCH_2_), 6,88 (s, 1H, H-7), 6,95 (d, 1H, H-9, *J* = 8.4 Hz), 7,03 (d, 1H, H-10, *J* = 8.4 Hz), 7.26–7.28 (d, 2H, H_ph_), 7.34 (d, 1H, H-2), 7.54–7.58 (m, 2H, H-1, H-3), 7.55 (s, 1H, H-12), and 8.01 (d, 1H, H-4, *J* = 8.4 Hz). ^13^C NMR (75 MHz, CDCl_3_) δ: 21.54, 23.35, 26.85, 42.62, 45.81, 116.67, 119.41, 123.39, 125.26, 125.73, 126.32, 127.10, 127.64, 129.62, 129.69, 130.23, 133.28, 133.81, 137.00, 142.04, 143.30, and 151.59. HR MS (ESI) calcd for C_26_H_25_ClN_3_O_2_S_2_ [M+H]^+^: 510.1077, found: 510.1076. R_f_ = 0.29 (Al_2_O_3_, CH_2_Cl_2_).

- Synthesis of 8-chloroquinobenzothiazines with triazole substituents **27**–**33**.

To a solution of 8-chloro-6-propynyquinobenzothiazine **26** (0.16 g, 0.5 mmol) and copper iodide (I) (0.06 g) in dry toluene (5 mL), a corresponding organic azide (0.510 mmol) was added. The reaction mixture was stirred and heated at 70 °C for 48 h. Then, the solvent mixture was distilled under reduced pressure. The dry residue was dissolved in CH_2_Cl_2_ and purified by column chromatography (aluminum oxide 90 active neutral, Merck 1.01077.2000, CH_2_Cl_2_ as eluent) to give pure triazole derivatives **27**–**33**.

8-Chloro-6-((1-phenyl-1H-1,2,3-triazol-4-yl)methyl)-quino[3,2-b]benzo[1,4]thiazine (**27**):

Yield: 73% M.p.: 180–181 °C. ^1^H NMR (CDCl_3_) δ: 5.47 (s, 2H, CH_2_), 6.80 (d, 1H, H-7, *J* = 1.8 Hz), 6.88 (d, 1H, H-9, *J* = 7.8 Hz), 7.25 (d, 1H, H-10, *J* = 7.2 Hz), 7.32 (d, 1H, H-Ph, *J* = 7.8 Hz), 7.38–7.41 (m, 3H, 2H-Ph, CH), 7.46–7.48 (m, 2H, H-1, H-3), 7.58–7.61 (m, 3H, H-2, 2H-Ph), 7.67 (d, 1H, H-4, *J* = 7.8 Hz), and 8.11 (s, 1H, H-12). ^13^C NMR (75 MHz, CDCl_3_) δ: 42.85, 116.74, 117.99, 118.07, 120.57, 122.44, 122.89, 124.78, 126.25, 126.33, 127.02, 127.14, 128.68, 129.56, 129.68, 131.99, 133.82, 137.08, 142.35, 145.35, and 152.25. HR MS (ESI) calcd for C_24_H_17_ClN_5_S [M+H]^+^: 442.0893, found 442.0892. R_f_ = 0.60 (Al_2_O_3_, CH_2_Cl_2_).

8-Chloro-6-((1-(4-(trifluoromethyl)phenyl)-1*H*-1,2,3-triazol-4-yl)methyl)-quino[3,2-b]benzo[1,4]thiazine (**28**):

Yield: 77% M.p.: 188–189 °C. ^1^H NMR (CDCl_3_) δ: 5.57 (s, 2H, CH_2_), 6.90 (d, 1H, H-7, J = 5.4 Hz), 6.98 (d, 1H, H-9, *J* = 7.8 Hz), 7.35 (t, 1H, H-2, *J* = 7.6 Hz), 7.45 (s, 1H, H-10), 7.57 (m, 2H, H-1, H-3), 7.59 (s, 1H, CH), 7.58–7.77 (m, 3H, H-4, 2H-Ph), 7.86 (d, 2H, 2H-Ph, *J* = 8.4 Hz), and 8.32 (s, 1H, H-12). ^13^C NMR (75 MHz, CDCl_3_) δ: 42.69, 53.46, 116.65, 118.03, 118.15, 120.48, 123.00, 124.44, 124.91, 126.26, 126.44, 127.00, 127.03, 127.06, 127.08, 127.10, 129.67, 132.12, 133.83, 142.24, 145.22, and 151.18. HR MS (ESI) calcd for C_25_H_16_ClF_3_N_5_S [M+H]^+^: 510.0767, found 510.0772. R_f_ = 0.80 (Al_2_O_3_, CH_2_Cl_2_).

8-Chloro-6-[(1-benzyl-1*H*-1,2,3-triazol-4-yl)-methyl]-quinobenzo[1,4]thiazine (**29**):

Yield: 82% M.p.: 183–184 °C. ^1^H NMR (CDCl_3_) δ: 5.32 (s, 2H, CH_2_), 5.50 (s, 2H, CH_2_), 6.91 (d, 1H, H-7, *J* = 6.6 Hz), 6.98 (s, 1H, H-9, *J* = 8.4 Hz), 7.19–7.28 (m, 2H, H-2, 1H-Ph), 7.32–7.41 (m, 4H, 4H-Ph), 7.40 (d, 1H, H-10), 7.52 (H, 1H, H-3), 7.57 (d, 1H, H-1), 7.66 (d, 1H, H-4), 7.67 (s, 1H, H-12), and 7.72 (s, 1H, CH). ^13^C NMR (75 MHz, CDCl_3_) δ: 43.40, 54.10, 117.13, 118.52, 123.22, 123.22, 124.48, 125.00, 125.99, 126.34, 126.42, 127.10, 127.81, 127.88, 128.10, 128.59, 128.83, 129.04, 129.11, 129.14, 129.75, 132.56, 133.88, 134.72, 142.30, and 151.22. HR MS (ESI) calcd for: C_25_H_19_ClN_5_S [M+H]^+^: 456.1050, found: 456.1052. R_f_ = 0.63 (Al_2_O_3_, CH_2_Cl_2_).

8-chloro-6-((1-(4-fluorobenzyl)-1*H*-1,2,3-triazol-4-yl)methyl)-quino[3,2-b]benzo[1,4]thiazine (**30**):

Yield: 83% M.p.: 164–165 °C. ^1^H NMR (CDCl_3_) δ: 5.46 (s, 2H, 2CH_2_), 6.05 (s, 2H, CH_2_), 6.91–6.94 (m, 2H, H-7, H-9), 7.11–7.15 (m, 4H, H-3, H-10, 2H-Ph), 7.31 (d, 1H, H-1), 7.50 (t, 1H, H-2, *J* = 7.6 Hz), 7.64–7.67 (m, 2H, 2H-Ph), 7.94 (s, 1H, H-12), 8.40 (s, 1H, CH), and 8.57 (s, 1H, H-4). ^13^C NMR (75 MHz, CDCl_3_) δ: 46.48, 53.47, 115.97, 116.11, 119.31, 120.57, 121.64, 122.25, 124.80, 125.51, 126.05, 126.72, 127.29, 127.90, 129.57, 129.63, 130.37, 130.39, 132.35, 134.76, 136.72, 141.28, and 150.28. HR MS (ESI) calcd for: C_25_H_18_ClFN_5_S [M+H]^+^: 474.0955, found: 474.0944. R_f_ = 0.68 (Al_2_O_3_, CH_2_Cl_2_).

8-Chloro-6-[(1-(4-chlorobenzyl)-1*H*-1,2,3-triazol-4-yl)methyl]-quinobenzo[1,4]thiazine (**31**):

Yield: 83% M.p.: 184–185 °C. ^1^H NMR (CDCl_3_) δ: 5.47 (s, 2H, CH_2_), 5.50 (s, 2H, CH_2_), 6.91 (d, 1H, H-7, J = 6.0 Hz), 6.98 (d, 1H, H-9, *J* = 7.8 Hz), 7.13–7.14 (d, 2H, 2H-Ph), 7.28–7.30 (m, 2H, 2H-Ph), 7.34 (t, 1H, H-2, *J* = 6.6 Hz), 7.36 (d, 1H, H-10), 7.53–7.56 (m, 2H, H-1, H-3), 7.63 (d, 1H, H-4, *J* = 8.4 Hz), and 7.66 (s, 1H, H-12), 7.70 (s, 1H, CH). ^13^C NMR (75 MHz, CDCl_3_) δ: 43.25, 53.34, 116.98, 118.40, 118.46, 123.14, 124.41, 124.99, 126.03, 126.37, 126.49, 127.10, 129.18, 129.25, 129.71, 132.44, 133.22, 133.83, 134.64, 142.30, 144.47, 144.79, and 151.20. HR MS (ESI) calcd for: C_25_H_18_Cl_2_N_5_S [M+H]^+^: 490,0668, found: 490,0668. R_f_ = 0.58 (Al_2_O_3_, CH_2_Cl_2_).

4-((4-((8-Chloroquino[3,2-b]benzo[1,4]thiazin-6-yl)methyl)-1*H*-1,2,3-triazol-1-yl)methyl)benzonitrile (**32**):

Yield: 75% M.p.: 192–193 °C. ^1^H NMR (CDCl_3_) δ: 5.57 (s, 2H, CH_2_), 5.76 (s, 2H, CH_2_), 7.02 (d, 1H, H-7, *J* = 1.6 Hz), 7.06 (d, 1H, H-9, *J* = 6.6 Hz), 7.25 (d, 2H, 2H-Ph, *J* = 8.4 Hz), 7.28 (d, 1H, H-2, *J* = 7.8 Hz), 7.32–7.34 (m, 1H, H-10), 7.43 (d, 1H, H-3, *J* = 7.2 Hz), 7.56 (d, 2H, 2H-Ph, *J* = 8.4 Hz), 7.61–7.63 (m, 2H, H-4, H-12), and 7.80 (s, 1H, CH). ^13^C NMR (75 MHz, CDCl_3_) δ: 46.92, 53.41, 112.65, 118.10, 119.64, 120.87, 124.62, 126.15, 126.57, 126.88, 127.79, 128.03, 128.06, 128.13, 128.32, 132.75, 132.78, 132.81, 134.96, 137.44, 139.71, 141.14, and 150.25. HR MS (ESI) calcd for: C_26_H_18_ClN_6_S [M+H]^+^: 481.1002, found: 481.1011. R_f_ = 0.63 (Al_2_O_3_, CH_2_Cl_2_).

8-Chloro-6-((1-((phenylthio)methyl)-1*H*-1,2,3-triazol-4-yl)methyl)-quino[3,2-b]benzo[1,4]thiazine (**33**):

Yield: 80% M.p.: 141–142 °C. ^1^H NMR (CDCl_3_) δ: 5.44 (s, 2H, CH_2_), 5.55 (s, 2H, CH_2_), 6.90 (d, 1H, H-7, *J* = 1.6 Hz), 6.98 (d, 1H, H-9, *J* = 8.4 Hz), 7.16 (d, 2H, 2H-Ph), 7.18 (d, 1H, H-2, *J* = 7.8 Hz), 7.21 (d, 2H, 2H-Ph), 7.34–7.36 (m, 2H, H-10, H-Ph), 7.54 (t, 1H, H-3, J = 7.2 Hz), 7.57 (d, 1H, H-1, *J* = 7.8 Hz), 7.62 (d, 1H, H-4, *J* = 8.4 Hz), 7.63 (s, 1H, H-12), and 7.67 (s,1H, CH). ^13^C NMR (75 MHz, CDCl_3_) δ: 42.83, 54.16, 116.61, 117.87, 118.12, 122.12, 123.76, 124.75, 126.20, 126.29, 127.01, 127.26, 128.81, 129.34, 129.43, 129.48, 131.50, 131.94, 132.93, 133.75, 142.32, 145.10, 145.24, and 151.13. HR MS (ESI) calcd for: C_25_H_19_ClN_5_S_2_ [M+H]^+^: 488.0770, found: 488.0770. R_f_ = 0.78 (Al_2_O_3_, CH_2_Cl_2_).

### 3.2. Biological Assays

#### 3.2.1. Cell Line and Culture

The human cell lines, such as A549 (lung cancer), MDA-MB-231 (breast cancer), MiaPaca-2 (pancreatic cancer), PC3 (metastatic prostate cancer), HCT-116 (colon carcinoma), and HaCaT (immortalized keratinocytes), were sourced from the American Type Culture Collection (ATCC) in Rockville, USA. Culturing conditions varied as follows: HCT116 cells were cultured in MEM (ThermoSci, Waltham, MA, USA), A549, MDA-MB-231, MiaPaca, HaCaT cells in DMEM High Glucose and PC3 cells were cultured in RPMI (Biowest SAS, Nuaillé, France). The growth medium consisted of 10% fetal bovine serum (FBS, Sigma-Aldrich, St. Louis, MO, USA), 20 mM HEPES (Biowest, Nuaillé, France), and antibiotics (100 U/mL of penicillin and 100 μg/mL of streptomycin) from Gibco, Grand Island, NY, USA. All cells were maintained in a humidified incubator at 37 °C with a 5% CO_2_ atmosphere until they reached 80–90% confluence.

#### 3.2.2. MTT Assay

To evaluate the cytotoxic effects of the newly synthesized compounds, a preliminary MTT (3-(4,5-dimethylthiadiazol-2-yl)-2,5-diphenyl-2*H*-tetrazolium bromide) test was performed on two different cancer cell lines, A549 and MDA-MB231, as well as on one healthy HaCaT cell line. After obtaining the results, additional MTT tests were performed on three additional cancer cell lines (MiaPaca-2, PC3, and HCT-116), only on a selected group of compounds showing cytotoxic activity (**5**, **8**, **21**, **23**, and **25**). The study derivatives were subjected to testing at various concentrations (ranging from 5 to 140 µM), alongside the reference drug doxorubicin. These compounds were added to 96-well plates containing study cells (1 × 10^4^ cells per well) and incubated for 72 h. MTT analysis, as previously described [65], was employed.

Cell absorbance results were incorporated into the formula for calculating the relative MTT level (%), enabling the assessment of cell viability following exposure to the test compounds. The cell viability percentage represents the MTT reduction in cells treated with the test compounds compared to the control sample, where only the medium was added to the cells. The IC_50_ values, representing the concentration at which 50% of cell viability is inhibited, were calculated using Prism 8.0.1, GraphPad software.

#### 3.2.3. Apoptosis and Cell Cycle Analysis by Flow Cytometry (FCM)

To analyze the number of cells in early apoptosis, late apoptosis, or necrosis, A549, MiaPaca-2, and HCT-116 cell lines were cultured in 6-well plates with a seeding density of 1 × 10^5^ cells per well. These cells were then treated with selected compounds (**4**, **5**, and **8**) at their respective IC_50_ concentrations and incubated for 72 h. Subsequently, a commercially available kit, the FITC: Annexin V Apoptosis Detection Kit I from BD Biosciences Pharmingen in San Jose, CA, USA, was utilized to assess apoptosis. After 72 h the cells were harvested, washed, and labeled with Annexin V-FITC and propidium iodide (PI) following the manufacturer’s protocol (Becton Dickinson), as previously described [65]. The stained cells were analyzed by flow cytometry. The cells were identified as early apoptotic (Annexin V+/PI−) or late apoptotic/necrotic (Annexin V+/PI+).

- Cell cycle analysis:

In brief, cells were seeded at a density of 1 × 10^5^ cells/well on a six-well plate and allowed to adhere for 24 h. Subsequently, they were treated with selected compounds at their IC_50_ concentrations for an additional 24 h. Both population cells (the attached and detached) were collected and centrifuged at 400× *g* for 5 min at 4 °C. After centrifugation, the cells were washed twice with 0.9% NaCl and subsequently fixed in 500 μL of 70 % cold ethanol overnight.

Before analysis, the fixed cells underwent another centrifugation step at 850× *g* for 5 min at 4 °C and were washed with PBS. Following the washes, the cells were incubated with 50 μL of RNase (100 μg/mL) and 200 μL of propidium iodide (PI) (50 μg/mL) at 37 °C for 30 min in the dark. Finally, 100 μL of PBS was added to each sample. Flow cytometry (Becton Dickinson FACS Verse, Franklin Lakes, NJ, USA) was then used to analyze the cell cycle distribution, identifying cells in various stages including sub-G1, G0/G1, S, and G2/M phases. Each assay was performed in quadruplicate.

#### 3.2.4. In Vitro Antibacterial Studies

To evaluate the antibacterial effectiveness of chloroquinobenzothiazine derivatives, various bacterial strains from international microbe collections such as the American Type Culture Collection (ATCC) and the National Collection of Type Culture (NCTC) were examined, alongside a panel of clinical rods. This included two Gram-negative organisms, *E. coli ATCC 25922* and *P. aeruginosa ATCC 15442*, and a series of six Gram-positive strains: *S. aureus NCTC 4163*; *S. aureus ATCC 29213*, *25923*, and *6538*; and S*. epidermidis ATCC 12228* and *35984*.

The antimicrobial activity was examined using the Minimal Inhibitory Concentration (MIC) method, following standard CLSI procedures with slight adjustments. The MIC was determined using the two-fold serial broth microdilution method in 96-well microtitration plates containing Mueller-Hinton II broth medium (Becton Dickinson, Franklin Lakes, NJ, USA). The final inoculum for all tested bacteria was adjusted to 10^−6^ CFU/mL (colony-forming units per milliliter). The stock solution of the tested compounds was prepared in dimethyl sulfoxide (DMSO) and diluted to a maximum of 1% solvent content with a sterile medium. The MIC value recorded represents the lowest concentration of the tested antimicrobial agents (expressed in µg/mL) that inhibits visible growth of the microorganism after 19 h of incubation at 35 °C.

Additional details regarding the conducted biological studies, including cell culture, appropriate conditions, and methodology, were provided in our previous publication [66].

#### 3.2.5. Statistical Analysis

The statistical analysis was performed using GraphPad Prism 9 software (Graph Pad Software, San Diego, CA, USA). The results were reported as mean ± SD from a minimum of three independent experiments. Statistical significance of differences between values was assessed using analysis of variance with Dunnett’s multiple comparison post hoc test, and significance was defined as *p* < 0.05.

## 4. Conclusions

We report here the efficient synthesis of new derivatives of 6*H*-8-chloroquinobenzothiazines in the reactions of 2-amino-4-chlorobenzenothiol and 3-bromo-2-chloroquinoline. On the basis of spectroscopic studies, it was found that the thiazine ring formation reaction involves the Smiles rearrangement. The tetracyclic quinobenzothiazine ring system was identified using advanced two-dimensional ^1^H and ^13^C NMR spectra (COSY, ROESY, HSQC, and HMBC) of N-methyl derivatives. The efficient reaction to obtain 6*H*-8-chloroquinobenzothiazine allowed it to be used for the synthesis of a series of new N-substituted quinobenzothiases modified with the quinolyl ring, which are new analogues of chlorpromazine. 6*H*-8-chloroquinobenzothiazine was further transformed into N-dialkylaminoalkyl, N-acylaminoalkyl, N-sulfonylaminoalkyl, and N-methyltriazolyl derivatives.

All derivatives were tested against two human carcinoma cell lines (A549, MDA-MB-231) and a normal cell line HaCaT to determine their IC_50_ values using the MTT method. Of the twenty-four compounds tested, nine compounds (**5**, **8**, **9**, **11**, **20**, **21**, **23**, **25**, and **27**) showed promising activity against A549 cells without affecting HaCaT cells. Compounds **8**, **20**, **21**, and **25** showed the most promising cytotoxicity towards A549 cells and a higher selectivity index (SI = 7.6–10.7) than the reference compound—doxorubicin (SI = 0.14–0.15). Based on these results, these most active new chlorpromazine analogues were further evaluated in three additional cancer cell lines (MiaPaca-2, PC3, and HCT-116) using the MTT method. Apart from the A549 cell line, HCT 116 cells showed the highest sensitivity to the tested substances, while PC3 cells showed lower sensitivity. Compounds **8**—with dimethylaminopropyl substituent—and **23**—with methanesulfonylaminobutyl substituent—displayed the highest selectivity index (143), with IC_50_ values of 1.6 µM and 0.7 µM, respectively, against HCT-116 cells, while showing no cytotoxic effects on HaCaT cells. Compound **11**—with 1-methyl-2-piperidylethyl substituent—exhibited notably higher cytotoxicity specifically against HCT-116 cells (7.7 µM).

Studies on the mechanisms of cytotoxic action have also been carried out. These studies confirmed the pro-apoptotic activity of the tested chlorpromazine analogues, especially in terms of inducing late apoptosis or necrosis in the A549, MiaPaCa-2, and HCT-116 cancer cell lines.

The effects of the new derivatives on the cell cycle were also investigated to elucidate the inhibition mechanisms. Compounds **5**, **8**, **20**, **21**, and **25** were found to induce G0/G1 phase arrest in cell lines (A549 and MiaPaca-2, respectively). A simultaneous reduction in the number of cells in the S and G2/M phases was also observed. The results clearly suggest that the tested derivatives exert antiproliferative effects by inducing cell cycle arrest in the S phase and, consequently, apoptosis.

New chlorpromazine analogues were also tested against selected standard Gram-positive bacteria (various strains of *S. aureus* and *S. epidermidis*) as well as Gram-negative bacteria (*E. coli* and *P. aeruginosa*). Seven of the tested 8-chloroquinobenzothiazines (**5**–**9**, **20**, **21**) showed moderate antibacterial activity, mainly against standard strains of staphylococci. The most prominent activity against standard strains was observed for compound **21** (MIC = 2–8 µg/mL).

The obtained test results indicate five new chlorpromazine analogues as very promising compounds, as follows: **8** (6-(3-dimethylaminopropyl)-8-chloroquinobenzothiazine), **11** (6-(1-methyl-2-piperidylethyl)-8-chloroquinobenzothiazine), **20** (6-chloroethylureidopropyl-8-chloroquinobenzothiazine), **21** (6-chloroethylureidobutyl-8-chloroquinobenzothiazine), and **23** (8-chloro-6-methanesulfonyl-aminobutylquinobenzothiazine). Compounds **8**, **11**, **20**, and **21** have both antibacterial and cytotoxic properties, while derivative **23** shows high activity against the HCT116 cell line with no toxicity on HaCaT cells.

## Figures and Tables

**Table 1:** The proton–proton correlation of compound **6**.

^1^H NMR (ppm)	ROESY	COSY
3.61 CH_3_	6.94	
6.94 H7	3.61	
6.96 H9		7.05
7.05 H10		6.96
7.32 H2		7.55, 7.56
7.55 H3 7.56 H1		7.32
7.70 H12		
7.79 H4		7.55, 7.56

**Table 2:** The proton–carbon correlation of compound **6**.

^13^C NMR	HSQC	HMBC
29.73	3.61	
115.70	3.61 and 6.94 C7	6.96
118.19		6.96 and 6.94 C8
118.95		6.94 C4a
122.48	6.96 C9	
124.53	7.32 C2	
125.99		7.32 C11a
126.30	7.54–7.57 C1	
127.23	7.79 C4	
127.47	7.05 C10	
129.33	7.54–7.57 C3	7.54–7.57
132.21	7.70 C12	
133.60		6.94 and 7.05 C10a
144.17		7.05 and 3.61 C6a
145.74		7.70 and 7.54–7.57 C12a
152.77		7.70 and 3.61 C5a

**Table 3:** Cytotoxic activity (IC_50_, µM) of studied compounds estimated by the MTT assay ^a^.

Compound	Cancer Cells				Normal Cells
	A549 ^d^		MDA ^e^		HaCaT ^f^
	IC_50_^b^	SI ^c^	IC_50_	SI	IC_50_
**5**	14.9 ± 2.7	3.8	76.5 ± 0.8	0.7	56.5 ± 4.5
**7**	27.2 ± 8.4	3.7	>100	1.0	>100
**8**	8.2 ± 3.6	7.6	52.1 ± 2.7	1.2	62.3 ± 2.5
**9**	17.4 ± 4.1	5.7	77.1 ± 1.6	1.3	>100
**10**	63.8 ± 8.5	1.6	>100	1.0	>100
**11**	16.0 ± 4.2	1	16.7 ± 1.1	0.9	12.7 ± 1.9
**16**	24.6 ± 6.1	4.1	76.6 ± 8.9	1.3	>100
**17**	82.3 ± 6.2	1.2	>100	1.0	>100
**18**	86.6 ± 0.8	1.1	>100	1.0	>100
**19**	>100	1.0	>100	1.0	>100
**20**	9.3 ± 1.2	10.7	48.6 ± 7.3	2.0	>100
**21**	6.98 ± 1.2	0.16	7.4 ± 1.2	0.15	1.1 ± 0.3
**22**	>100	1.0	>100	1.0	>100
**23**	30.5 ± 8.3	3.3	>100	0.2	>100
**24**	>100	1.0	>100	1.0	>100
**25**	9.45 ± 1.3	10.5	>100	1.0	<100
**26**	>100	1.0	95.8 ± 9.2	0.7	71.7 ± 5.7
**27**	27.5 ± 6.2	2.5	>100	0.7	70.2 ± 10.4
**28**	>100	1.0	>100	1.0	>100
**29**	>100	1.0	>100	1.0	>100
**30**	>100	1.0	>100	1.0	>100
**31**	>100	1.0	>100	1.0	>100
**32**	>100	1.0	>100	1.0	>100
**33**	>100	1.0	>100	1.0	>100
DX ^g^	0.6 ± 0.2	0.14	0.8 ± 0.1	0.15	0.3 ± 0.1

^a^ Data are expressed as mean SD, ^b^ IC_50_ (µM)—the concentration of the compound that corresponds to a 50% growth inhibition of cell line (as compared to the control) after the cells are cultured for 72 h with the individual compound. ^c^ The SI (selectivity index) was calculated using the formula: SI = IC_50_ for normal cell line/IC_50_ cancer cell line. ^d^ Human lung cancer (A549). ^e^ Human breast cancer (MDA-MB231). ^f^ Human immortal keratinocyte cell line from adult human skin (HaCaT). ^g^ The selected reference compound commonly used in cancer treatment (doxorubicin).

**Table 4:** Cytotoxic activity (IC_50_, µM) of selected compounds estimated by the MTT assay ^a^.

Compound	Cancer Cells						Normal Cells
	MiaPaCa-2 ^d^		PC3 ^e^		HCT116 ^f^		HaCaT ^g^
	IC_50_^b^	SI ^c^	IC_50_	SI	IC_50_	SI	IC_50_
**5**	11.1 ± 0.4	5.0	76.5 ± 8.1	0.7	33.5 ± 6.8	1.7	56.5 ± 6.4
**8**	40.2 ± 0.7	1.6	52.1 ± 7.1	1.2	1.6 ± 0.8	39	62.3 ± 3.5
**9**	57.4 ± 9.6	1.7	77.1 ± 9.4	1.3	17.5 ± 1.4	5.7	>100
**11**	24.3 ± 3.5	0.5	16.7 ± 1.8	0.7	7.7 ± 1.2	1.6	12.7 ± 2.1
**20**	23.2 ± 2.7	4.3	34.8 ± 9.8	2.8	10.4 ± 1.6	8.8	>100
**21**	6.4 ± 2.4	0.2	76.6 ± 9.8	0.1	11.3 ± 2.2	0.1	1.1 ± 0.2
**23**	98.4 ± 5.6	1.0	>100	1.0	0.7 ± 0.08	143	>100
**25**	>100	1.0	48.6 ± 4.7	2.0	>100	1.0	>100
**27**	37.4 ± 5.6	1.9	>100	1.0	49.6 ± 4.7	1.4	71.7 ± 7.5
DX ^h^	0.6 ± 0.2	0.14	0.8 ± 0.1	0.15	0.59 ± 0.02	0.5	0.3 ± 0.1

^a^ Data are expressed as mean SD. ^b^ IC_50_ (µM)—the concentration of the compound that corresponds to a 50% growth inhibition of cell line (as compared to the control) after culturing the cells for 72 h with the individual compound. ^c^ The SI (selectivity index) was calculated using formula: SI = IC_50_ for normal cell line/IC_50_ cancer cell line. ^d^ Human pancreas cancer (MiaPaCa-2). ^e^ Human prostate cancer (PC3). ^f^ Human colon cancer (HCT-116). ^g^ Human immortal keratinocyte cell line from adult human skin (HaCaT). ^h^ The selected reference compound commonly used in cancer treatment (doxorubicin).

**Table 5:** Activity of compounds against Standard Bacterial Strains—expressed as minimal inhibitory concentrations [MICs (μg/mL)].

Compound	Bacterial Strains							
	*S. aureus NCTC 4163*	*S. aureus ATCC 25923*	*S. aureus ATCC 6538*	*S. aureus ATCC 29213*	*S. epidermidis ATCC 12228*	*S. epidermidis ATCC 35984*	*E. coli ATCC 25922*	*P. aeruginosa ATCC 15442*
**5**	8	8	8	8	8	8	256	256
**7**	8	8	8	8	8	8	>256	>256
**8**	8	8	8	8	8	8	64	256
**9**	16	16	16	16	16	16	>256	>256
**10**	>256	>256	>256	>256	>256	>256	>256	>256
**11**	8	128	128	128	256	256	>256	>256
**16**	>256	>256	>256	>256	>256	>256	>256	>256
**17**	>256	>256	>256	>256	>256	>256	>256	>256
**18**	>256	>256	>256	>256	>256	>256	>256	>256
**19**	>256	>256	>256	>256	>256	>256	>256	>256
**20**	8	8	8	8	8	4	>256	>256
**21**	2	2	2	2	2	2	8	>256
**22**	>256	>256	>256	>256	>256	>256	>256	>256
**23**	>256	>256	>256	>256	>256	>256	>256	>256
**24**	>256	>256	>256	>256	>256	>256	>256	>256
**25**	>256	>256	>256	>256	>256	>256	>256	>256
**26**	>256	>256	>256	>256	>256	>256	>256	>256
**27**	>256	>256	>256	>256	>256	>256	>256	>256
**28**	>256	>256	>256	>256	>256	>256	>256	>256
**29**	>256	>256	>256	>256	>256	>256	>256	>256
**30**	>256	>256	>256	>256	>256	>256	>256	>256
**31**	>256	>256	>256	>256	>256	>256	>256	>256
**32**	>256	>256	>256	>256	>256	>256	>256	>256
**33**	>256	>256	>256	>256	>256	>256	>256	>256
control—ciprofloxacin	0.125	0.25	0.125	0.25	0.125	0.125	0.0075	0.125

## Data Availability

All data generated or analyzed during this study are included in this published article; further inquiries can be directed to the authors.

## Supplementary Materials

The following supporting information can be downloaded at: https://www.mdpi.com/article/10.3390/ijms25084148/s1.
