# Supplementary material for: Novel Tetracyclic Azaphenothiazines with the Quinoline Ring as New Anticancer and Antibacterial Derivatives of Chlorpromazine
Source: Int J Mol Sci. 2024 Apr 9;25(8):4148. doi: 10.3390/ijms25084148 (PMC11050599; doi:10.3390/ijms25084148)
Supplement: Supplementary file 1 [file ijms-25-04148-s001.zip › ijms-2927679-supplementary.pdf]

Novel Tetracyclic Azaphenothiazines with the Quinoline Ring as New Anticancer and Antibacterial Derivatives of Chlorpromazine

Małgorzata Jeleń [1,*], Dagmara Otto-Ślusarczyk [2], Beata Morak-Młodawska [1] and Marta Struga [2]

[1] Department of Organic Chemistry, The Medical University of Silesia, Jagiellońska 4, 41-200 Sosnowiec, Poland; bmlodawska@sum.edu.pl
[2] Chair and Department of Biochemistry, Medical University of Warsaw, 02-097 Warsaw, Poland

Content:

1. NMR spectra and HR MS of 6*H*-8-chloroquino[3,2-b]benzo[1,4]thiazine (**5**). 2

2. NMR spectra and HR MS of 8-chloro-6-methylquino[3,2-b]benzo[1,4]thiazine (**6**). 4

3. NMR spectra and HR MS of 2-(8-chloroquino[3,2-b]benzo[1,4]thiazin-6-yl)-N,N-diethylethan-1-amine (**7**). 9

4. NMR spectra and HR MS of 3-(8-chloroquino[3,2-b]benzo[1,4]thiazin-6-yl)-N,N-dimethylpropan-1-amine (**8**). 11

5. NMR spectra and HR MS of 8-chloro-6-(2-(pyrrolidin-1-yl)ethyl)-quino[3,2-b]benzo[1,4]thiazine (**9**). 13

6. NMR spectra and HR MS of 8-chloro-6-(2-(piperidin-1-yl)ethyl)-quino[3,2-b]benzo[1,4]thiazine (**10**). 15

7. NMR spectra and HR MS of 8-chloro-6-(2-(1-methylpiperidin-2-yl)ethyl)-quino[3,2-b]benzo[1,4]thiazine (**11**). 17

8. NMR spectra and HR MS of 2-(3-(8-chloroquino[3,2-b]benzo[1,4]thiazin-6-yl)propyl)isoindoline-1,3-dione (**12**). 19

9. NMR spectra and HR MS of 3-(8-chloroquino[3,2-b]benzo[1,4]thiazin-6-yl)propan-1-amine (**14**). 21

10. NMR spectra and HR MS of N-(3-(8-chloroquino[3,2-b]benzo[1,4]thiazin-6-yl)propyl)acetamide (**16**). 22

11. NMR spectra and HR MS of ethyl(3-(8-chloro-quino[3,2-b]benzo[1,4]thiazin-6-yl)propyl)carbamate (**18**). 24

12. NMR spectra and HR MS of 1-(3-(8-chloroquino[3,2-b]benzo[1,4]thiazin-6-yl)propyl)-3-(2-chloroethyl)urea (**20**). 26

13. NMR spectra and HR MS of N-(3-(8-chloroquino[3,2-b]benzo[1,4]thiazin-6-yl)propyl)methanesulfonamide (**22**). 28

14. NMR spectra and HR MS of N-(3-(8-chloroquino[3,2-b]benzo[1,4]thiazin-6-yl)propyl)-4-methylbenzenesulfonamide (**24**). 30

15. NMR spectra and HR MS of 2-(4-(8-chloro quino[3,2-b]benzo[1,4]thiazin-6-yl)butyl)isoindoline-1,3-dione (**13**). 32

16. NMR spectra and HR MS of 4-(8-chloroquino[3,2-b]benzo[1,4]thiazin-6-yl)butan-1-amine (**15**). 34

17. NMR spectra and HR MS of N-(4-(8-chloroquino[3,2-b]benzo[1,4]thiazin-6-yl)butyl)acetamide (**17**). 36

18. NMR spectra and HR MS of ethyl(4-(8-chloro-quino[3,2-b]benzo[1,4]thiazin-6-yl)butyl)carbamate (**19**). 38

19. NMR spectra and HR MS of 1-(4-(8-chloroquino[3,2-b]benzo[1,4]thiazin-6-yl)butyl)-3-(2-chloroethyl)urea (**21**). 40

20. NMR spectra and HR MS of N-(4-(8-chloroquino[3,2-b]benzo[1,4]thiazin-6-yl)butyl)methanesulfonamide (**23**). 42

21. NMR spectra and HR MS of N-(4-(8-chloroquino[3,2-b]benzo[1,4]thiazin-6-yl)butyl)-4-methylbenzenesulfonamide (**25**). 44

22. NMR spectra and HR MS of 8-chloro-6-((1-phenyl-1*H*-1,2,3-triazol-4-yl)methyl)-quino[3,2-b]benzo[1,4]thiazine (**27**). 46

23. NMR spectra and HR MS 8-chloro-6-((1-(4-(trifluoromethyl)phenyl)-1*H*-1,2,3-triazol-4-yl)methyl)-quino[3,2-b]benzo[1,4]thiazine (**28**). 48

24. NMR spectra and HR MS of 6-((1-benzyl-1*H*-1,2,3-triazol-4-yl)methyl)-8-chloroquino[3,2-b]benzo[1,4]thiazine (**29**). 50

25. NMR spectra and HR MS of 8-chloro-6-((1-(4-fluorobenzyl)-1*H*-1,2,3-triazol-4-yl)methyl)-quino[3,2-b]benzo[1,4]thiazine (**30**). 52

26. NMR spectra and HR MS of 8-chloro-6-((1-(4-chlorobenzyl)-1*H*-1,2,3-triazol-4-yl)methyl)-quino[3,2-b]benzo[1,4]thiazine (**31**). 54

27. NMR spectra and HR MS of 4-((4-((8-chloroquino[3,2-b]benzo[1,4]thiazin-6-yl)methyl)-1*H*-1,2,3-triazol-1-yl)methyl)benzonitrile (**32**). 56

28. NMR spectra and HR MS of 8-chloro-6-((1-((phenylthio)methyl)-1*H*-1,2,3-triazol-4-yl)methyl)-quino[3,2-b]benzo[1,4]thiazine (**33**). 57

1. NMR spectra and HR MS of 6*H*-8-chloroquino[3,2-b]benzo[1,4]thiazine (**5**).

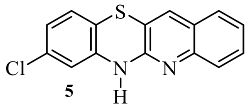

5

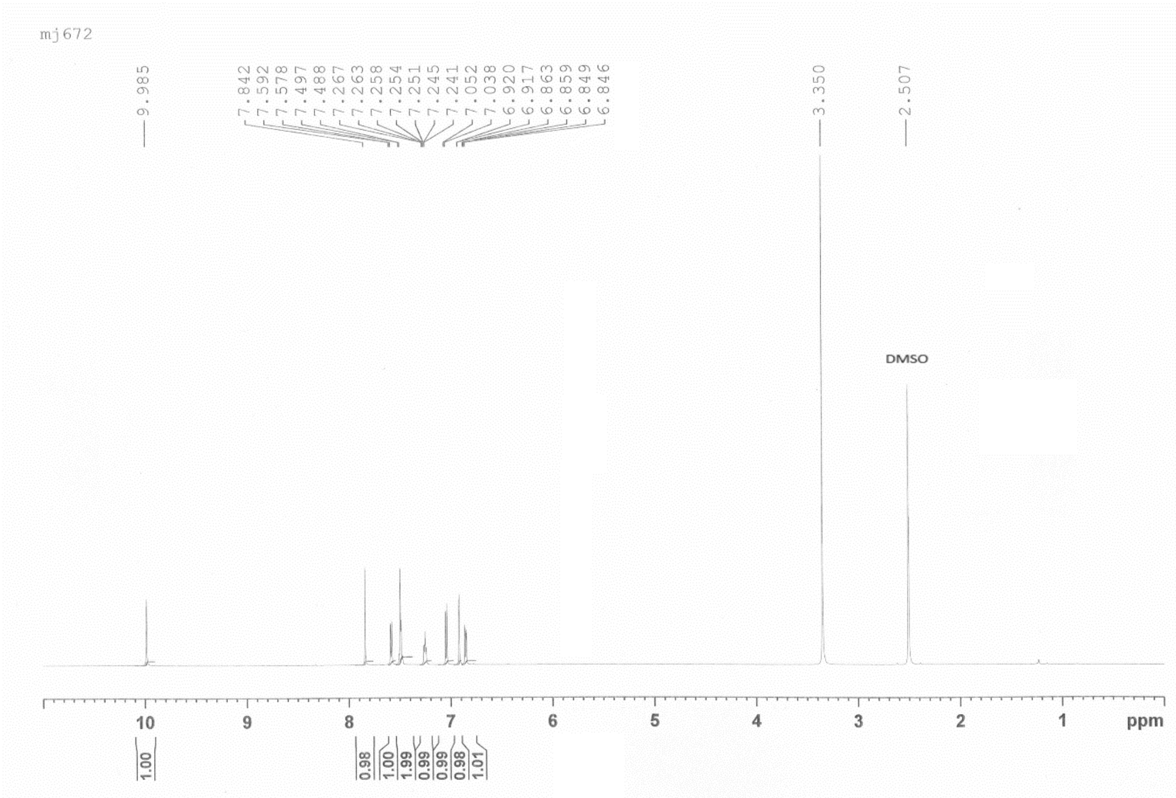
mj672
9.985
7.842
7.592
7.578
7.497
7.488
7.267
7.263
7.258
7.254
7.251
7.245
7.241
7.052
7.038
6.920
6.917
6.863
6.859
6.849
6.846
3.350
2.507
DMSO
10 9 8 7 6 5 4 3 2 1 ppm
1.00
0.98
1.00
1.99
0.99
0.99
0.98
1.01

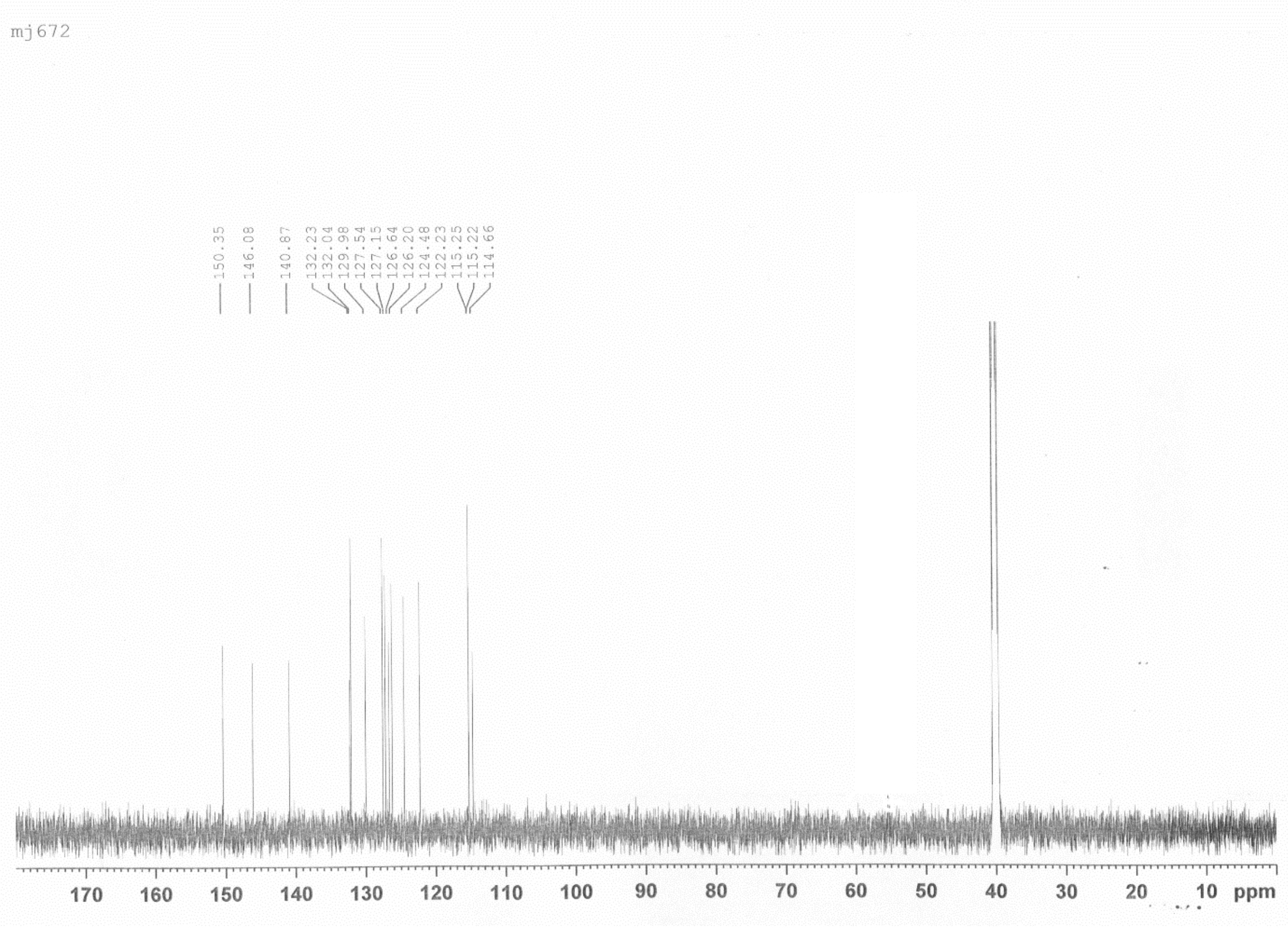
mj672
150.35
146.08
140.87
132.23
132.04
129.98
127.54
127.15
126.64
126.20
124.48
122.23
115.25
115.22
114.66
170 160 150 140 130 120 110 100 90 80 70 60 50 40 30 20 10 ppm

Acquisition Parameter

Source Type	ESI	Ion Polarity	Positive	Set Nebulizer	0.3 Bar
Focus	Active	Set Capillary	4000 V	Set Dry Heater	200 °C
Scan Begin	100 m/z	Set End Plate Offset	-500 V	Set Dry Gas	3.0 l/min
Scan End	1000 m/z	Set Charging Voltage	2000 V	Set Divert Valve	Source
		Set Corona	0 nA	Set APCI Heater	0 °C

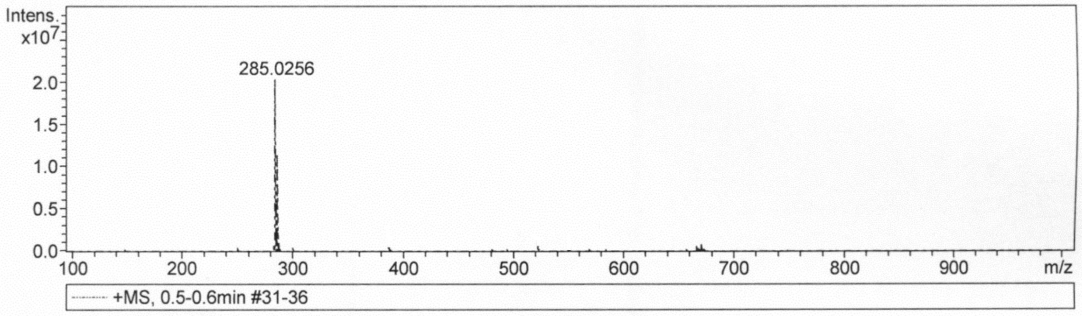

#	m/z	Res.	S/N	I	I %	FWHM
1	285.0256	17668	56320.7	20377480	100.0	0.0161
2	287.0218	37360	31654.5	11533646	56.6	0.0077

2. NMR spectra and HR MS of 8-chloro-6-methylquino[3,2-b]benzo[1,4]thiazine (**6**).

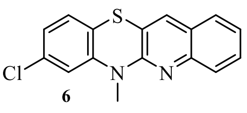

6

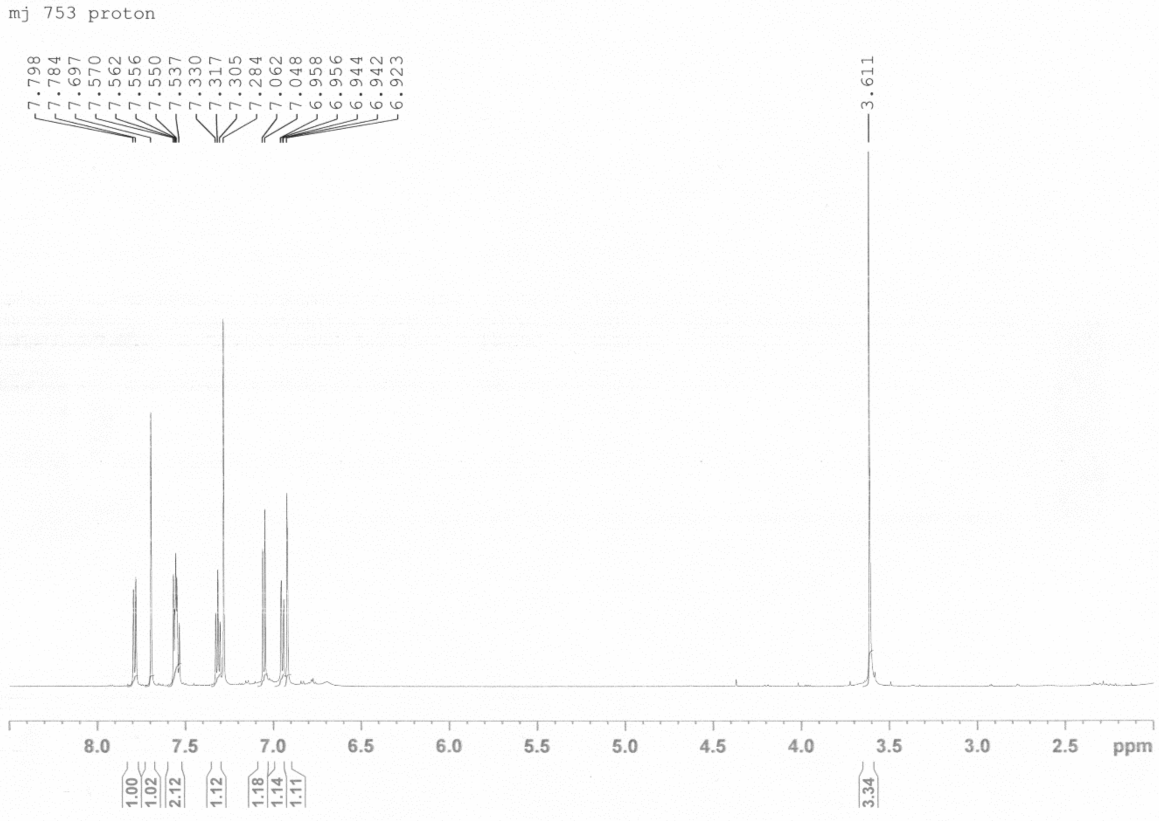

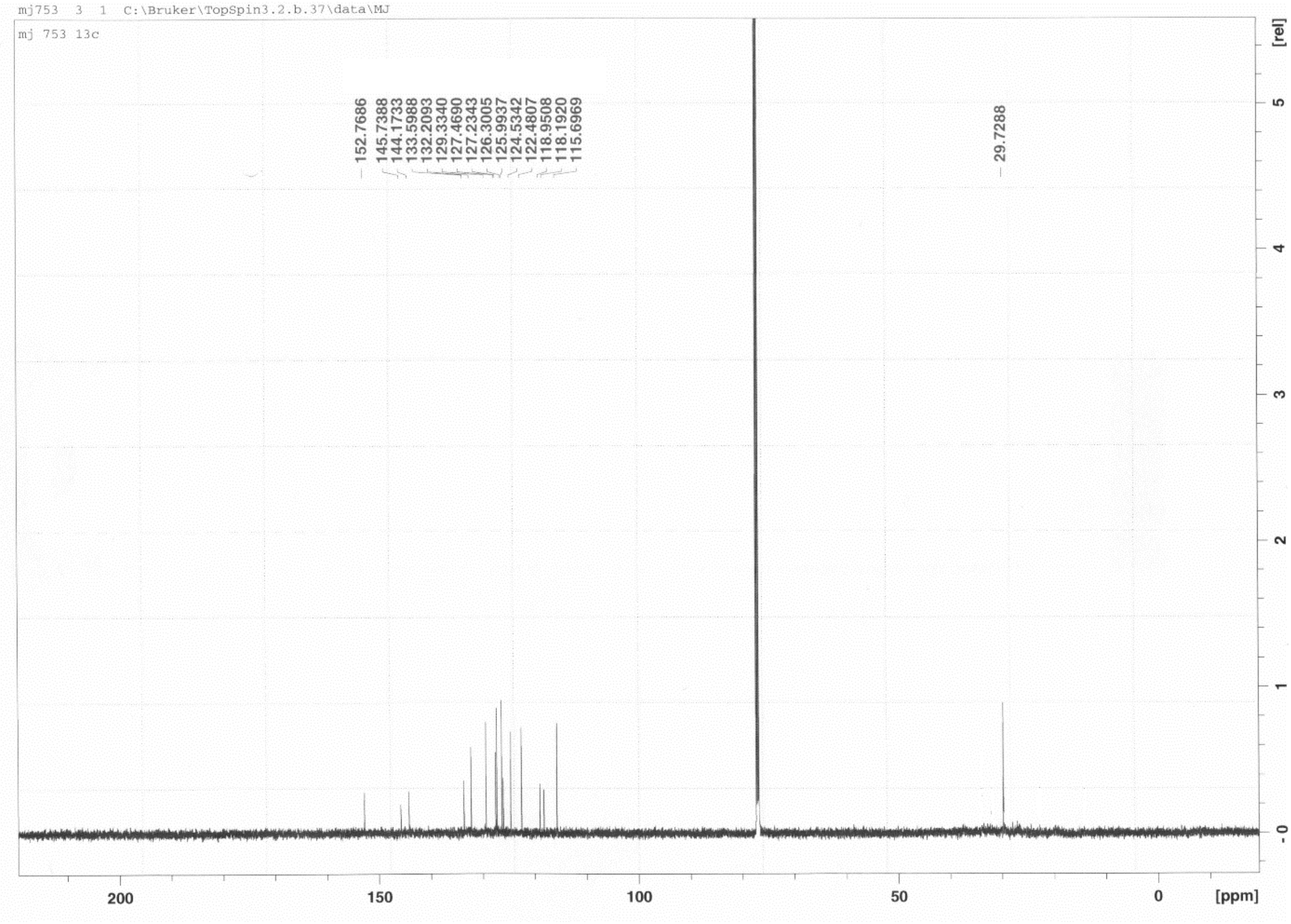

Acquisition Parameter

Source Type	ESI	Ion Polarity	Positive	Set Nebulizer	0.3 Bar
Focus	Active	Set Capillary	4000 V	Set Dry Heater	240 °C
Scan Begin	100 m/z	Set End Plate Offset	-500 V	Set Dry Gas	4.0 l/min
Scan End	1000 m/z	Set Charging Voltage	2000 V	Set Divert Valve	Source
		Set Corona	0 nA	Set APCI Heater	0 °C

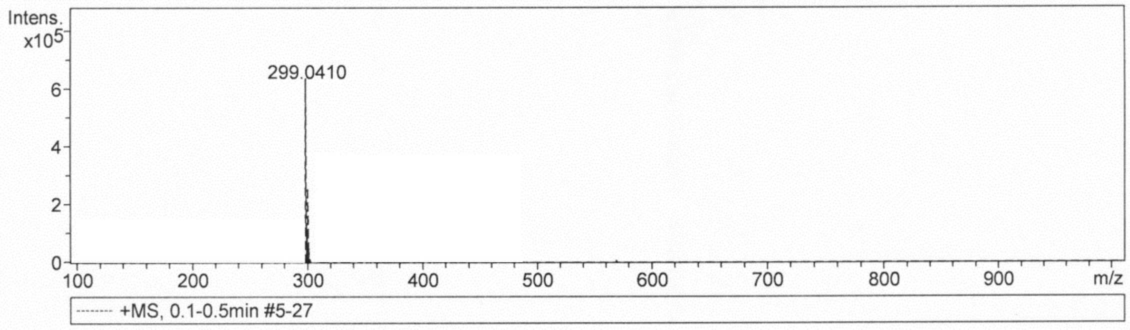

#	m/z	Res.	S/N	I	I %	FWHM
1	299.0410	22712	9224.3	617903	100.0	0.0132

mj 753 roesy

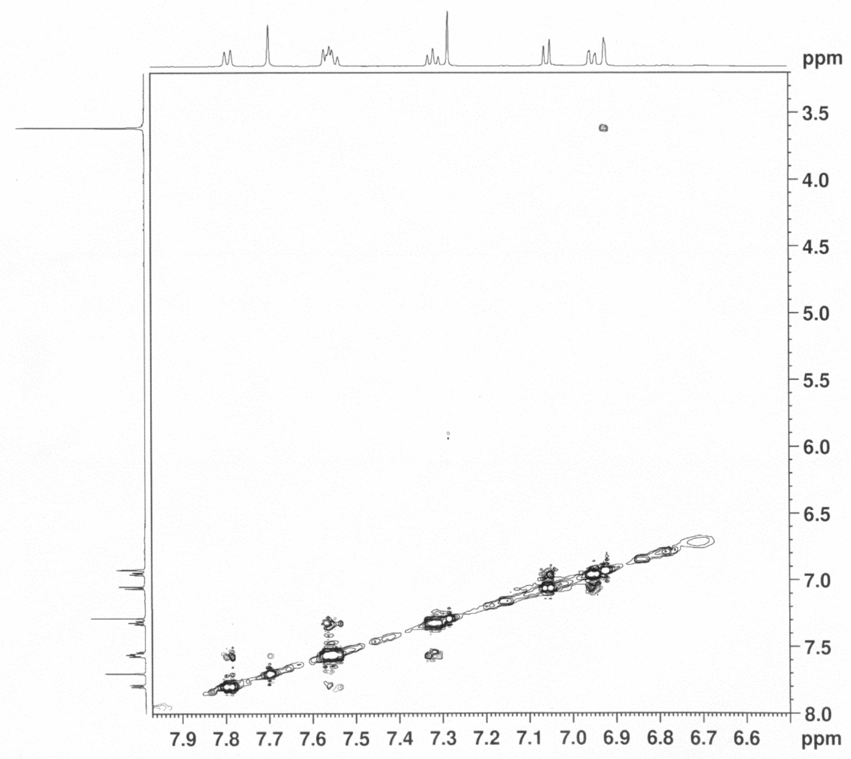

mj 753 cosy

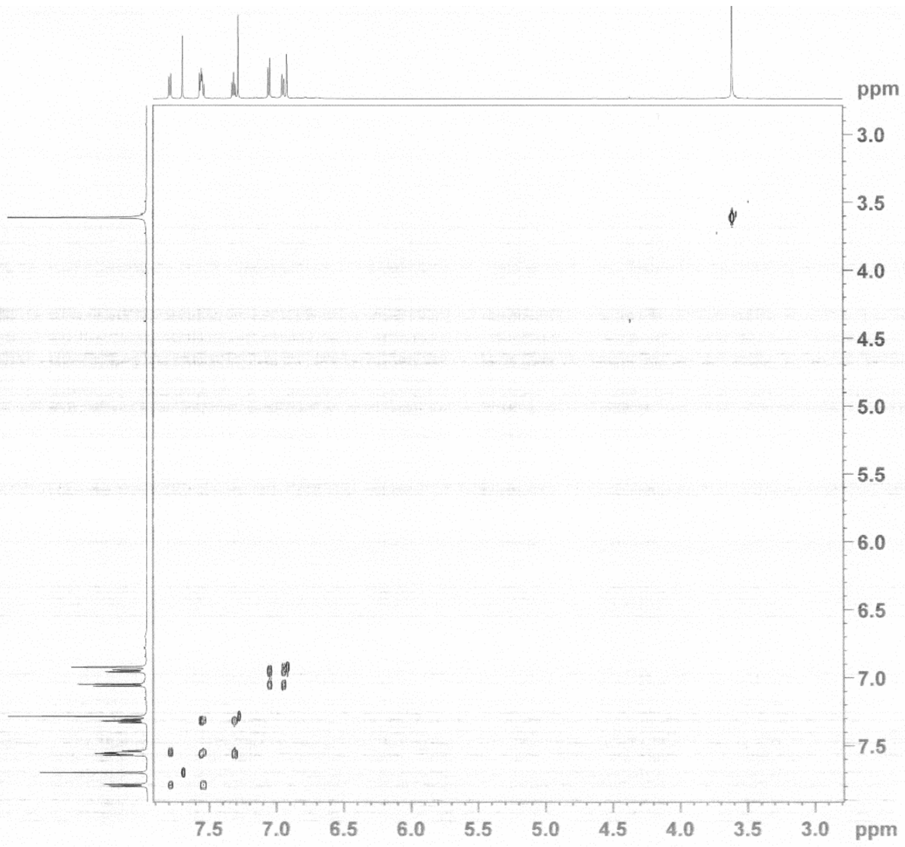

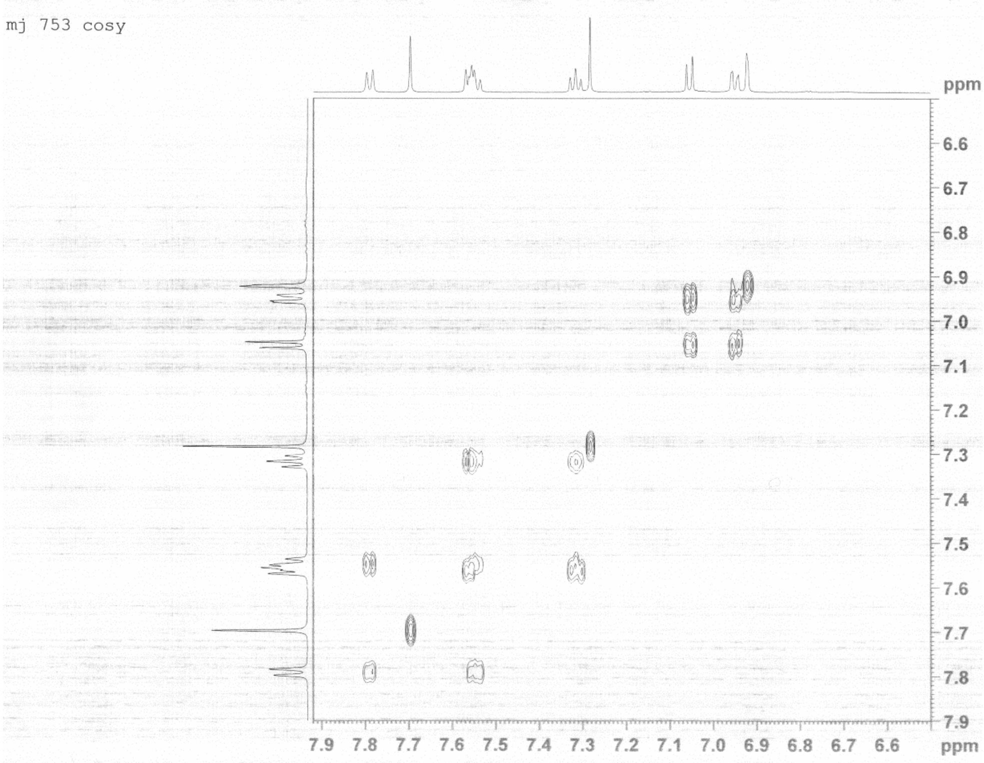

mj 753 cosy
ppm
6.6
6.7
6.8
6.9
7.0
7.1
7.2
7.3
7.4
7.5
7.6
7.7
7.8
7.9
7.9 7.8 7.7 7.6 7.5 7.4 7.3 7.2 7.1 7.0 6.9 6.8 6.7 6.6
ppm

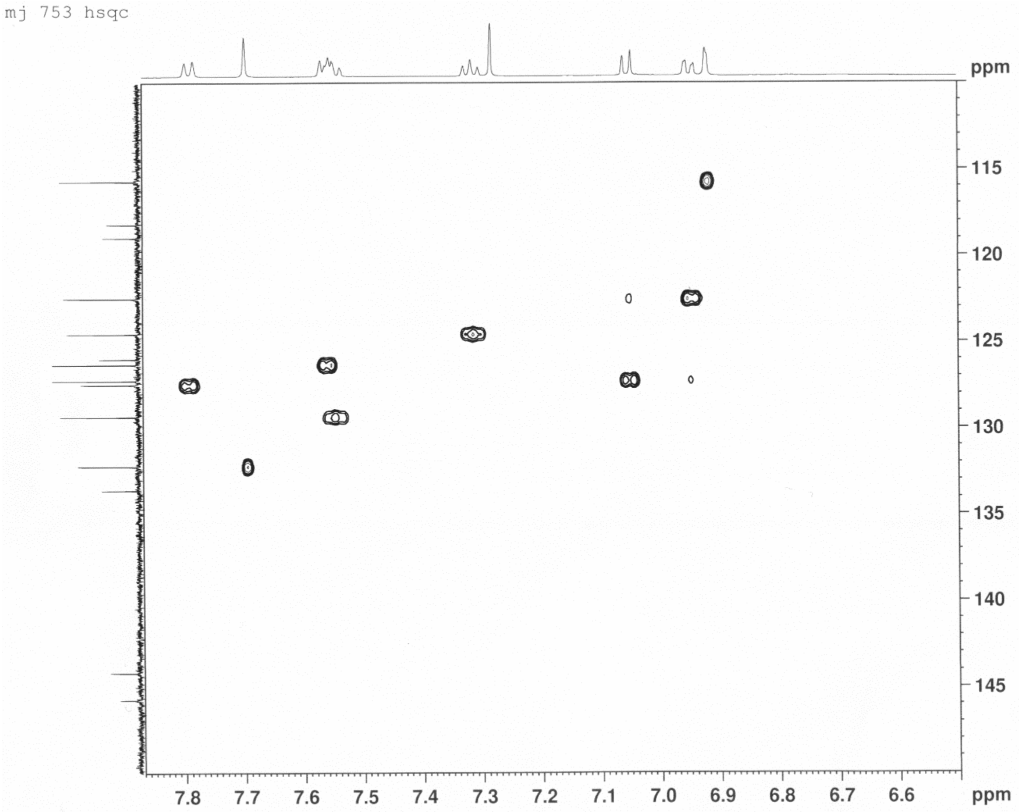

mj 753 hsqc
ppm
115
120
125
130
135
140
145
7.8 7.7 7.6 7.5 7.4 7.3 7.2 7.1 7.0 6.9 6.8 6.7 6.6
ppm

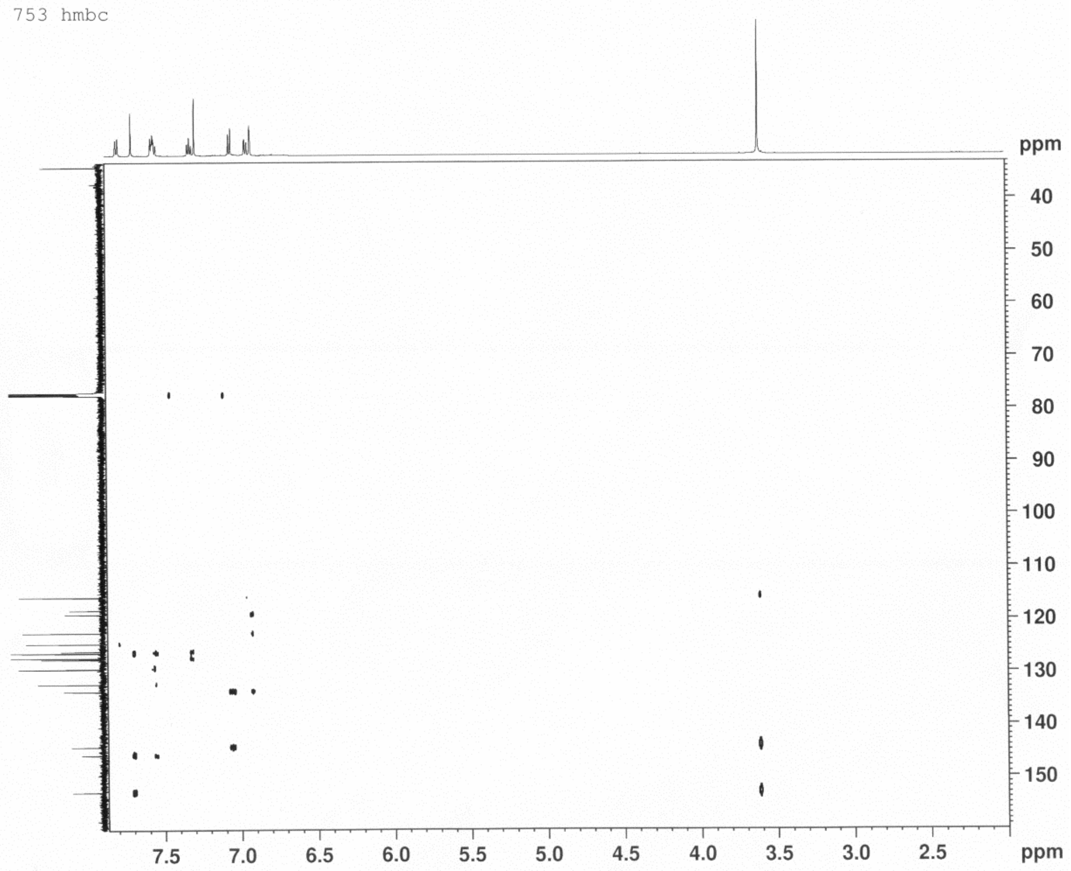
753 hmbc
ppm
40
50
60
70
80
90
100
110
120
130
140
150
7.5
7.0
6.5
6.0
5.5
5.0
4.5
4.0
3.5
3.0
2.5
ppm

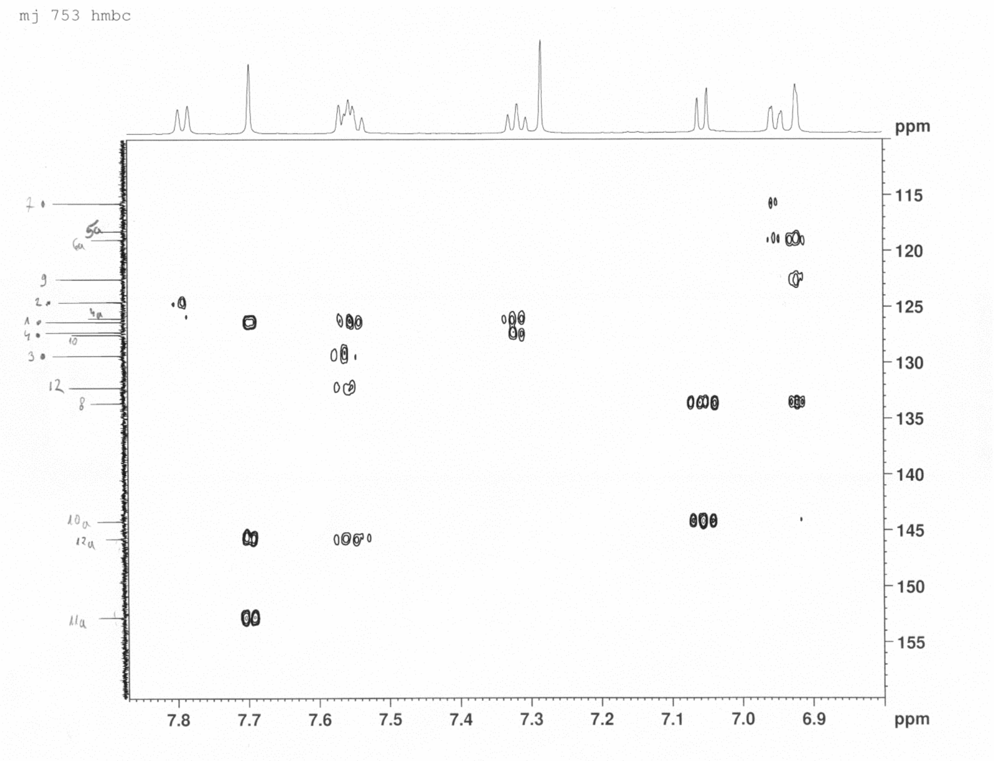
mj 753 hmbc
ppm
115
120
125
130
135
140
145
150
155
7.8
7.7
7.6
7.5
7.4
7.3
7.2
7.1
7.0
6.9
ppm

3. NMR spectra and HR MS of 2-(8-chloroquino[3,2-b]benzo[1,4]thiazin-6-yl)-N,N-diethylethan-1-amine (**7**)

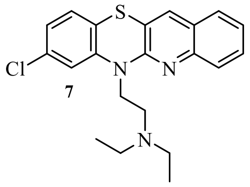

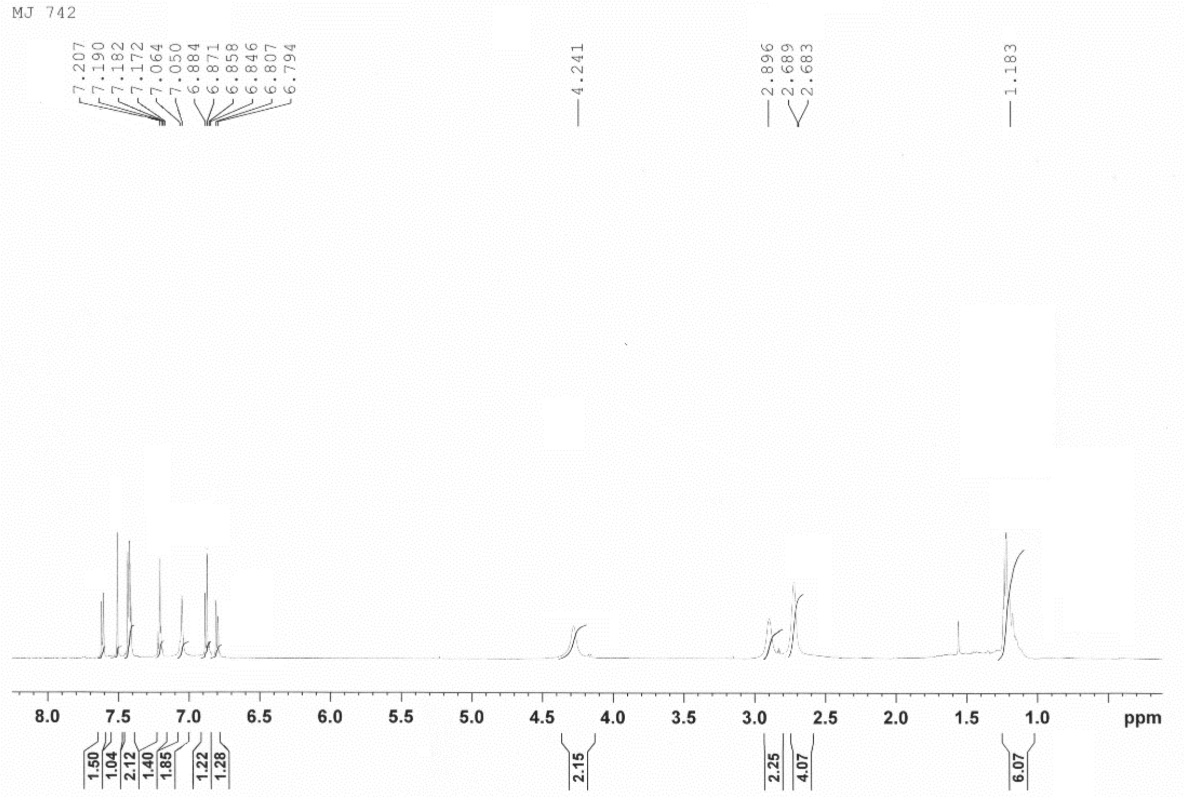

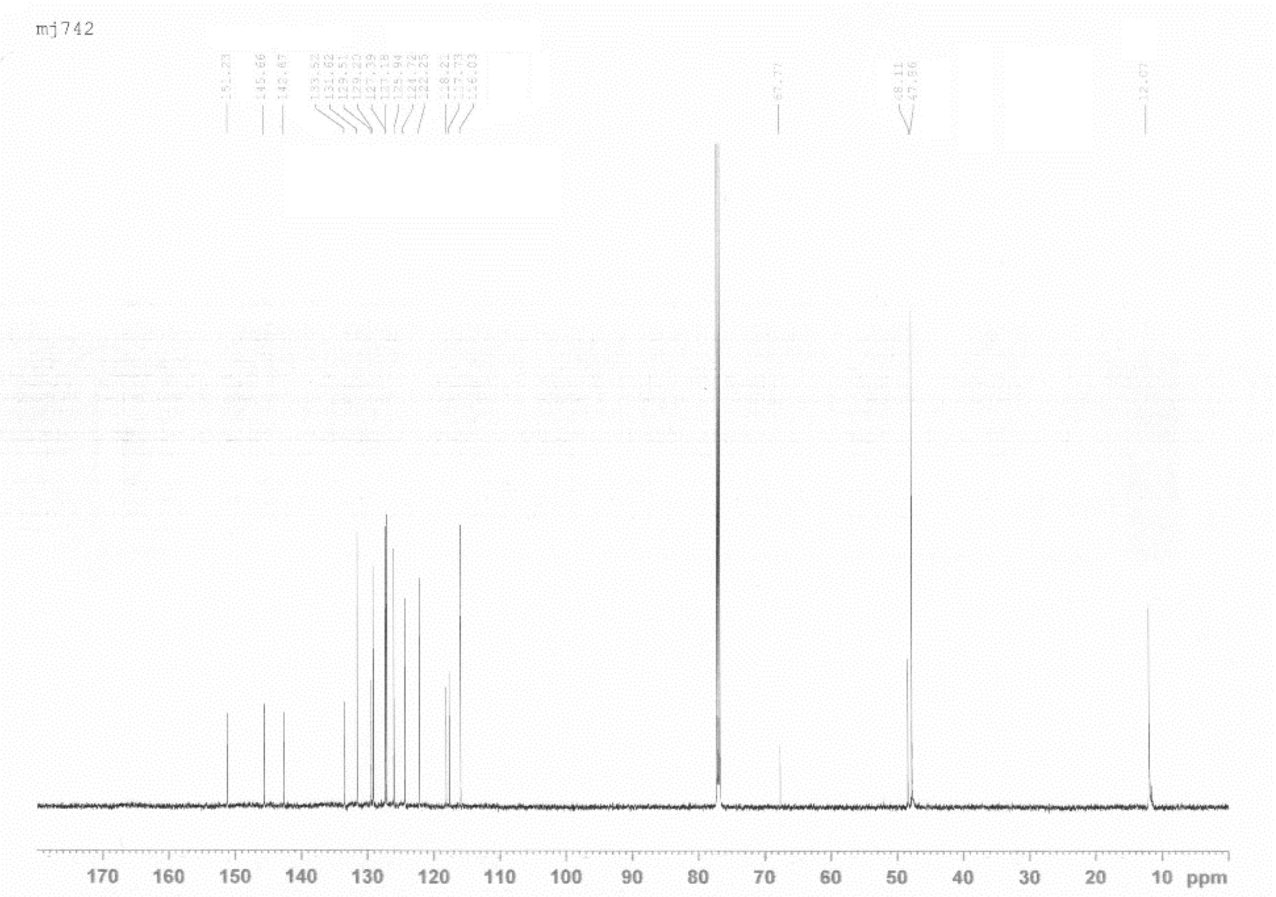

Acquisition Parameter

Source Type	ESI	Ion Polarity	Positive	Set Nebulizer	0.3 Bar
Focus	Not active	Set Capillary	4000 V	Set Dry Heater	200 °C
Scan Begin	100 m/z	Set End Plate Offset	-500 V	Set Dry Gas	3.0 l/min
Scan End	1000 m/z	Set Charging Voltage	2000 V	Set Divert Valve	Source
		Set Corona	0 nA	Set APCI Heater	0 °C

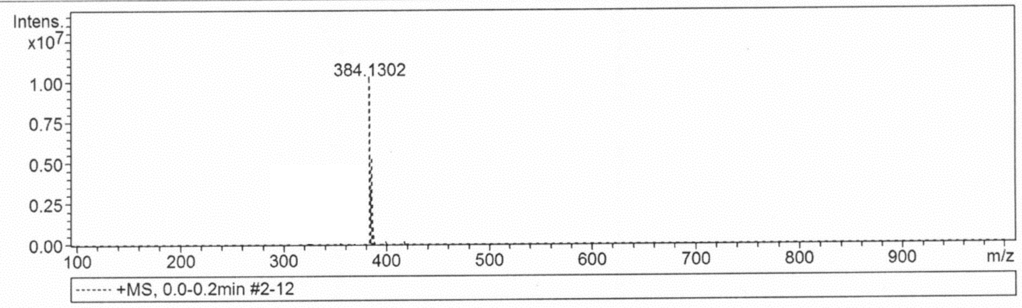

#	m/z	Res.	S/N	I	I %	FWHM
1	384.1302	10222	54428.6	10071745	100.0	0.0376
2	386.1272	13564	29049.0	5380588	53.4	0.0285

4. NMR spectra and HR MS of 3-(8-chloroquino[3,2-b]benzo[1,4]thiazin-6-yl)-N,N-dimethylpropan-1-amine (**8**).

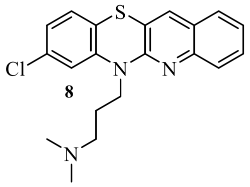

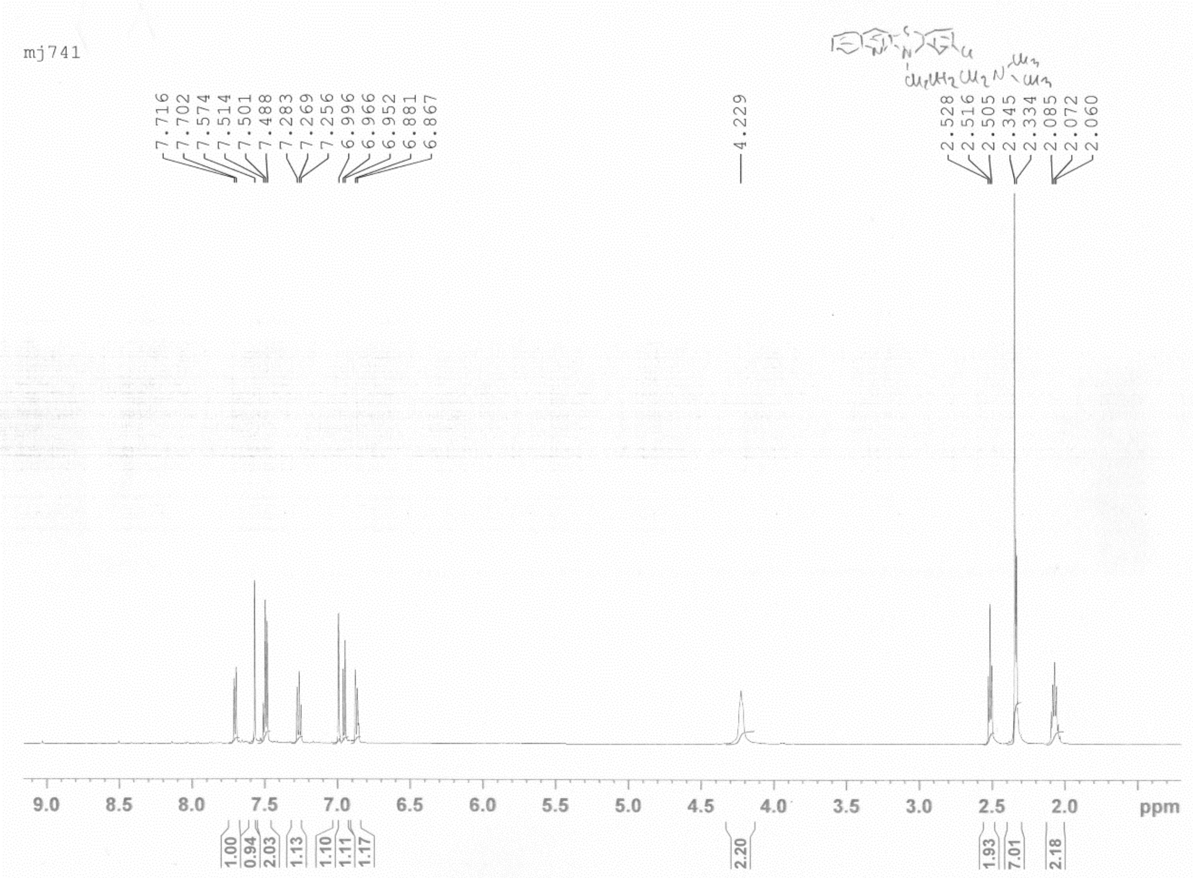

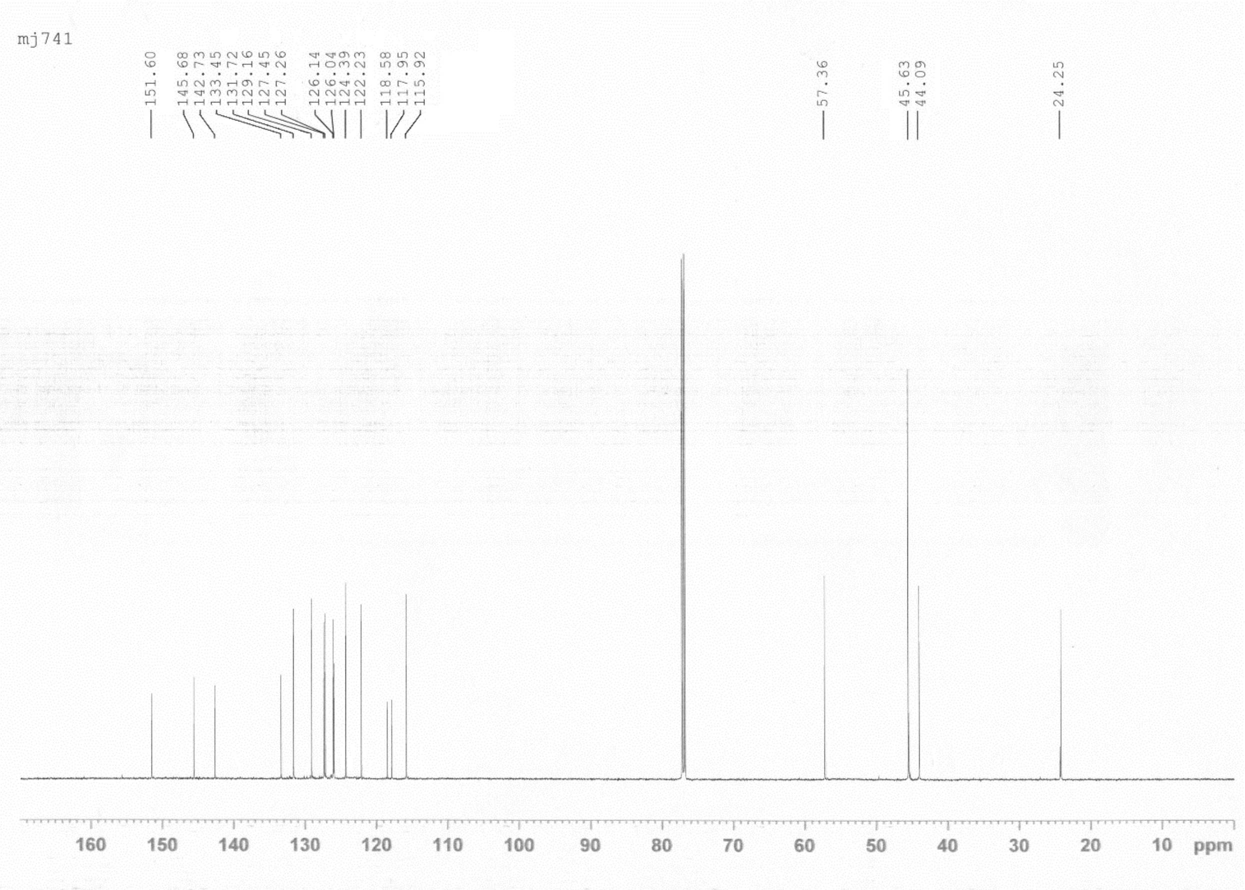

Acquisition Parameter

Source Type	ESI	Ion Polarity	Positive	Set Nebulizer	0.3 Bar
Focus	Not active	Set Capillary	4000 V	Set Dry Heater	200 °C
Scan Begin	100 m/z	Set End Plate Offset	-500 V	Set Dry Gas	3.0 l/min
Scan End	1000 m/z	Set Charging Voltage	2000 V	Set Divert Valve	Source
		Set Corona	0 nA	Set APCI Heater	0 °C

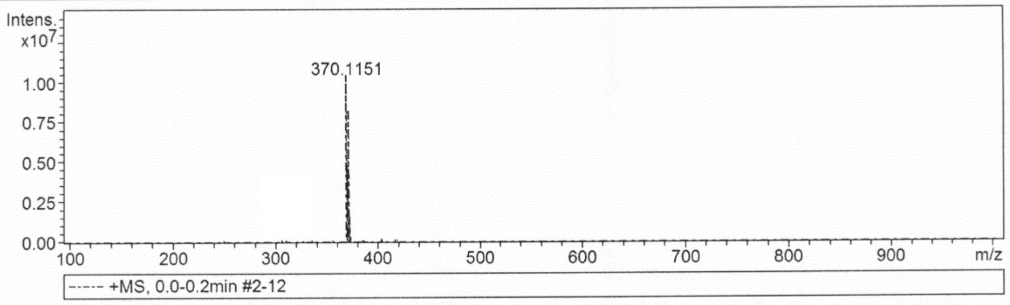

#	m/z	Res.	S/N	I	I %	FWHM
1	370.1151	8103	56136.2	10188798	100.0	0.0457
2	372.1123	13546	43737.8	7961009	78.1	0.0275

5. NMR spectra and HR MS of 8-chloro-6-(2-(pyrrolidin-1-yl)ethyl)-quino[3,2-b]benzo[1,4]thiazine (**9**).

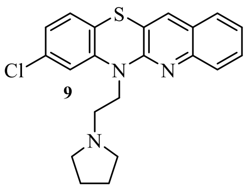

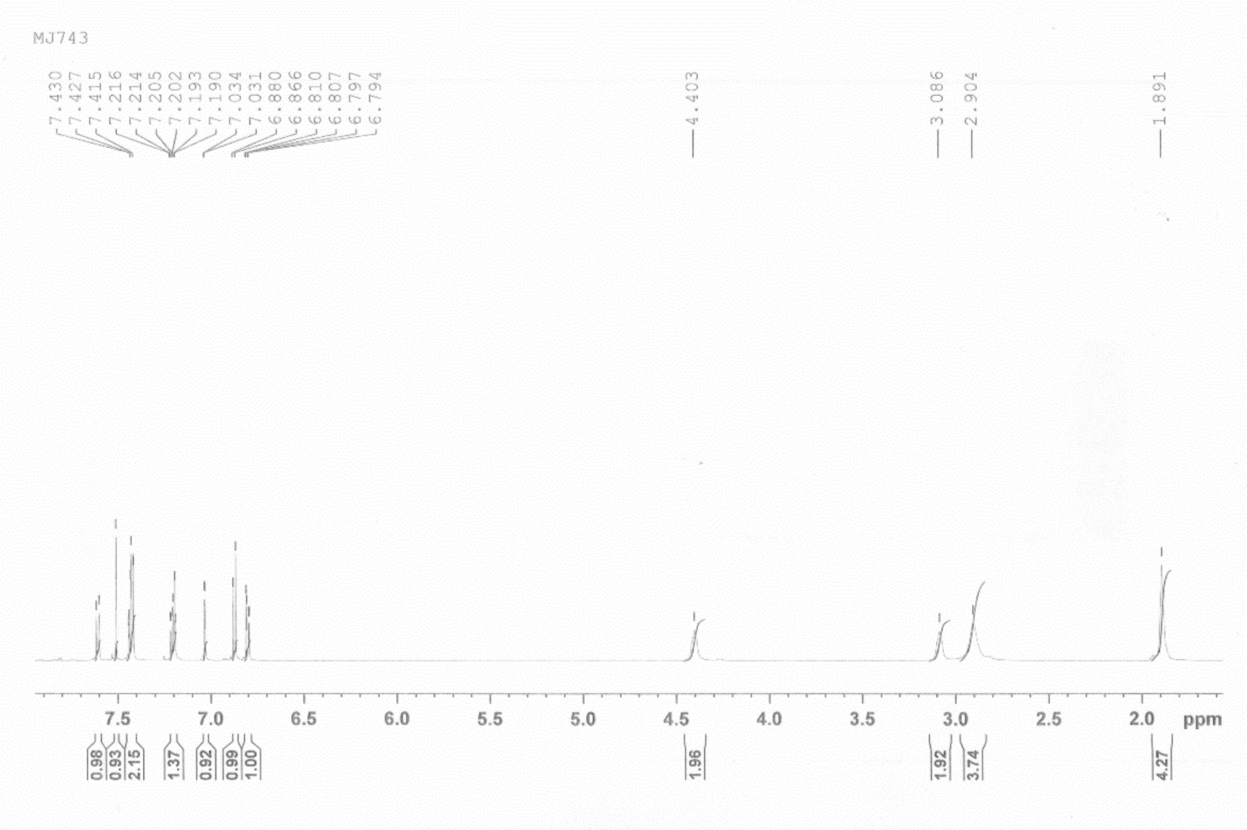

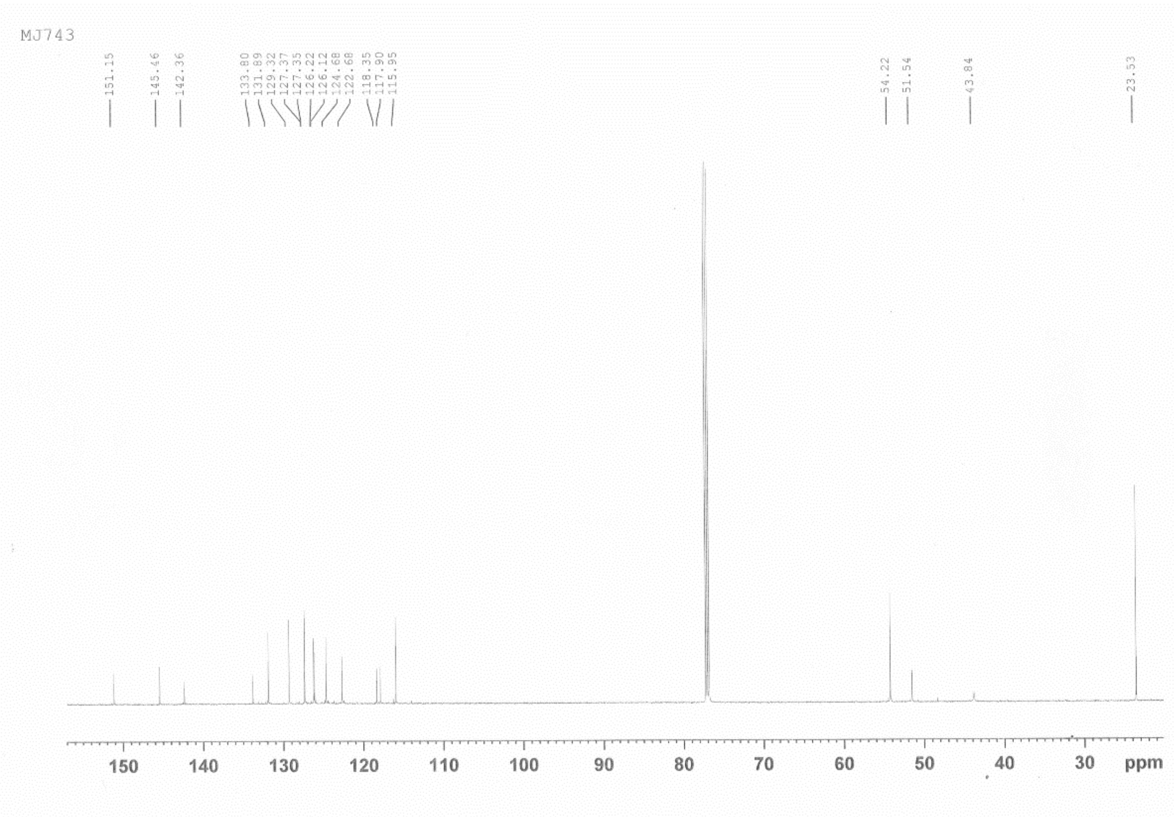

Acquisition Parameter

Source Type	ESI	Ion Polarity	Positive	Set Nebulizer	0.3 Bar
Focus	Not active	Set Capillary	4000 V	Set Dry Heater	200 °C
Scan Begin	100 m/z	Set End Plate Offset	-500 V	Set Dry Gas	3.0 l/min
Scan End	1000 m/z	Set Charging Voltage	2000 V	Set Divert Valve	Source
		Set Corona	0 nA	Set APCI Heater	0 °C

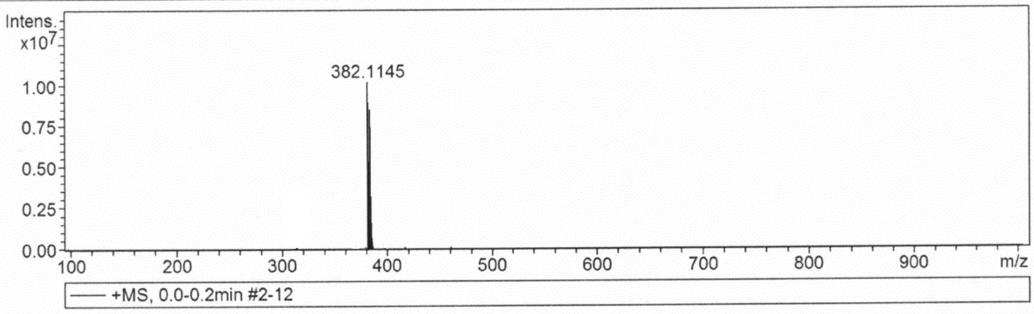

#	m/z	Res.	S/N	I	I %	FWHM
1	382.1145	7782	81007.5	10188798	100.0	0.0491
2	384.1118	13403	67270.0	8484336	83.3	0.0287

6. NMR spectra and HR MS of 8-chloro-6-(2-(piperidin-1-yl)ethyl)-quino[3,2-b]benzo[1,4]thiazine (**10**).

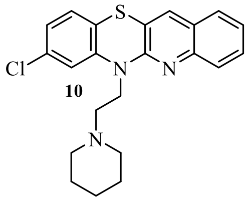

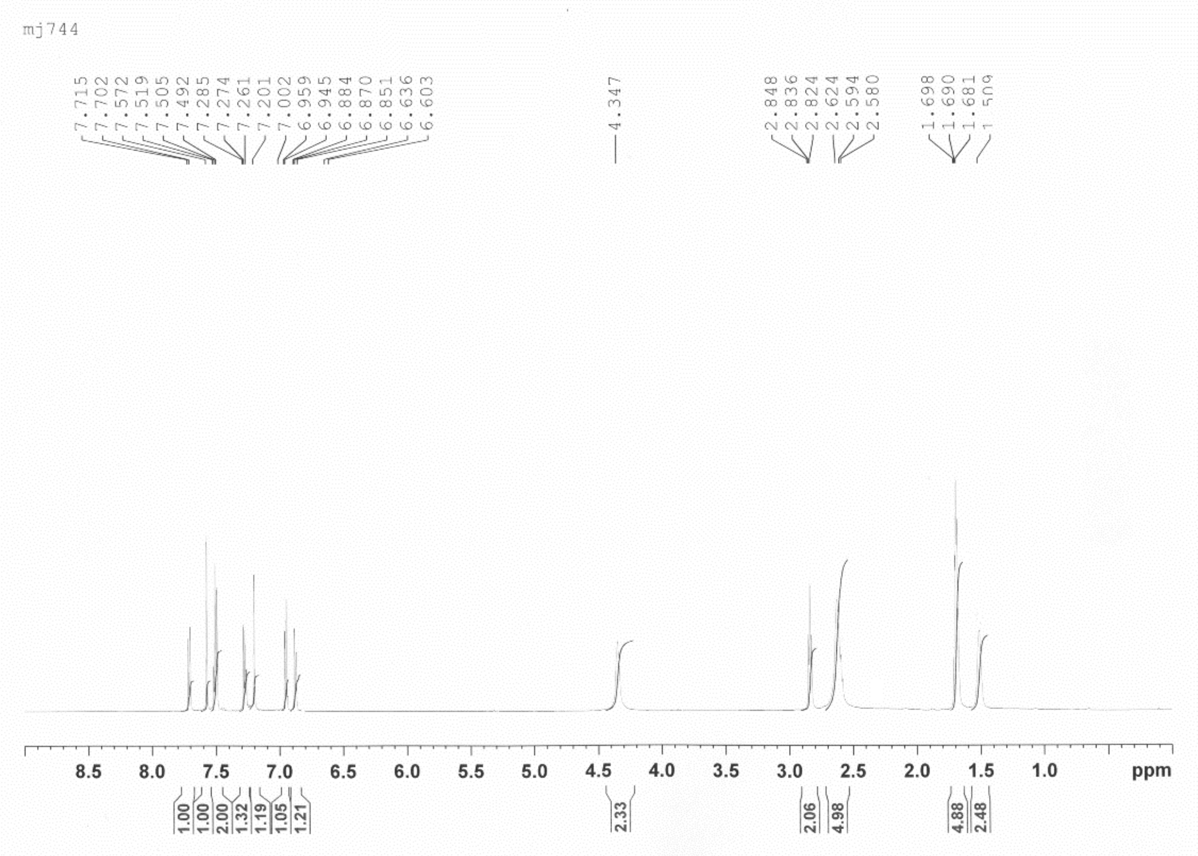

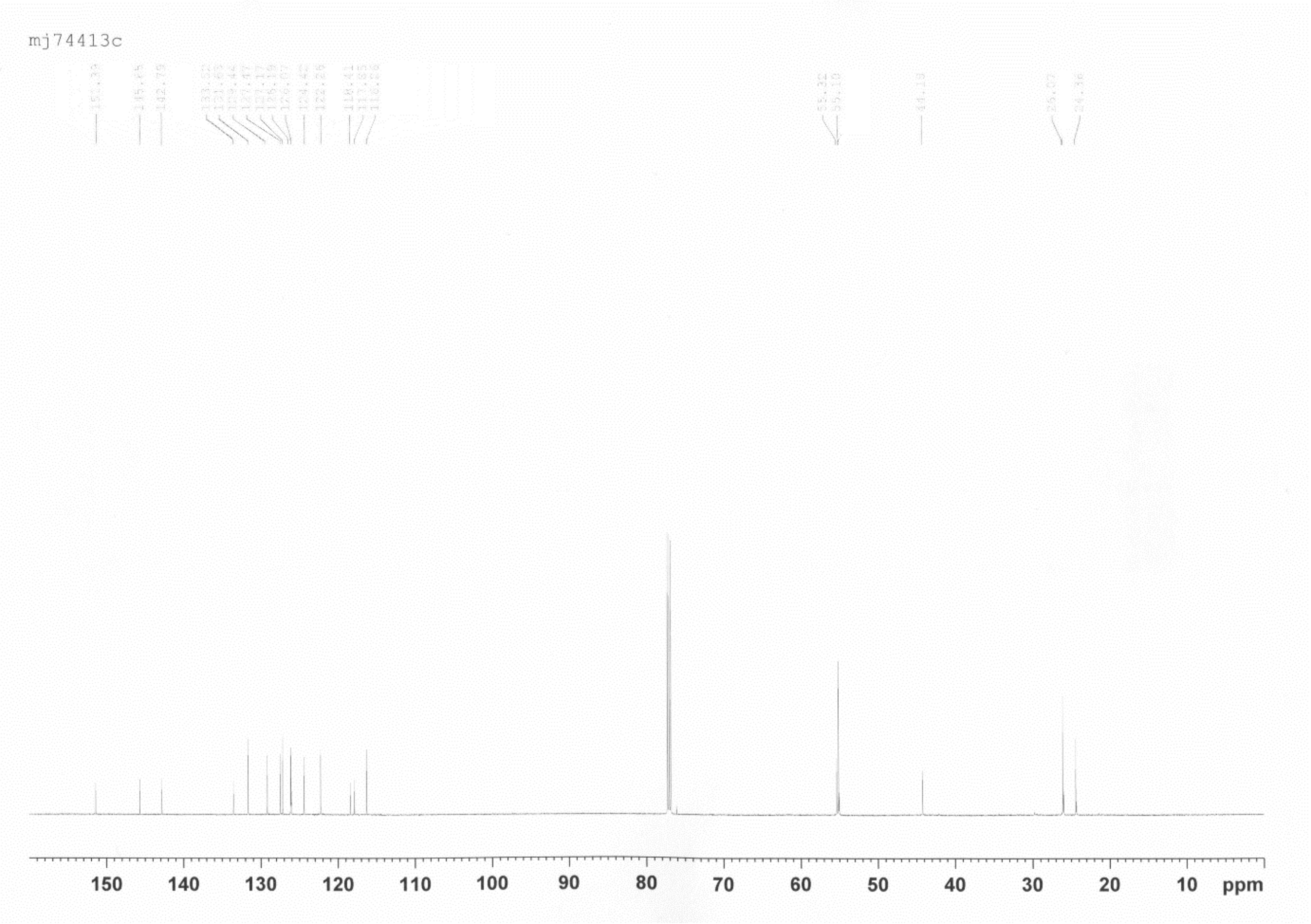

Acquisition Parameter

Source Type	ESI	Ion Polarity	Positive	Set Nebulizer	0.3 Bar
Focus	Not active	Set Capillary	4000 V	Set Dry Heater	200 °C
Scan Begin	100 m/z	Set End Plate Offset	-500 V	Set Dry Gas	3.0 l/min
Scan End	1000 m/z	Set Charging Voltage	2000 V	Set Divert Valve	Source
		Set Corona	0 nA	Set APCI Heater	0 °C

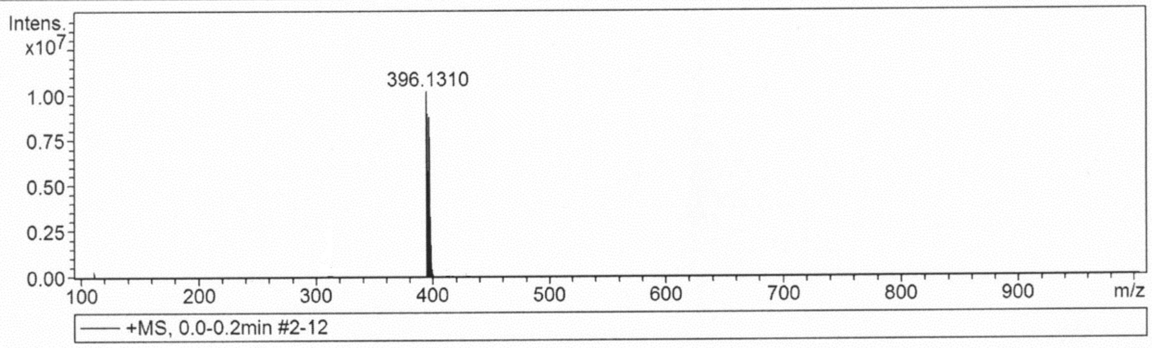

#	m/z	Res.	S/N	I	I %	FWHM
1	396.1310	7738	94773.6	10188798	100.0	0.0512
2	398.1282	13593	81523.1	8789789	86.3	0.0293

7. NMR spectra and HR MS of 8-chloro-6-(2-(1-methylpiperidin-2-yl)ethyl)-quino[3,2-b]benzo[1,4]thiazine (**11**).

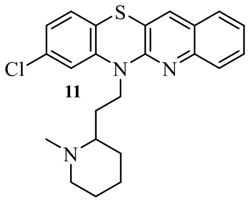

11

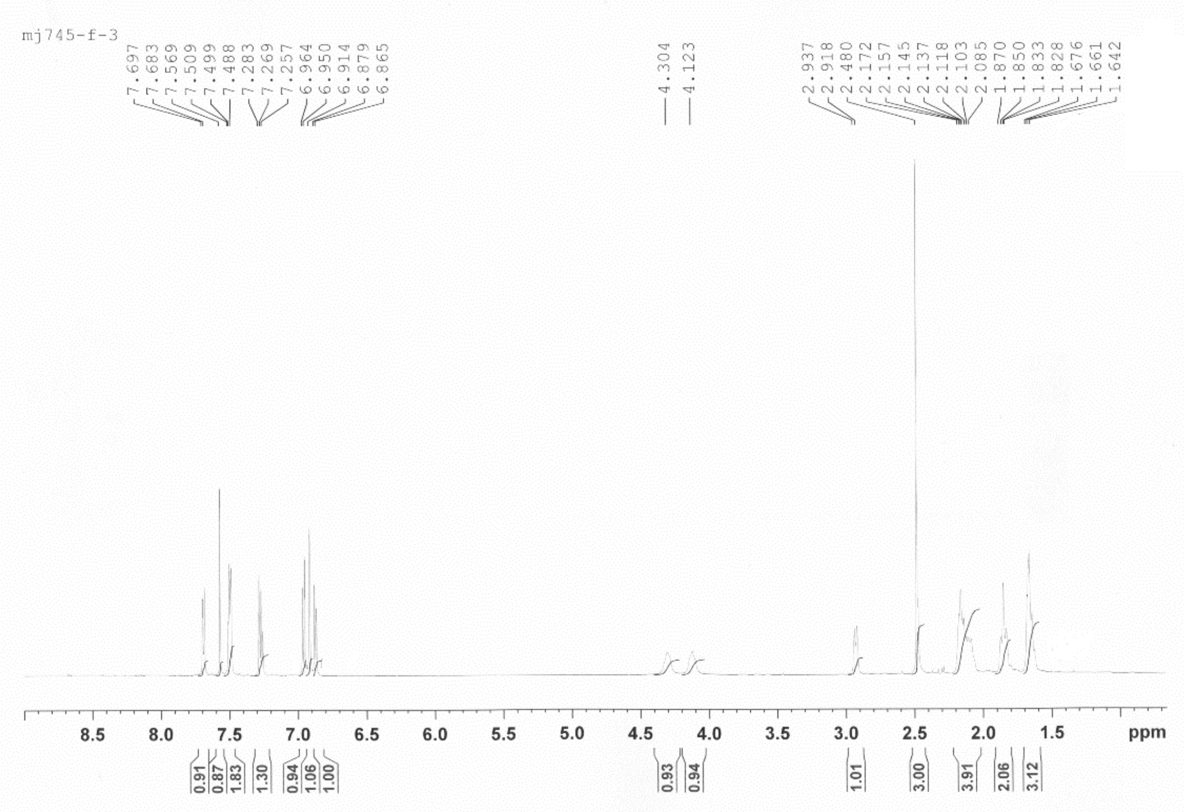

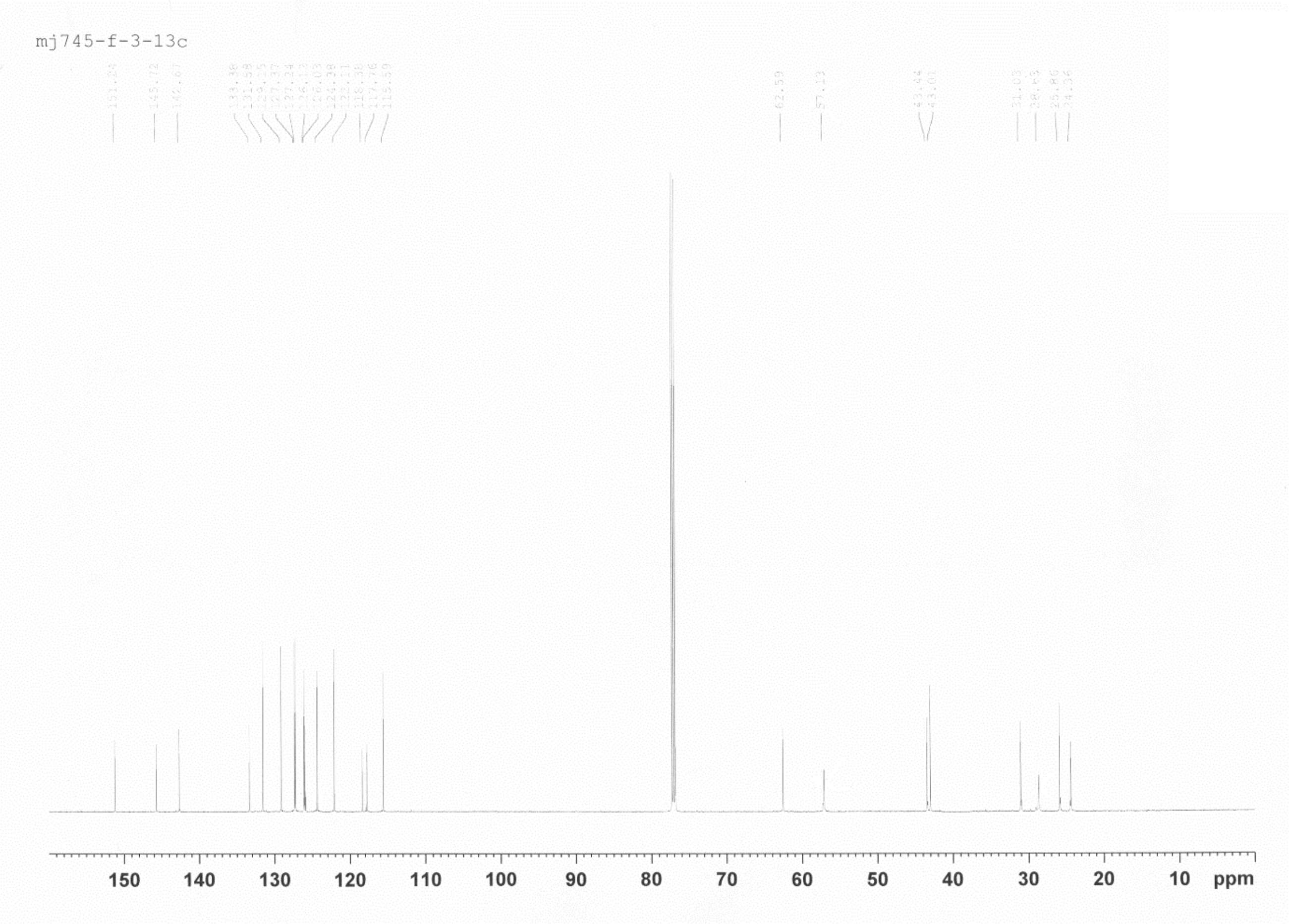

Acquisition Parameter

Source Type	ESI	Ion Polarity	Positive	Set Nebulizer	0.3 Bar
Focus	Not active	Set Capillary	4000 V	Set Dry Heater	200 °C
Scan Begin	100 m/z	Set End Plate Offset	-500 V	Set Dry Gas	3.0 l/min
Scan End	1000 m/z	Set Charging Voltage	2000 V	Set Divert Valve	Source
		Set Corona	0 nA	Set APCI Heater	0 °C

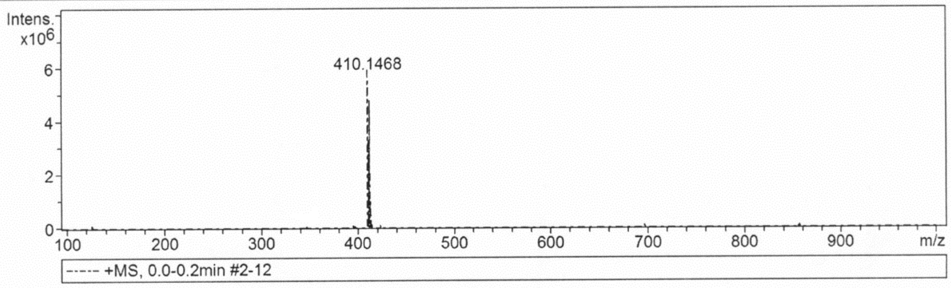

#	m/z	Res.	S/N	I	I %	FWHM
1	410.1468	8116	88496.9	5767489	100.0	0.0505
2	412.1442	13609	70653.7	4628476	80.3	0.0303

8. NMR spectra and HR MS of 2-(3-(8-chloroquino[3,2-b]benzo[1,4]thiazin-6-yl)propyl)isoindoline-1,3-dione (**12**).

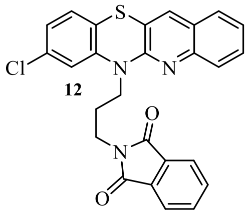

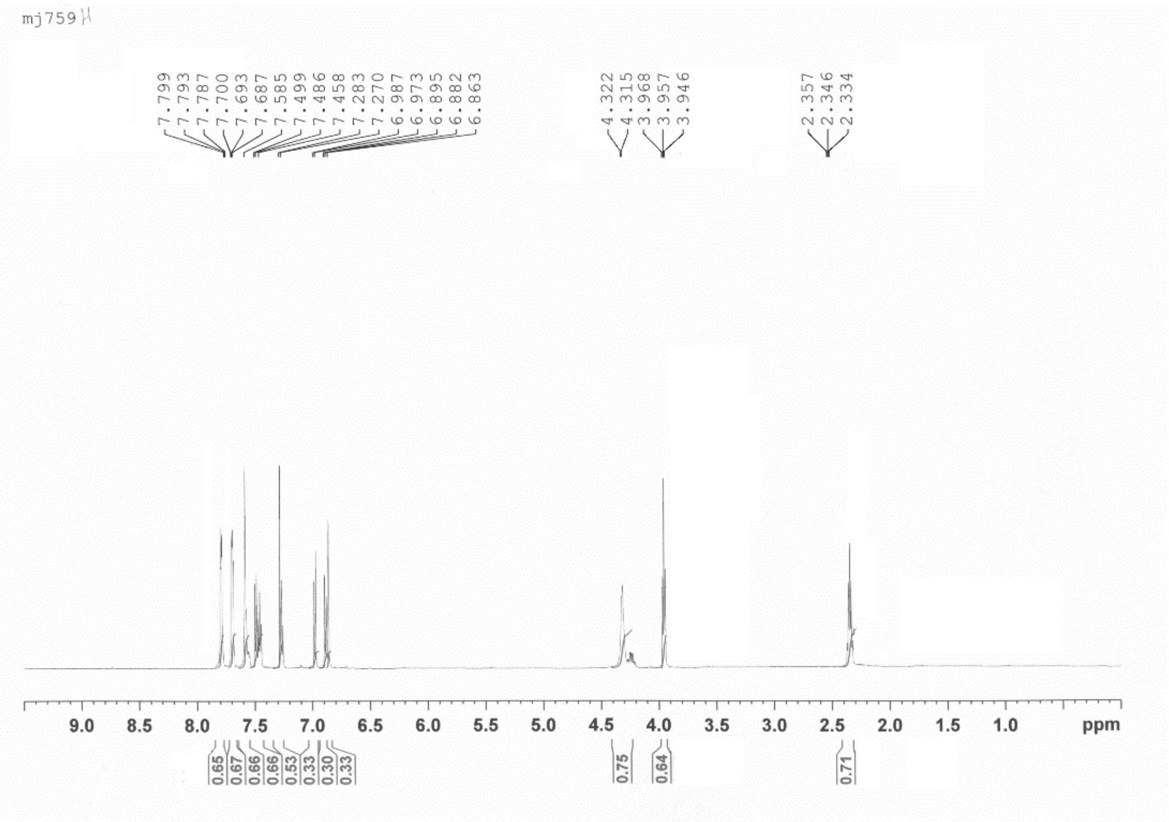

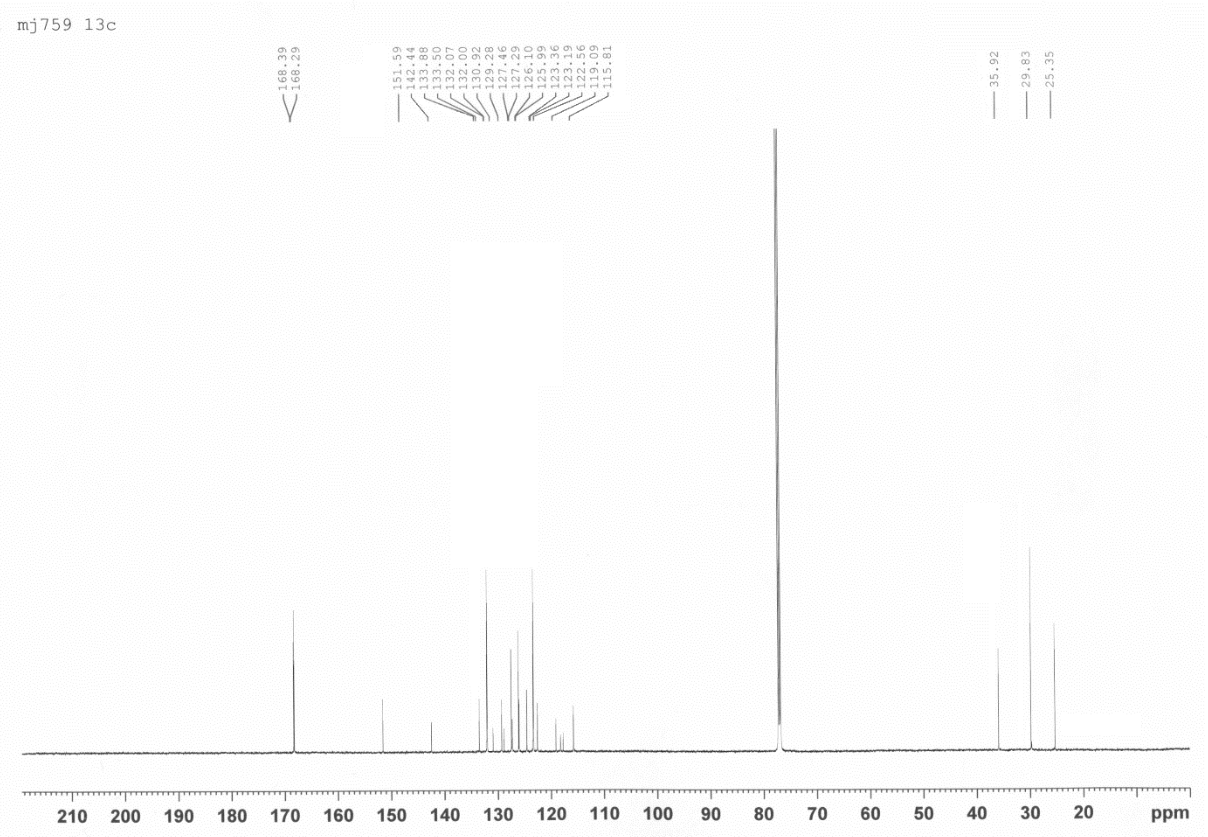

Acquisition Parameter

Source Type	ESI	Ion Polarity	Positive	Set Nebulizer	0.3 Bar
Focus	Active	Set Capillary	4000 V	Set Dry Heater	240 °C
Scan Begin	100 m/z	Set End Plate Offset	-500 V	Set Dry Gas	4.0 l/min
Scan End	500 m/z	Set Charging Voltage	2000 V	Set Divert Valve	Source
		Set Corona	0 nA	Set APCI Heater	0 °C

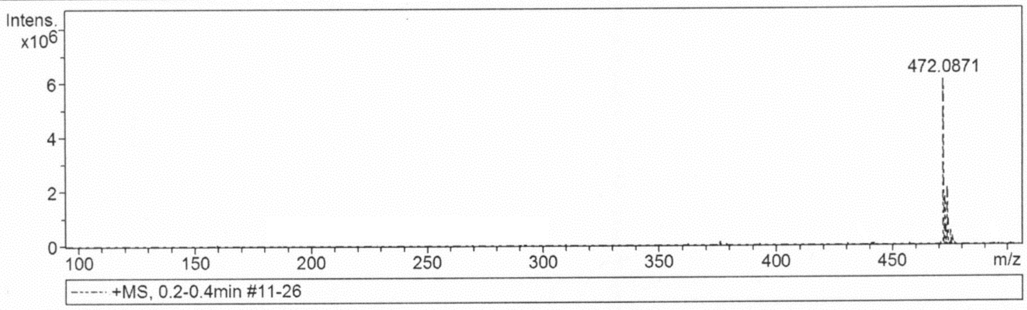

#	m/z	Res.	S/N	I	I %	FWHM
1	472.0871	37225	12528.0	6128841	100.0	0.0127
2	474.0836	26588	4409.6	2159305	35.2	0.0178

9. NMR spectra and HR MS of 3-(8-chloroquino[3,2-b]benzo[1,4]thiazin-6-yl)propan-1-amine (**14**).

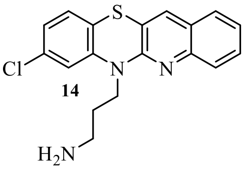

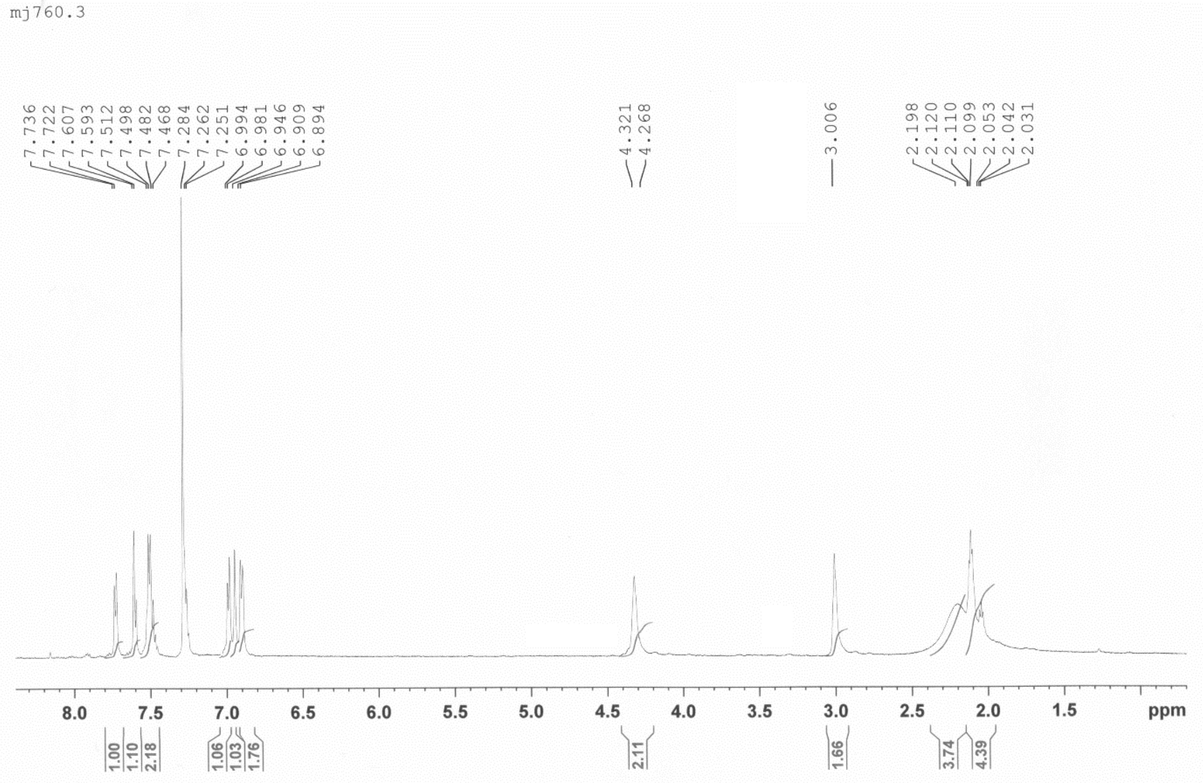

Acquisition Parameter

Source Type	ESI	Ion Polarity	Positive	Set Nebulizer	0.3 Bar
Focus	Active	Set Capillary	4000 V	Set Dry Heater	200 °C
Scan Begin	100 m/z	Set End Plate Offset	-500 V	Set Dry Gas	3.0 l/min
Scan End	1000 m/z	Set Charging Voltage	2000 V	Set Divert Valve	Source
		Set Corona	0 nA	Set APCI Heater	0 °C

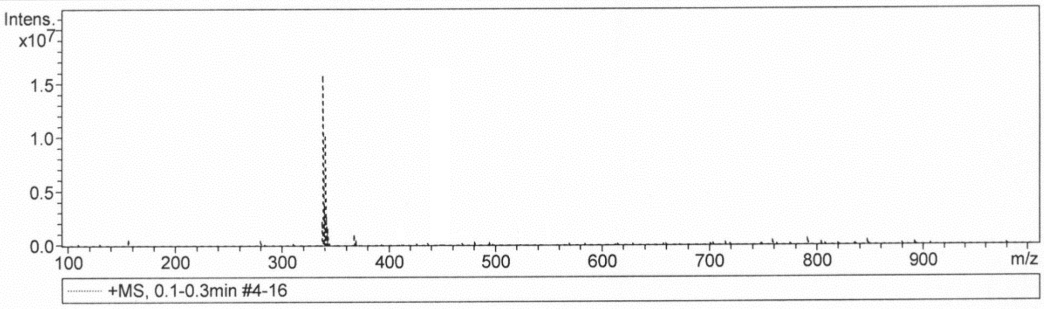

#	m/z	Res.	S/N	I	I %	FWHM
1	342.0844	23653	278.3	110821	100.0	0.0145

10. NMR spectra and HR MS of N-(3-(8-chloroquino[3,2-b]benzo[1,4]thiazin-6-yl)propyl)acetamide (**16**).

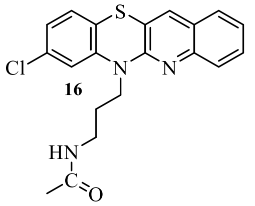

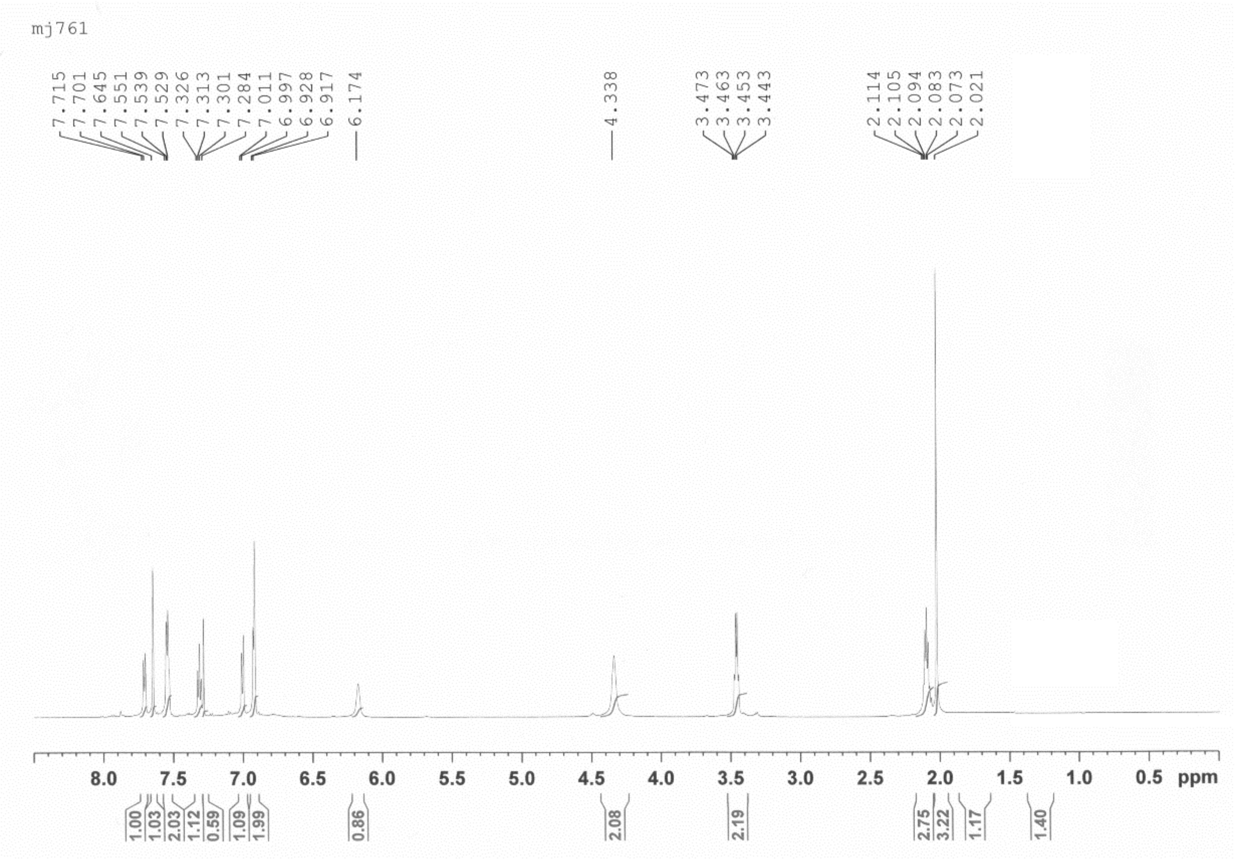

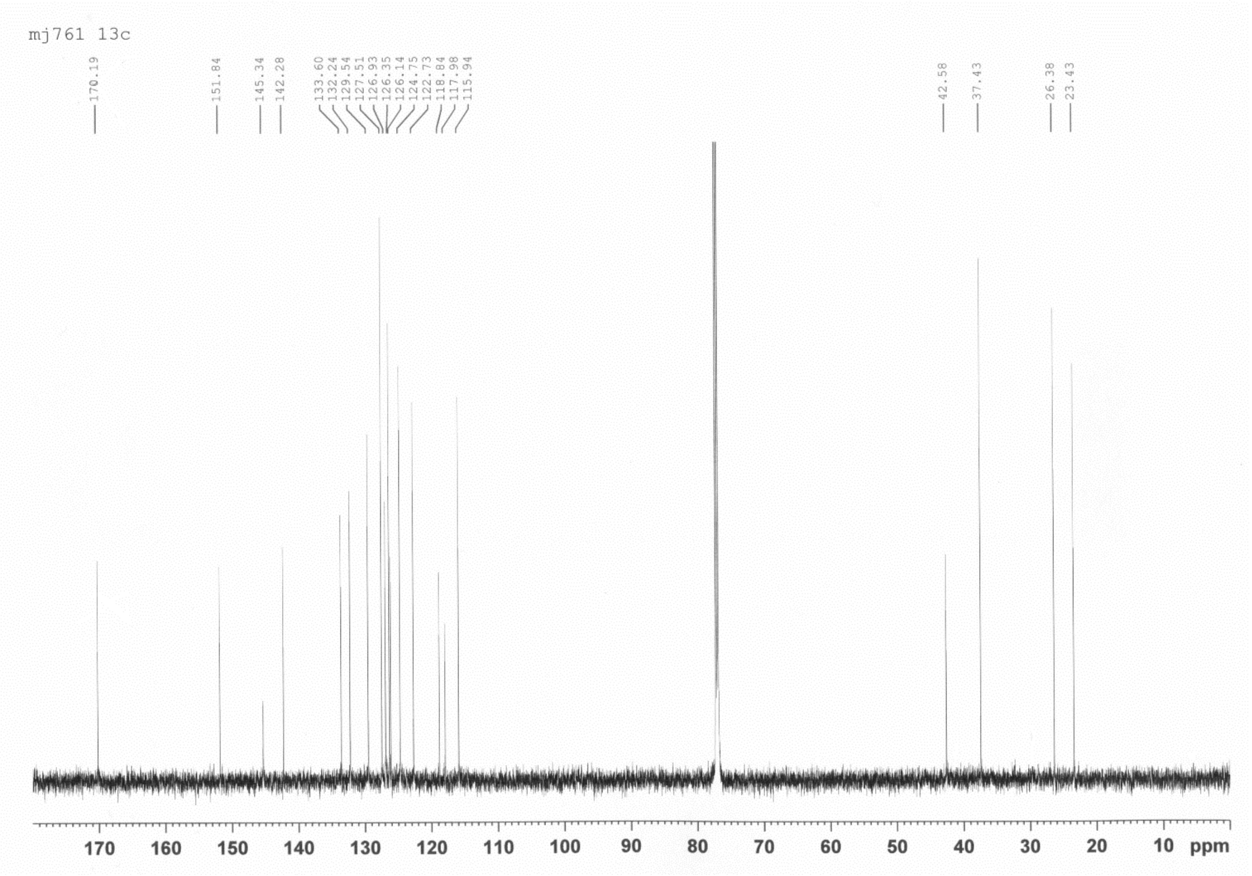

Acquisition Parameter

Source Type	ESI	Ion Polarity	Positive	Set Nebulizer	0.3 Bar
Focus	Active	Set Capillary	4000 V	Set Dry Heater	240 °C
Scan Begin	100 m/z	Set End Plate Offset	-500 V	Set Dry Gas	4.0 l/min
Scan End	500 m/z	Set Charging Voltage	2000 V	Set Divert Valve	Source
		Set Corona	0 nA	Set APCI Heater	0 °C

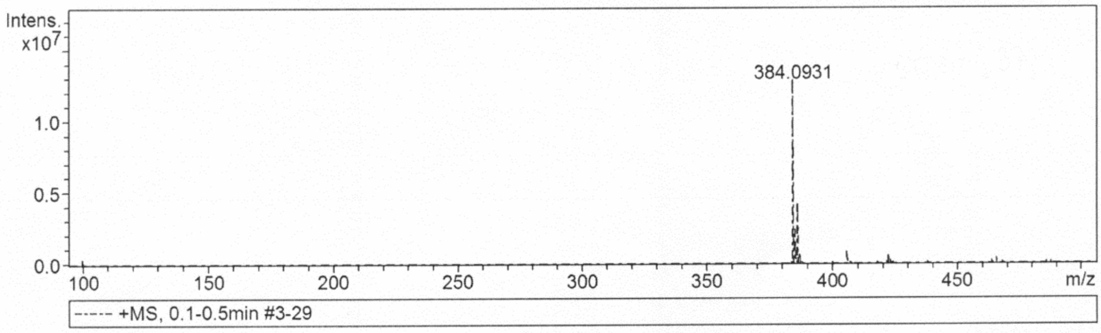

#	m/z	Res.	S/N	I	I %	FWHM
1	384.0931	38530	80379.1	12531551	100.0	0.0100
2	386.0898	32092	27159.5	4284578	34.2	0.0120

11. NMR spectra and HR MS of ethyl(3-(8-chloro-quino[3,2-b]benzo[1,4]thiazin-6-yl)propyl)carbamate (**18**).

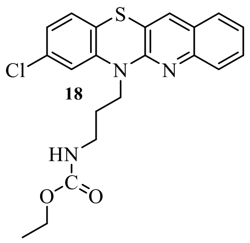

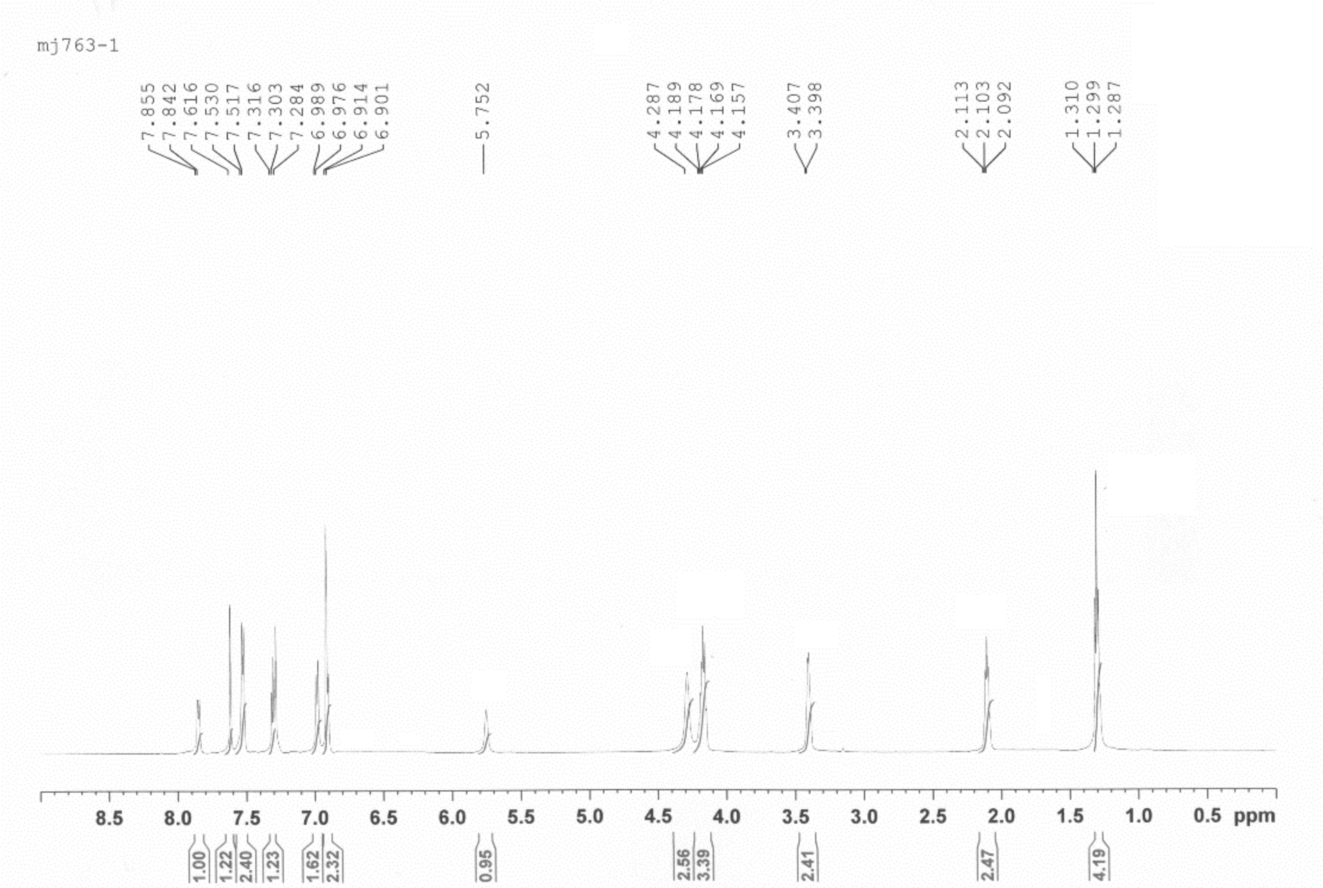

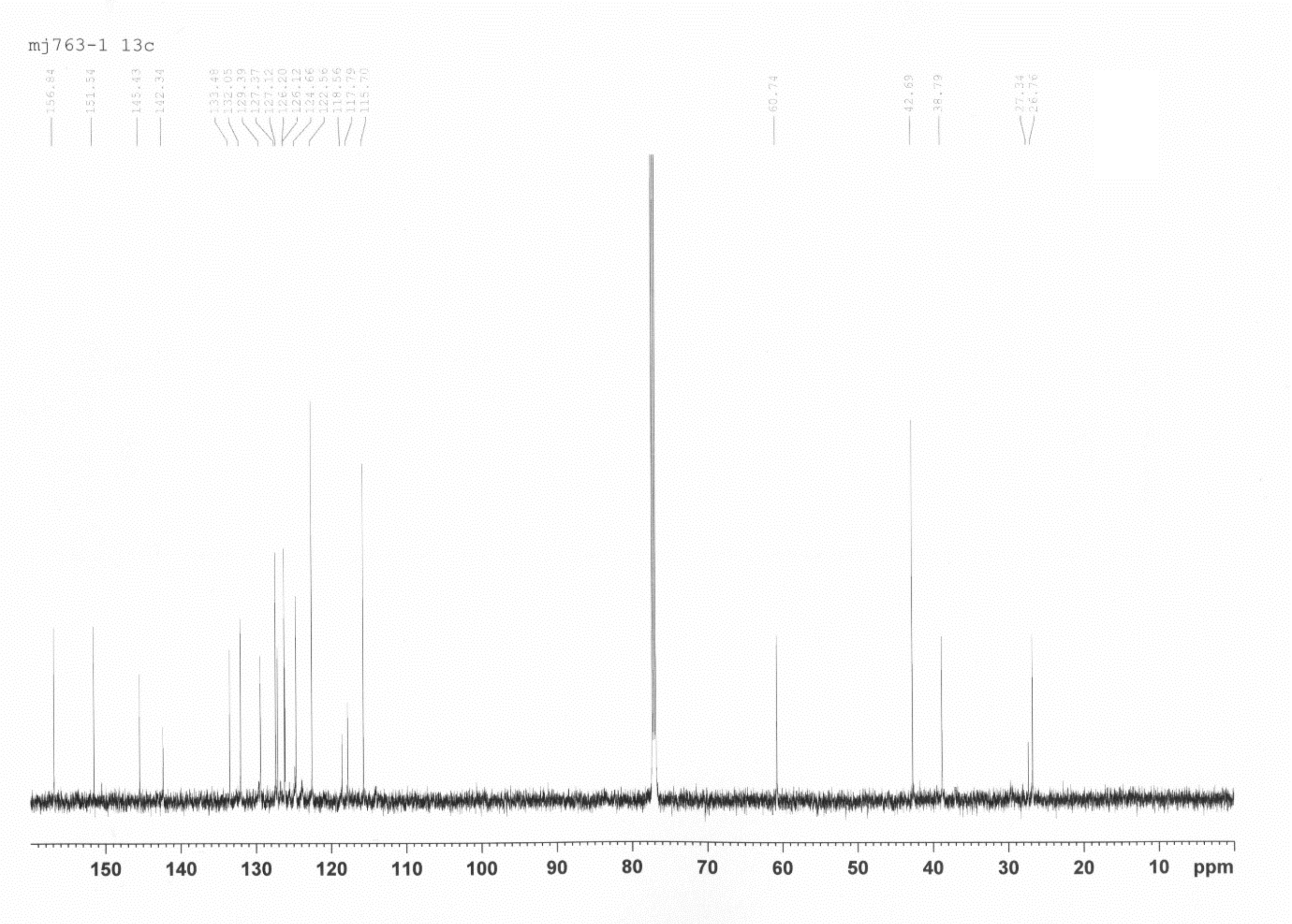

Acquisition Parameter

Source Type	ESI	Ion Polarity	Positive	Set Nebulizer	0.3 Bar
Focus	Active	Set Capillary	4000 V	Set Dry Heater	240 °C
Scan Begin	100 m/z	Set End Plate Offset	-500 V	Set Dry Gas	4.0 l/min
Scan End	500 m/z	Set Charging Voltage	2000 V	Set Divert Valve	Source
		Set Corona	0 nA	Set APCI Heater	0 °C

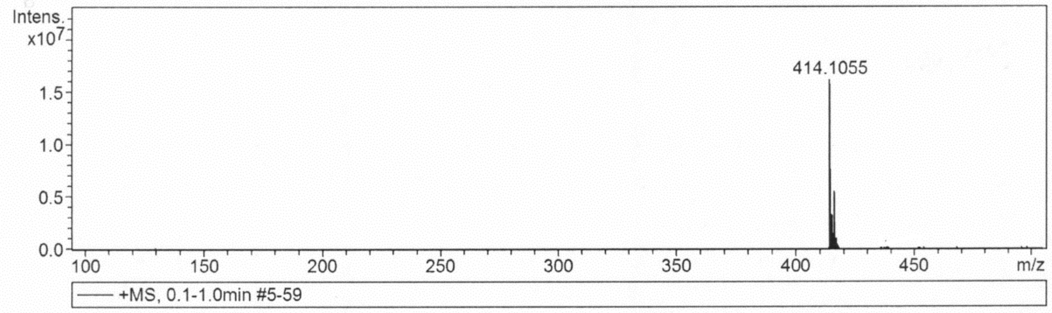

#	m/z	Res.	S/N	I	I %	FWHM
1	414.1055	34600	96812.6	16221700	100.0	0.0120
2	416.1021	33931	32773.3	5547655	34.2	0.0123

12. NMR spectra and HR MS of 1-(3-(8-chloroquino[3,2-b]benzo[1,4]thiazin-6-yl)propyl)-3-(2-chloroethyl)urea (**20**).

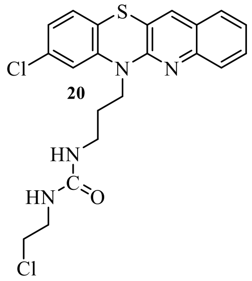

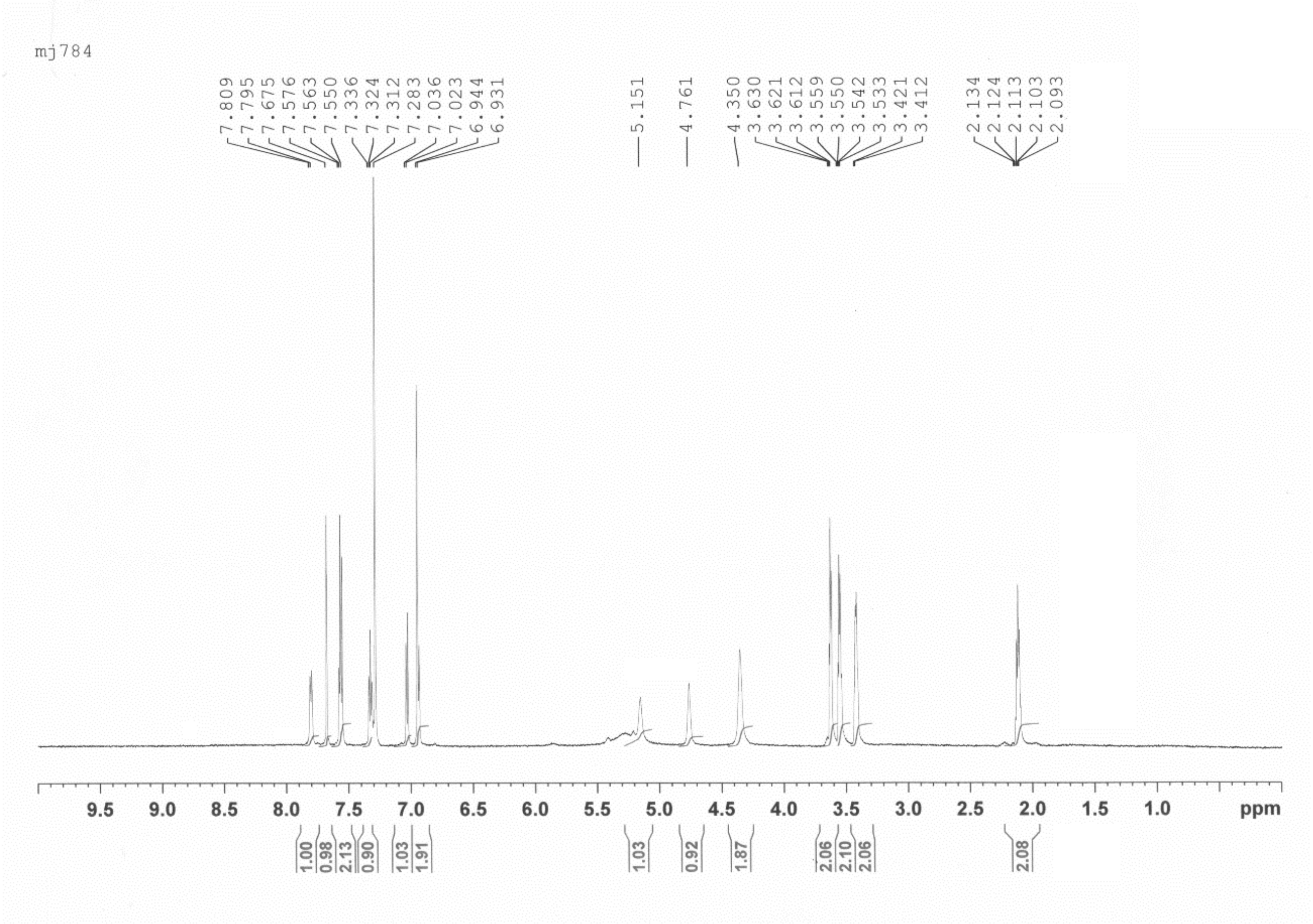

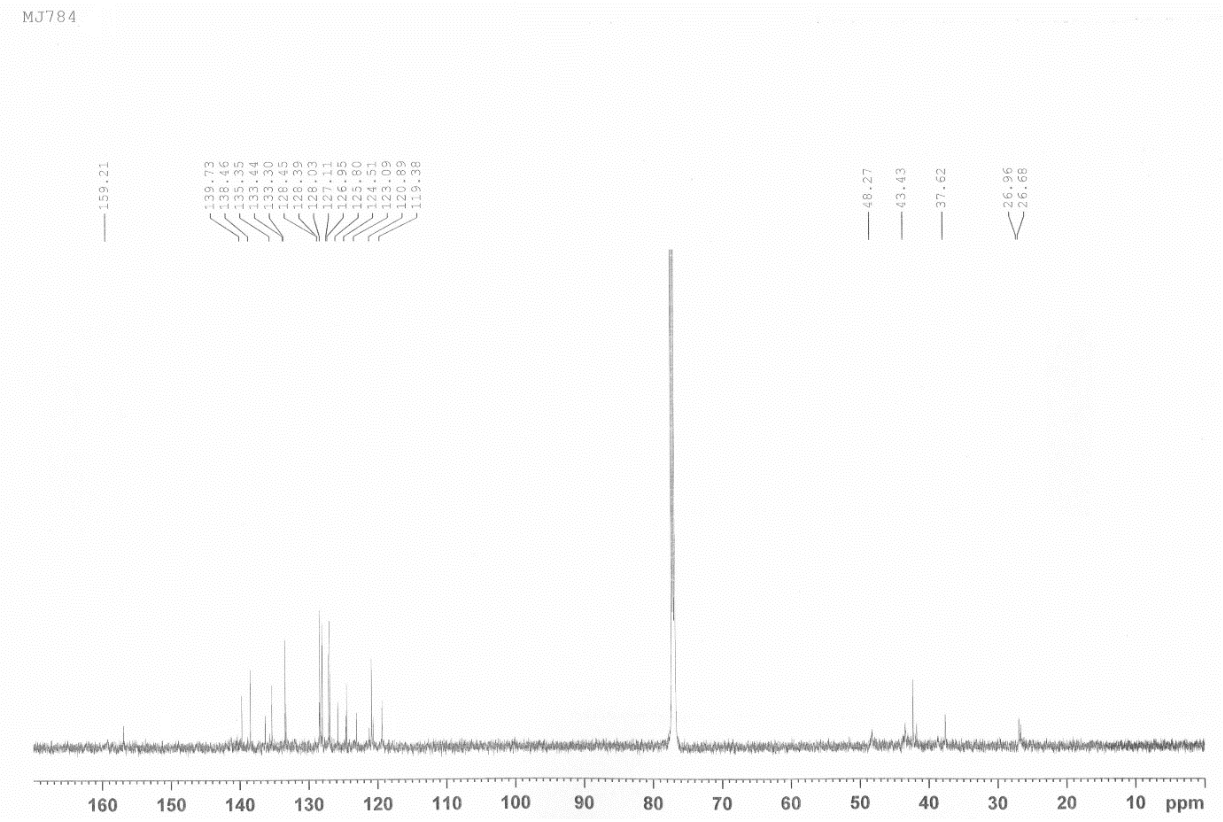

Acquisition Parameter

Source Type	ESI	Ion Polarity	Positive	Set Nebulizer	0.3 Bar
Focus	Active	Set Capillary	4000 V	Set Dry Heater	200 °C
Scan Begin	100 m/z	Set End Plate Offset	-500 V	Set Dry Gas	3.0 l/min
Scan End	1000 m/z	Set Charging Voltage	2000 V	Set Divert Valve	Source
		Set Corona	0 nA	Set APCI Heater	0 °C

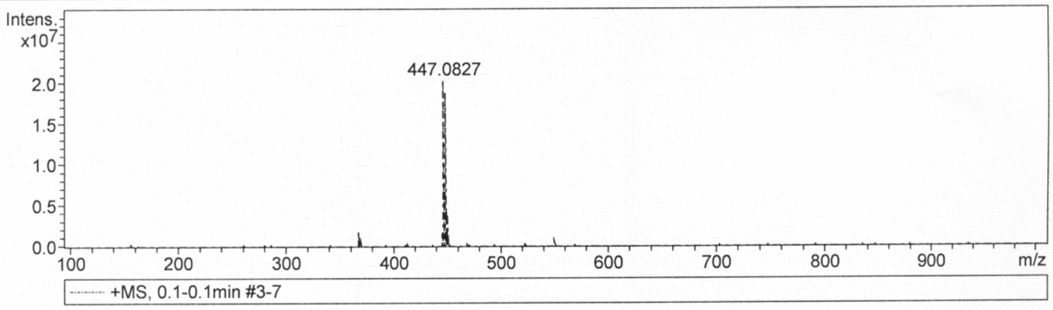

#	m/z	Res.	S/N	I	I %	FWHM
1	447.0827	22029	37433.2	20371780	100.0	0.0203
2	449.0793	39034	33448.2	18212446	89.4	0.0115

13. NMR spectra and HR MS of N-(3-(8-chloroquino[3,2-b]benzo[1,4]thiazin-6-yl)propyl)methanesulfonamide (**22**).

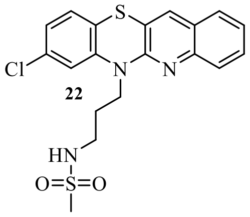

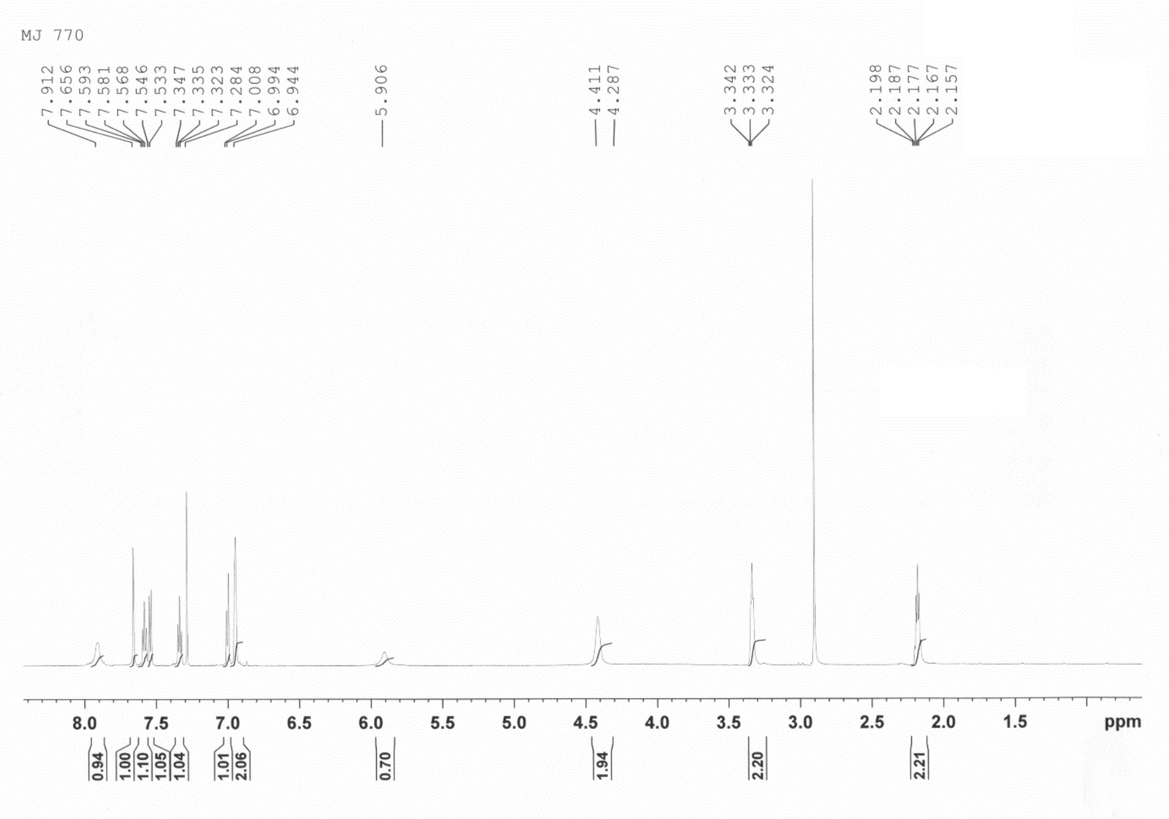

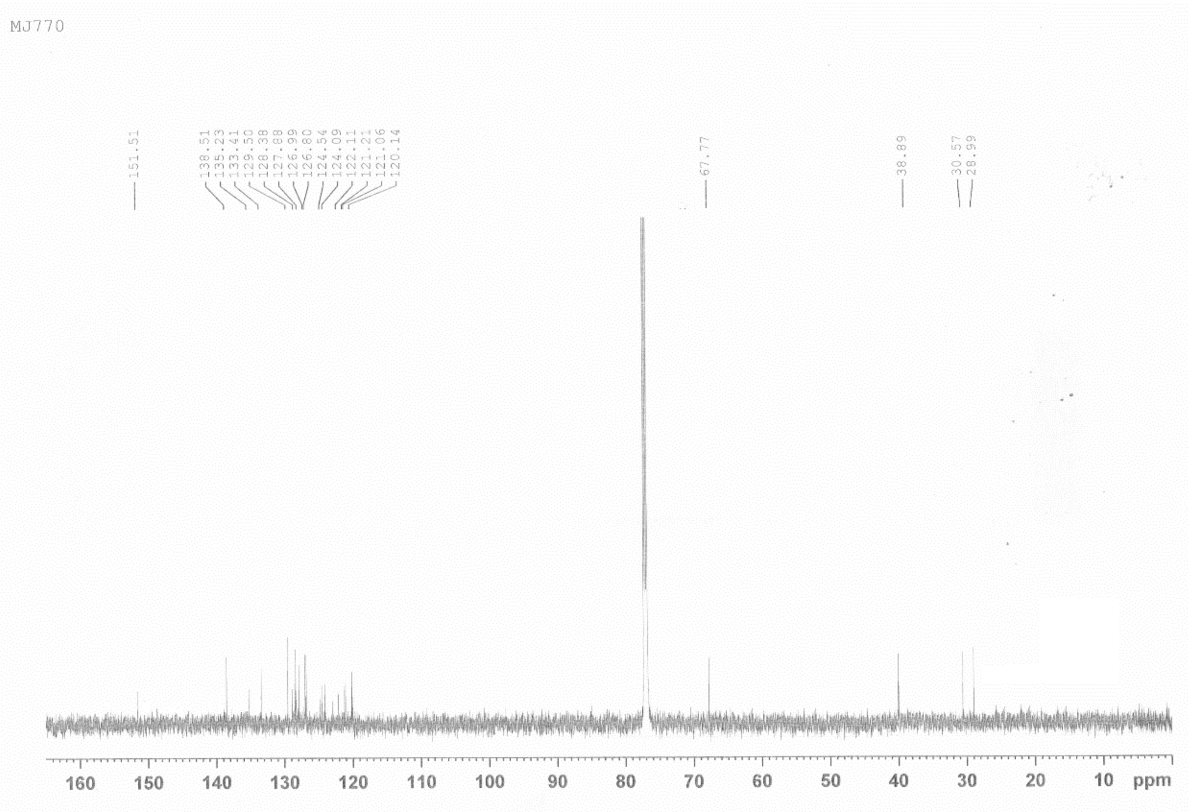

Acquisition Parameter

Source Type	ESI	Ion Polarity	Positive	Set Nebulizer	0.3 Bar
Focus	Active	Set Capillary	4000 V	Set Dry Heater	240 °C
Scan Begin	100 m/z	Set End Plate Offset	-500 V	Set Dry Gas	4.0 l/min
Scan End	500 m/z	Set Charging Voltage	2000 V	Set Divert Valve	Source
		Set Corona	0 nA	Set APCI Heater	0 °C

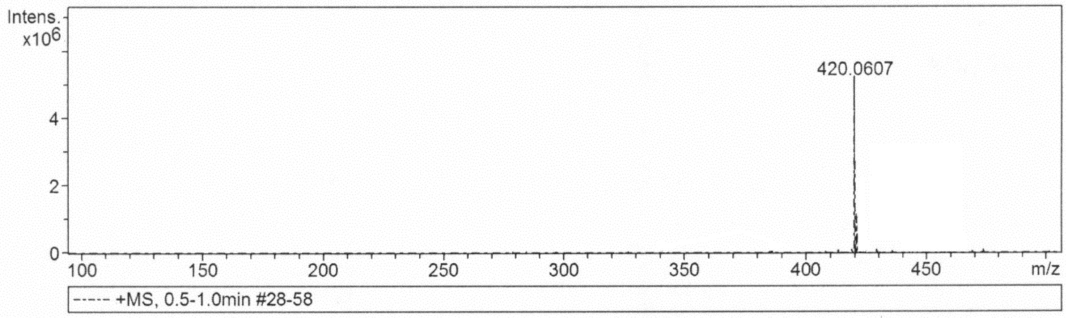

#	m/z	Res.	S/N	I	I %	FWHM
1	420.0607	34065	20988.5	5131382	100.0	0.0123
2	422.0575	26272	7957.4	1969690	38.4	0.0161

14. NMR spectra and HR MS of N-(3-(8-chloroquino[3,2-b]benzo[1,4]thiazin-6-yl)propyl)-4-methylbenzenesulfonamide (**24**).

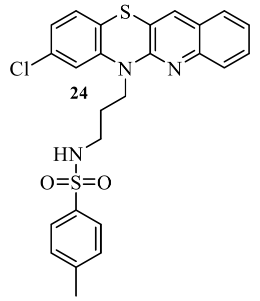

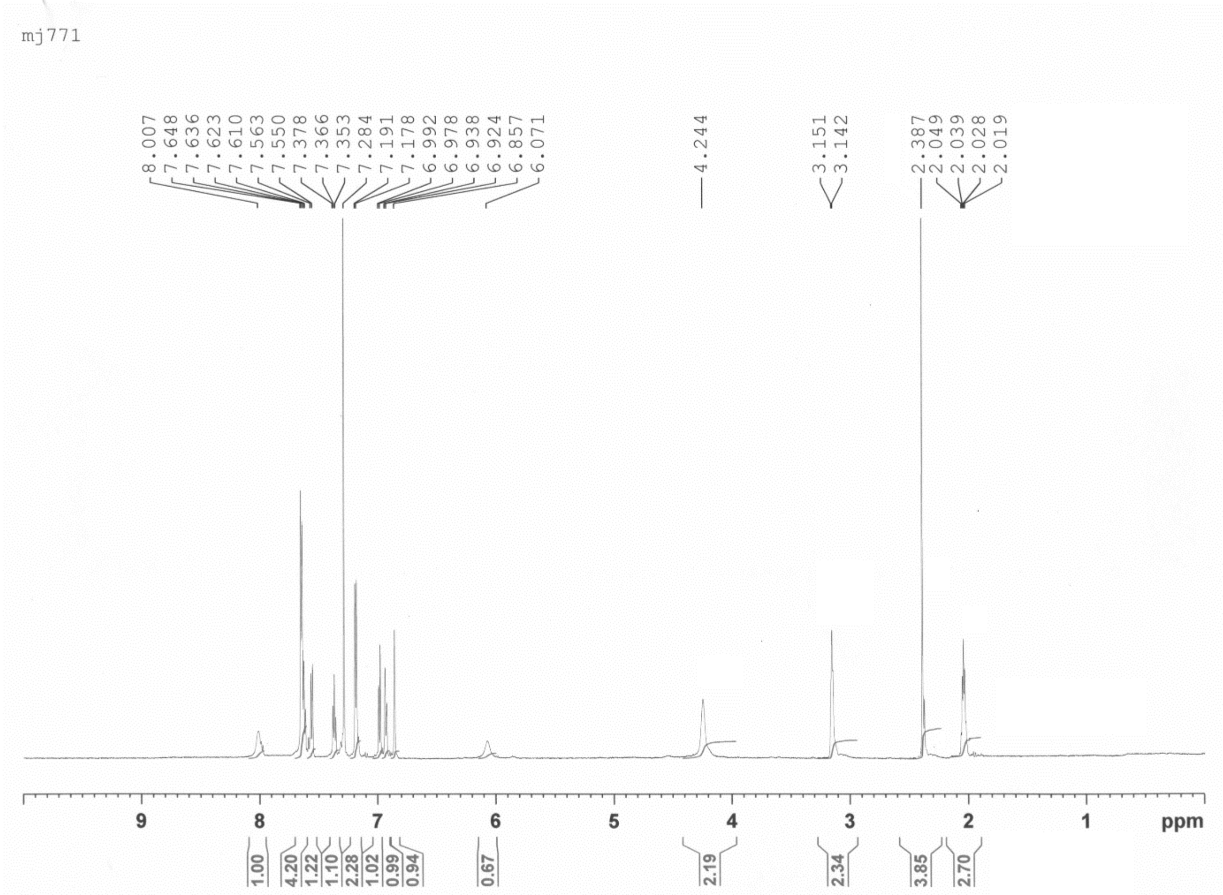

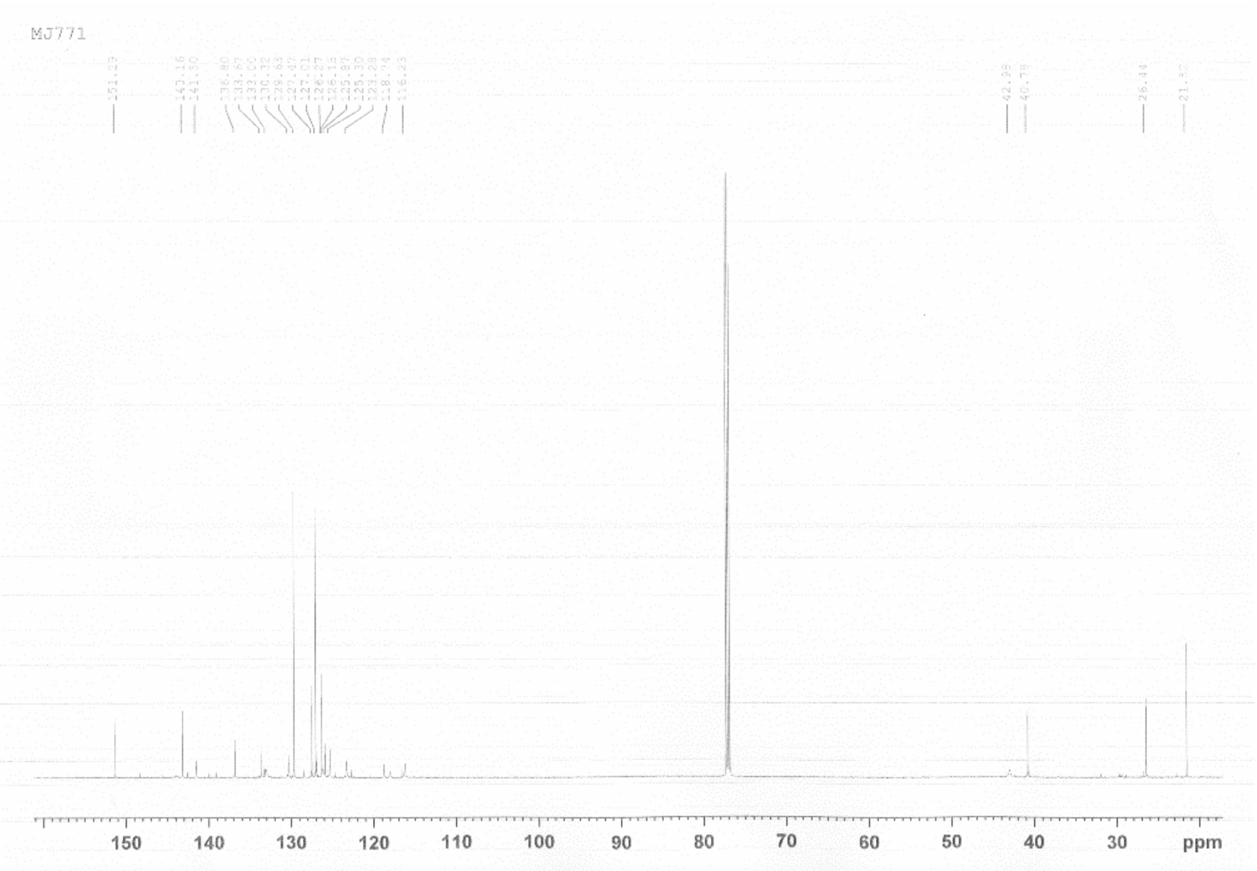

Acquisition Parameter

Source Type	ESI	Ion Polarity	Positive	Set Nebulizer	0.3 Bar
Focus	Active	Set Capillary	4000 V	Set Dry Heater	240 °C
Scan Begin	100 m/z	Set End Plate Offset	-500 V	Set Dry Gas	4.0 l/min
Scan End	500 m/z	Set Charging Voltage	2000 V	Set Divert Valve	Source
		Set Corona	0 nA	Set APCI Heater	0 °C

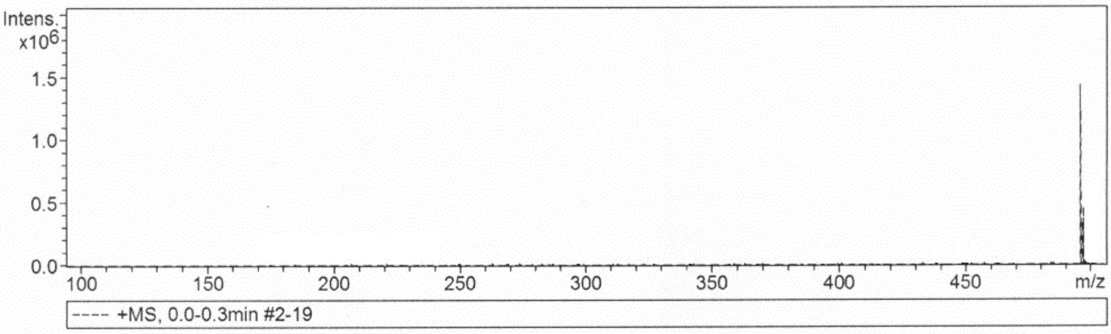

#	m/z	Res.	S/N	I	I %	FWHM
1	496.0924	25200	14496.2	1438795	100.0	0.0197
2	498.0897	19270	6287.3	624141	43.4	0.0258

15. NMR spectra and HR MS of 2-(4-(8-chloro quino[3,2-b]benzo[1,4]thiazin-6-yl)butyl)isoindoline-1,3-dione (**13**).

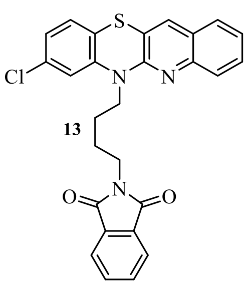

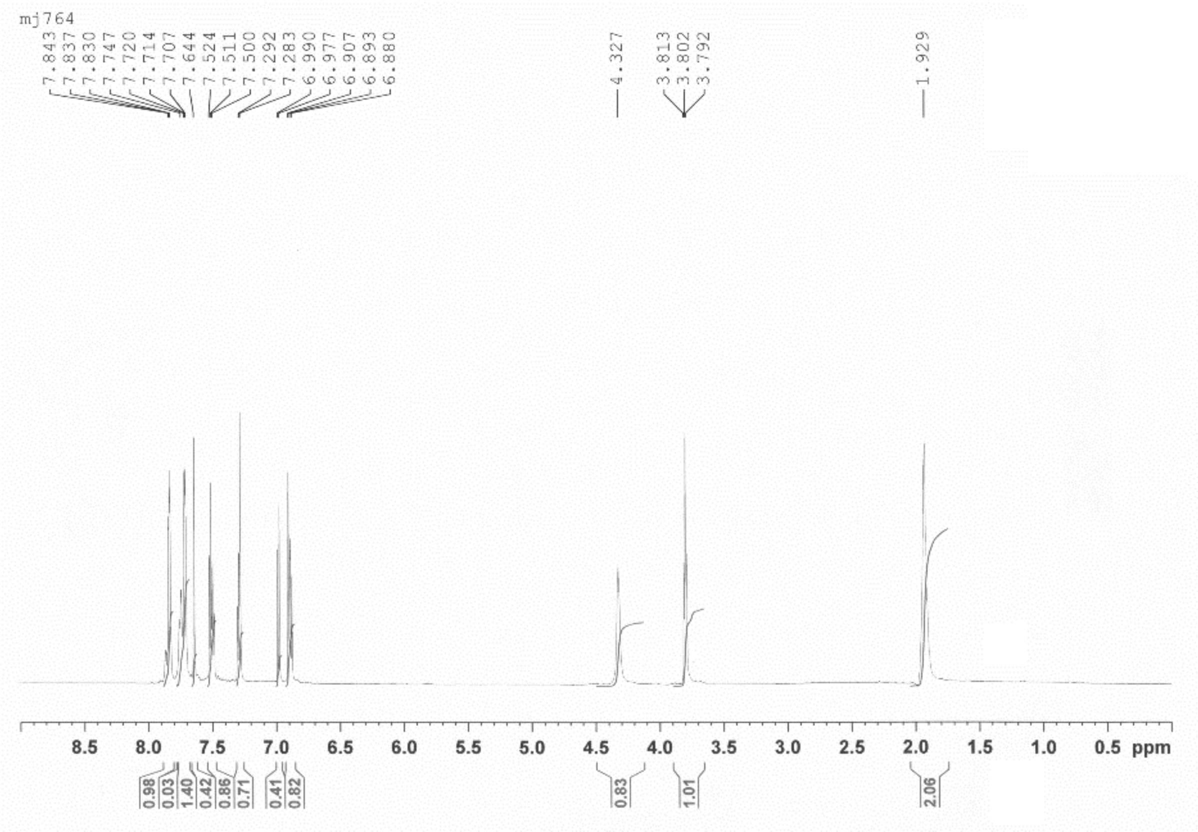

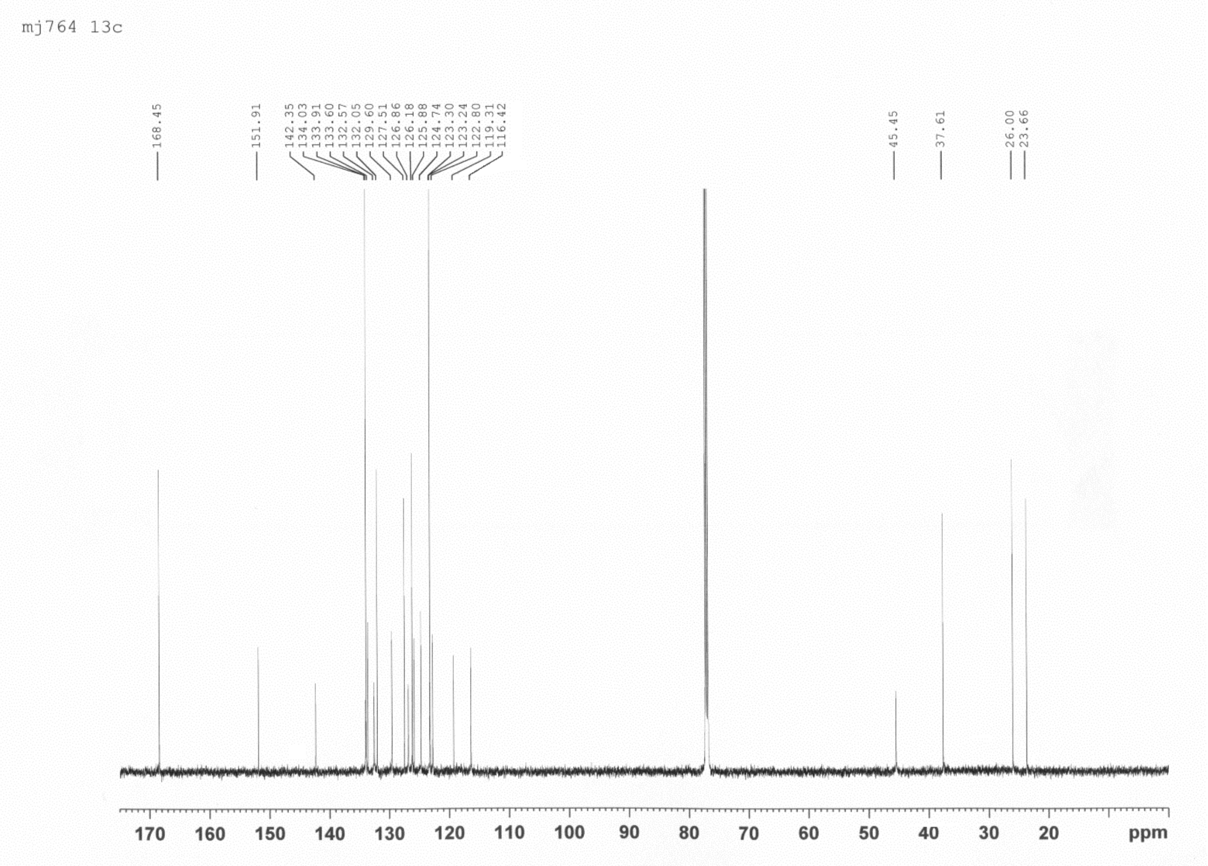

Acquisition Parameter

Source Type	ESI	Ion Polarity	Positive	Set Nebulizer	0.3 Bar
Focus	Active	Set Capillary	4000 V	Set Dry Heater	200 °C
Scan Begin	100 m/z	Set End Plate Offset	-500 V	Set Dry Gas	3.0 l/min
Scan End	1000 m/z	Set Charging Voltage	2000 V	Set Divert Valve	Source
		Set Corona	0 nA	Set APCI Heater	0 °C

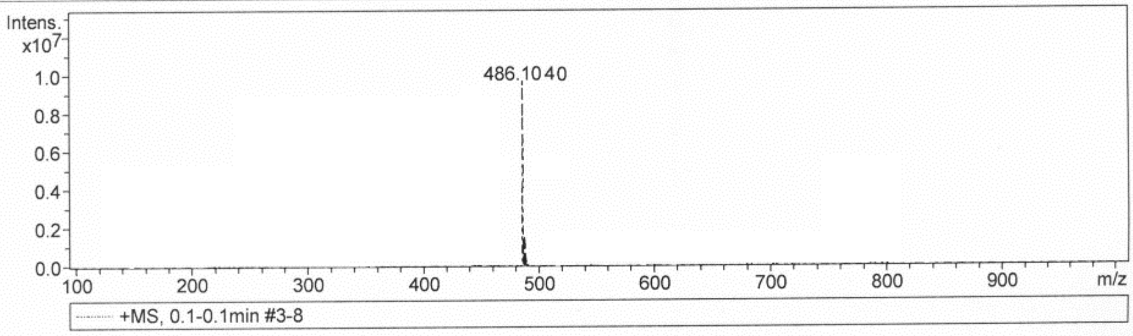

#	m/z	Res.	S/N	I	I %	FWHM
1	486.1040	41233	6045.6	4573534	100.0	0.0118

16. NMR spectra and HR MS of 4-(8-chloroquino[3,2-b]benzo[1,4]thiazin-6-yl)butan-1-amine (**15**).

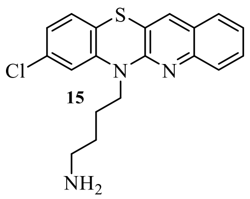

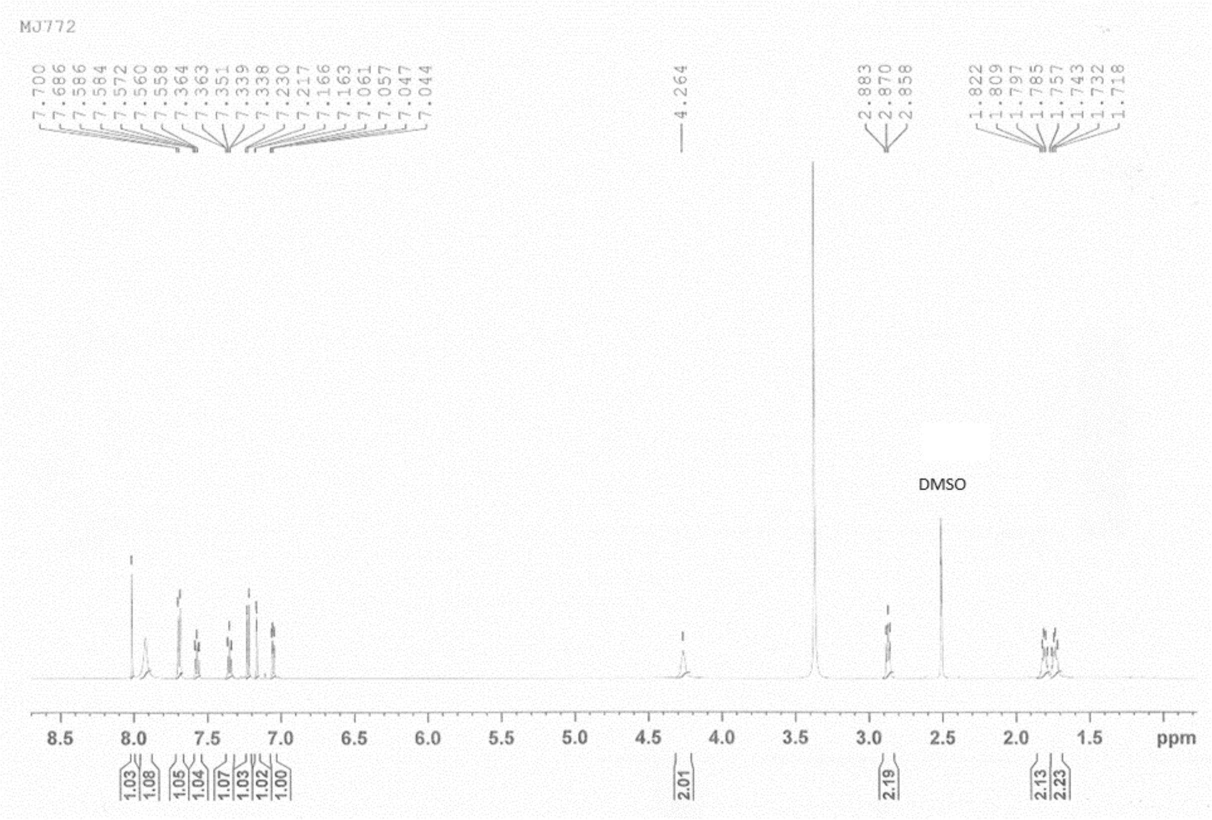

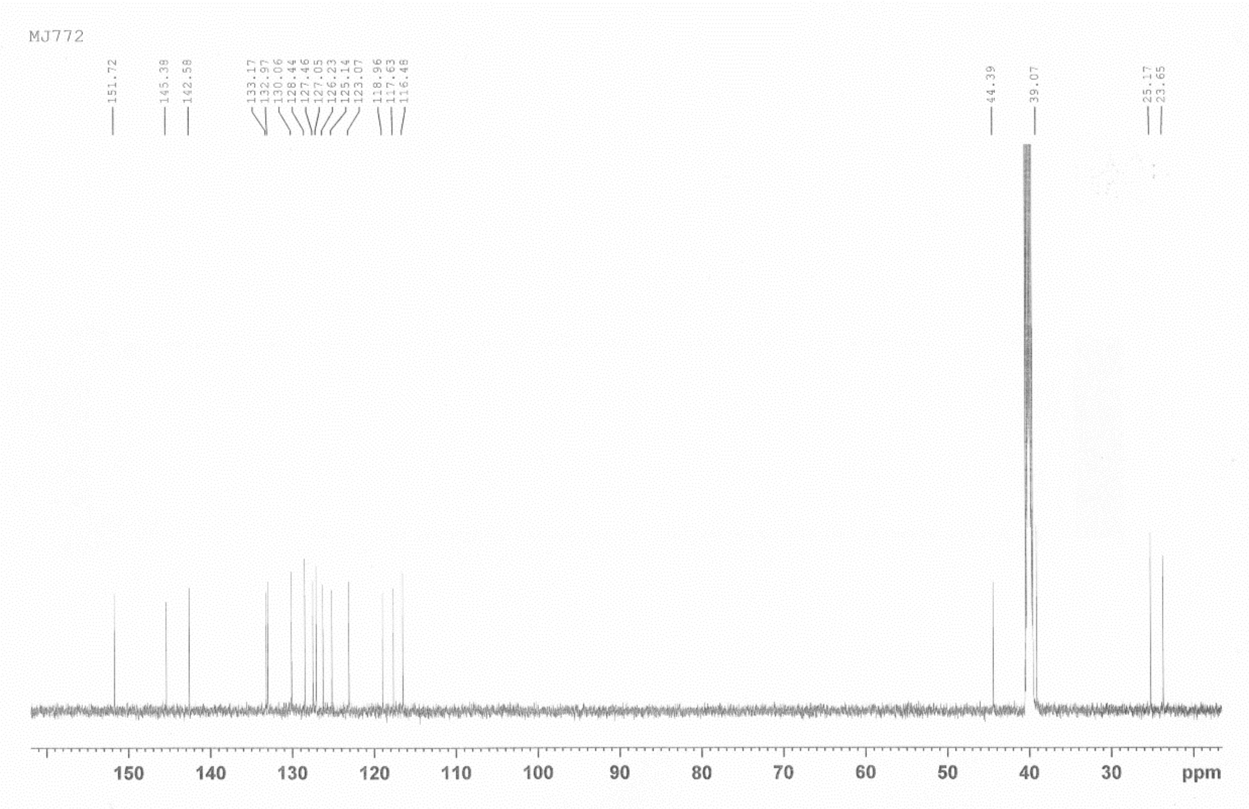

Acquisition Parameter

Source Type	ESI	Ion Polarity	Positive	Set Nebulizer	0.3 Bar
Focus	Not active	Set Capillary	4000 V	Set Dry Heater	200 °C
Scan Begin	100 m/z	Set End Plate Offset	-500 V	Set Dry Gas	3.0 l/min
Scan End	1000 m/z	Set Charging Voltage	2000 V	Set Divert Valve	Source
		Set Corona	0 nA	Set APCI Heater	0 °C

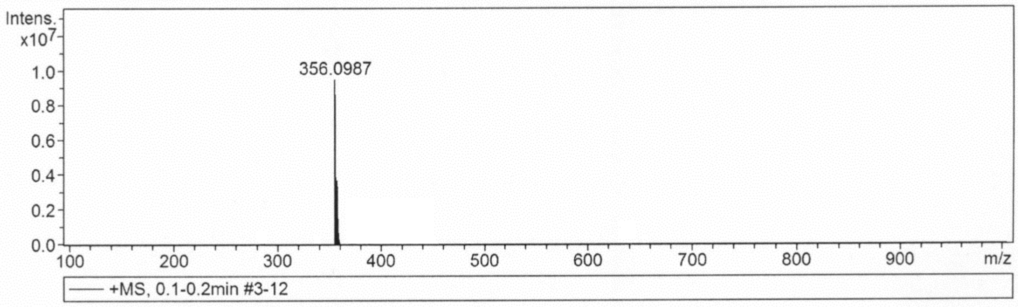

#	m/z	Res.	S/N	I	I %	FWHM
1	356.0987	12588	110224.2	9527740	100.0	0.0283
2	358.0954	13358	42185.6	3668580	38.5	0.0268

17. NMR spectra and HR MS of N-(4-(8-chloroquino[3,2-b]benzo[1,4]thiazin-6-yl)butyl)acetamide (**17**).

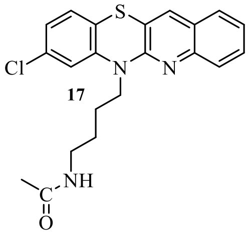

17

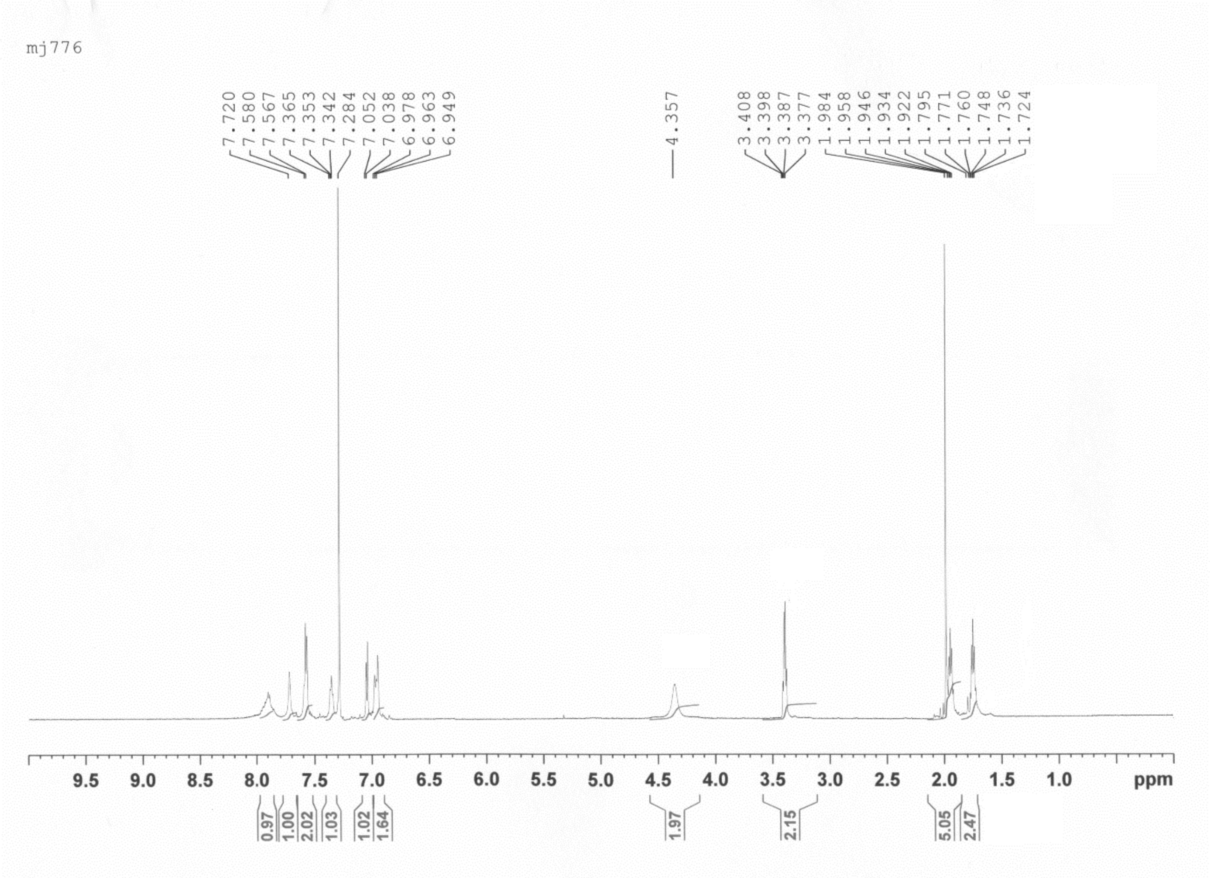

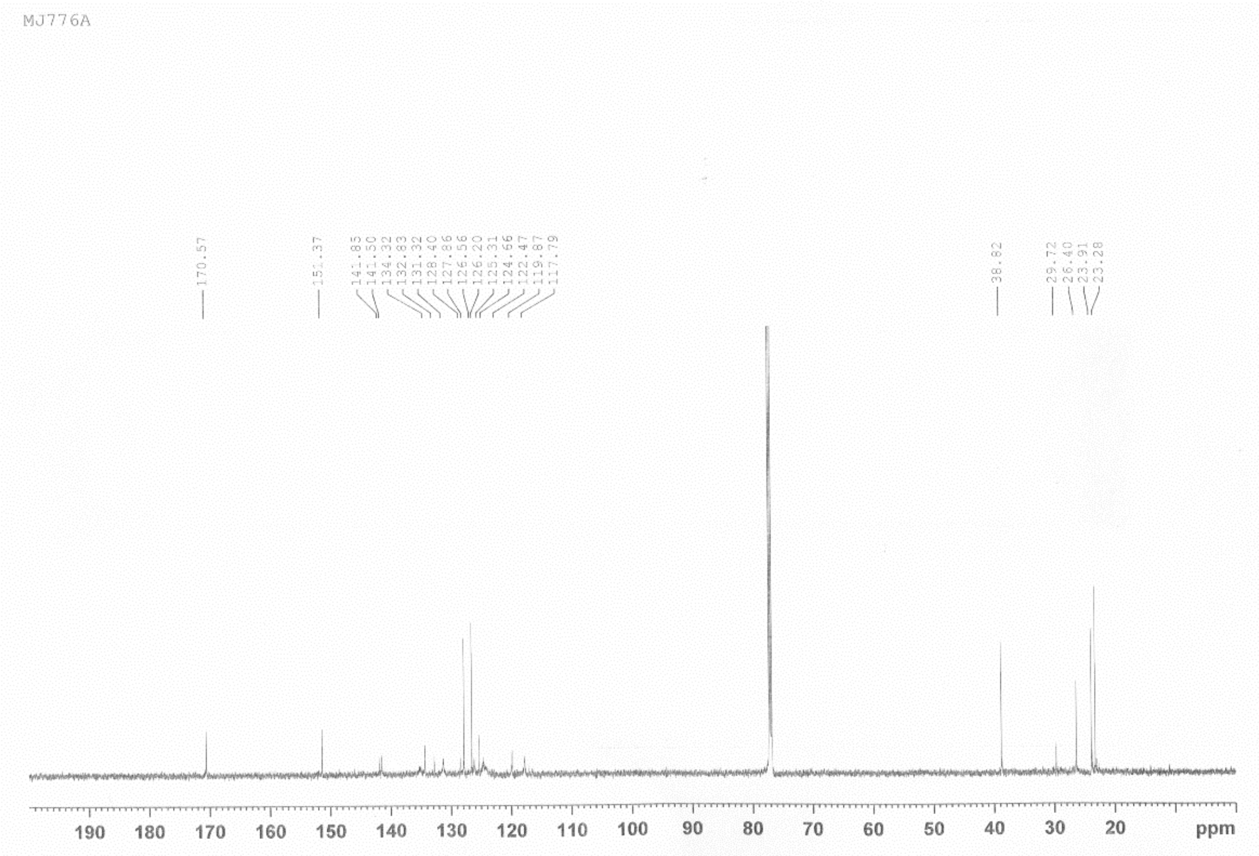

Acquisition Parameter

Source Type	ESI	Ion Polarity	Positive	Set Nebulizer	0.3 Bar
Focus	Not active	Set Capillary	4000 V	Set Dry Heater	200 °C
Scan Begin	100 m/z	Set End Plate Offset	-500 V	Set Dry Gas	3.0 l/min
Scan End	1000 m/z	Set Charging Voltage	2000 V	Set Divert Valve	Source
		Set Corona	0 nA	Set APCI Heater	0 °C

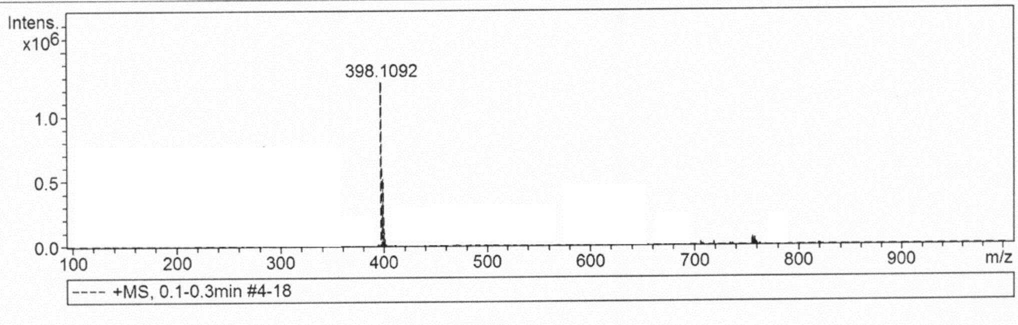

#	m/z	Res.	S/N	I	I %	FWHM
1	398.1092	13619	9225.4	1268062	100.0	0.0292
2	400.1063	13496	3545.4	490455	38.7	0.0296

18. NMR spectra and HR MS of ethyl(4-(8-chloro-quino[3,2-b]benzo[1,4]thiazin-6-yl)butyl)carbamate (**19**).

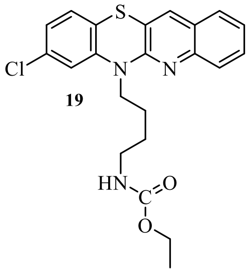

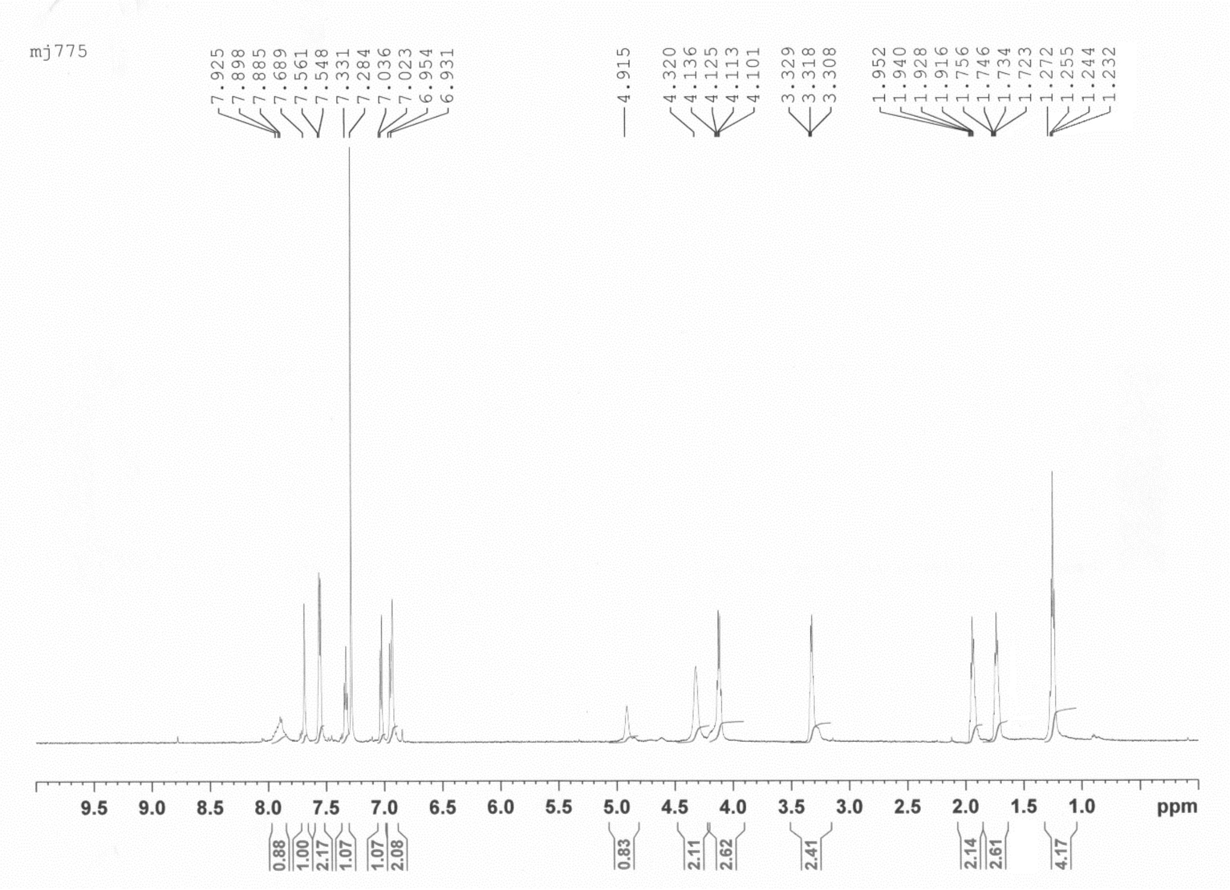

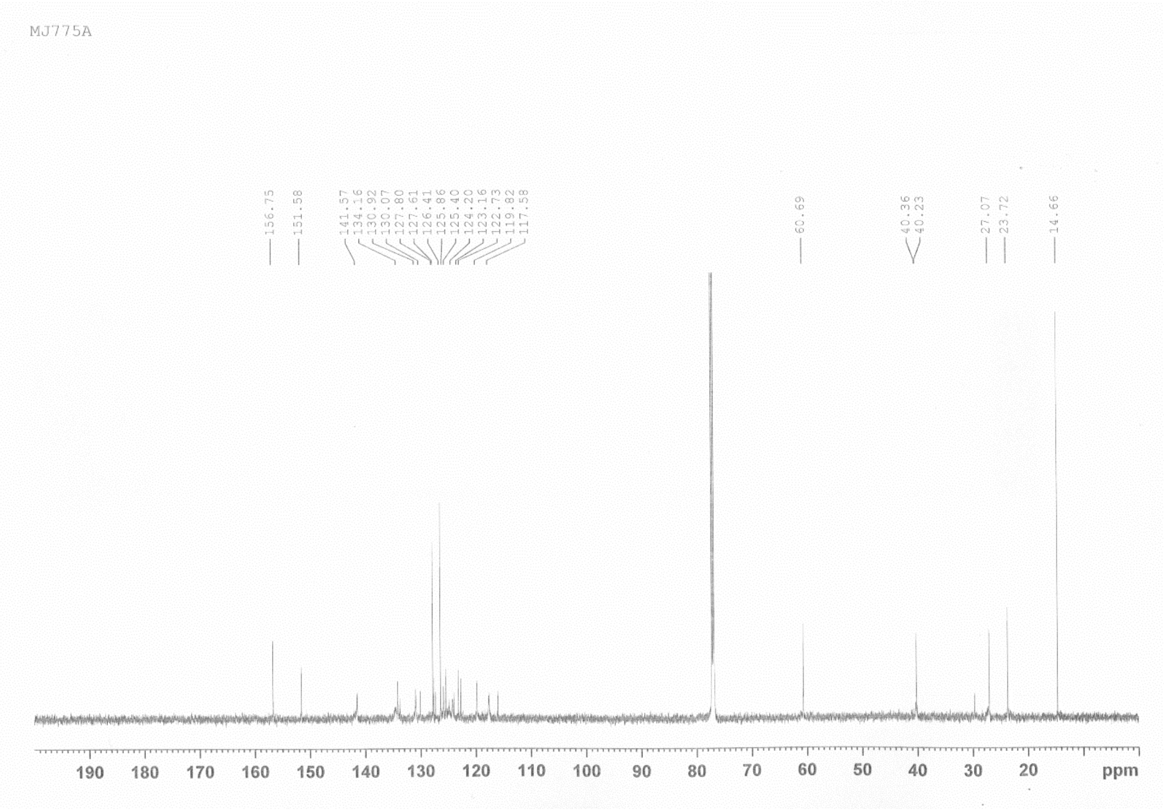

Acquisition Parameter

Source Type	ESI	Ion Polarity	Positive	Set Nebulizer	0.3 Bar
Focus	Not active	Set Capillary	4000 V	Set Dry Heater	200 °C
Scan Begin	100 m/z	Set End Plate Offset	-500 V	Set Dry Gas	3.0 l/min
Scan End	1000 m/z	Set Charging Voltage	2000 V	Set Divert Valve	Source
		Set Corona	0 nA	Set APCI Heater	0 °C

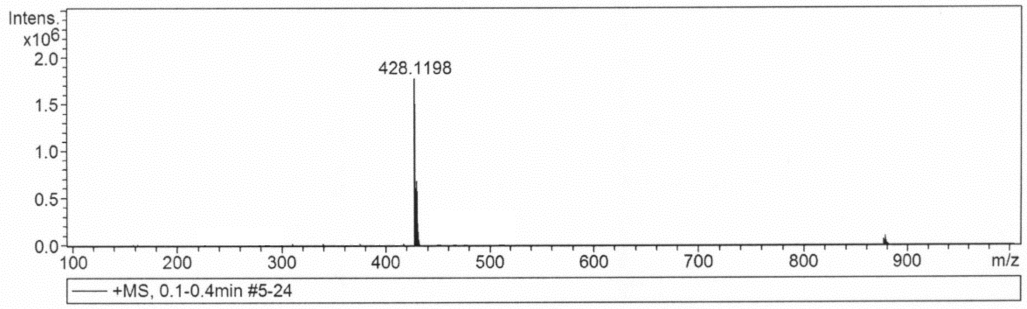

#	m/z	Res.	S/N	I	I %	FWHM
1	428.1198	13986	21640.4	1773247	100.0	0.0306
2	430.1171	13745	8352.5	686719	38.7	0.0313

19. NMR spectra and HR MS of 1-(4-(8-chloroquino[3,2-b]benzo[1,4]thiazin-6-yl)butyl)-3-(2-chloroethyl)urea (**21**).

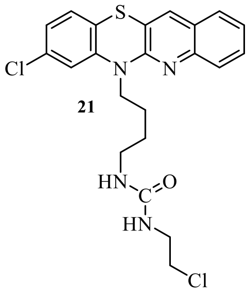

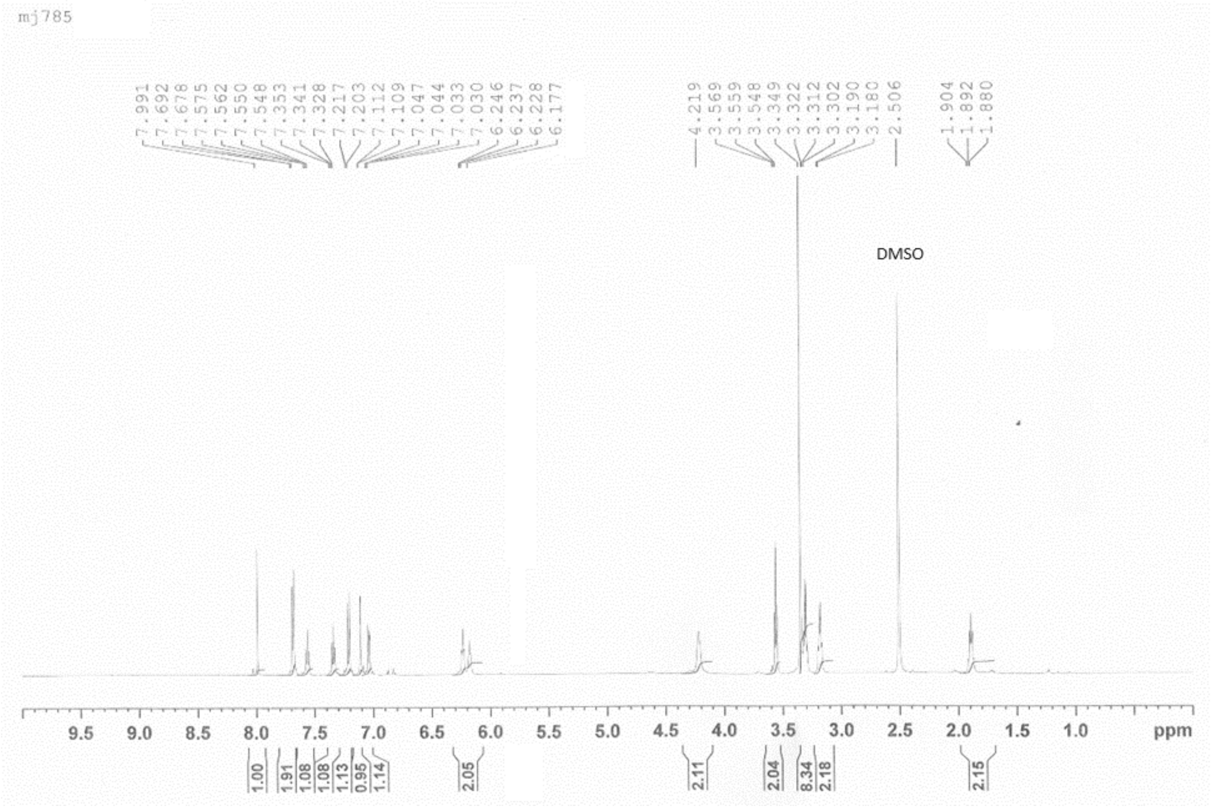

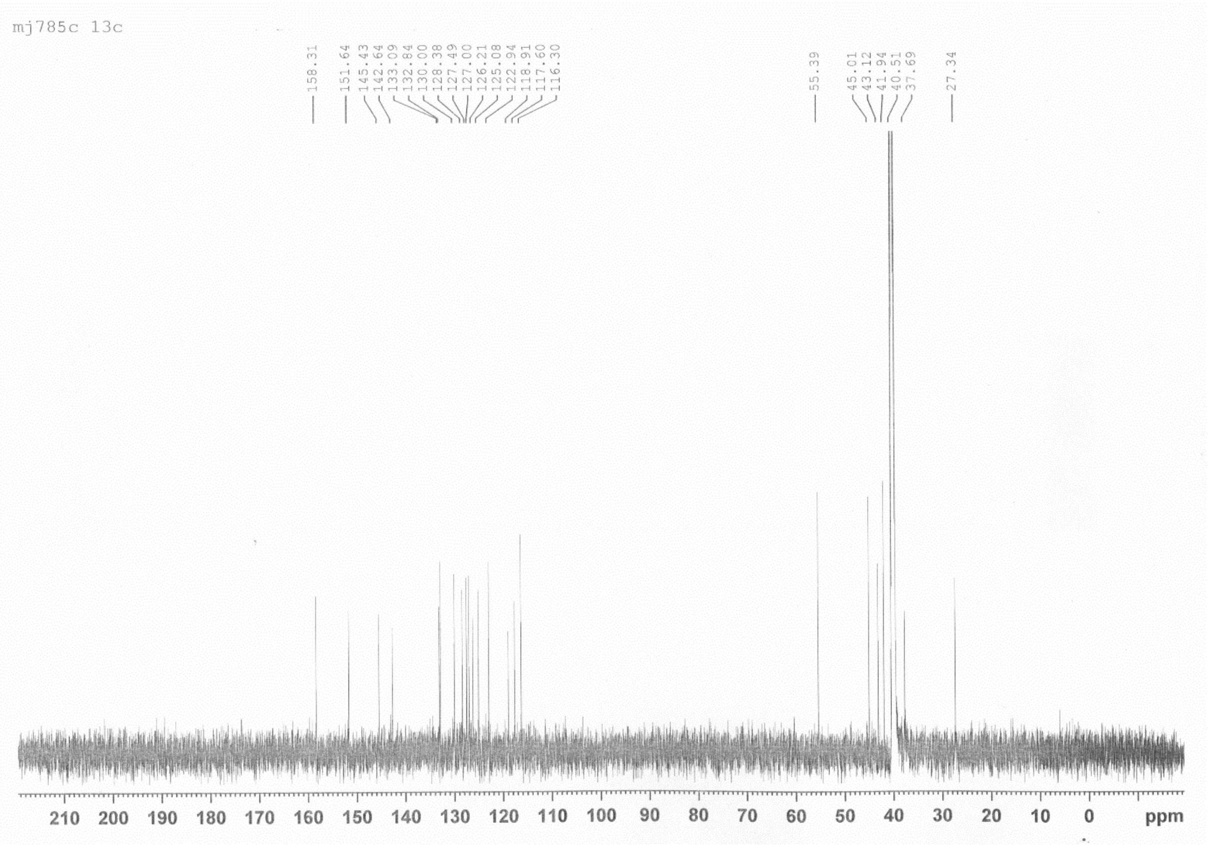

Acquisition Parameter

Source Type	ESI	Ion Polarity	Positive	Set Nebulizer	0.3 Bar
Focus	Active	Set Capillary	4000 V	Set Dry Heater	200 °C
Scan Begin	100 m/z	Set End Plate Offset	-500 V	Set Dry Gas	3.0 l/min
Scan End	1000 m/z	Set Charging Voltage	2000 V	Set Divert Valve	Source
		Set Corona	0 nA	Set APCI Heater	0 °C

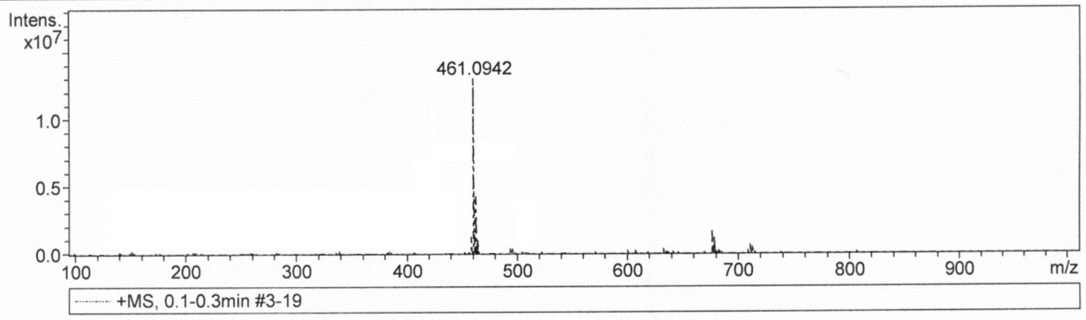

#	m/z	Res.	S/N	I	I %	FWHM
1	461.0942	43730	5526.7	6555349	100.0	0.0105
2	463.0719	41548	3633.5	4332482	66.1	0.0111

20. NMR spectra and HR MS of N-(4-(8-chloroquino[3,2-b]benzo[1,4]thiazin-6-yl)butyl)methanesulfonamide (**23**).

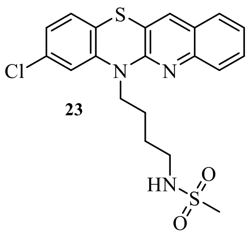

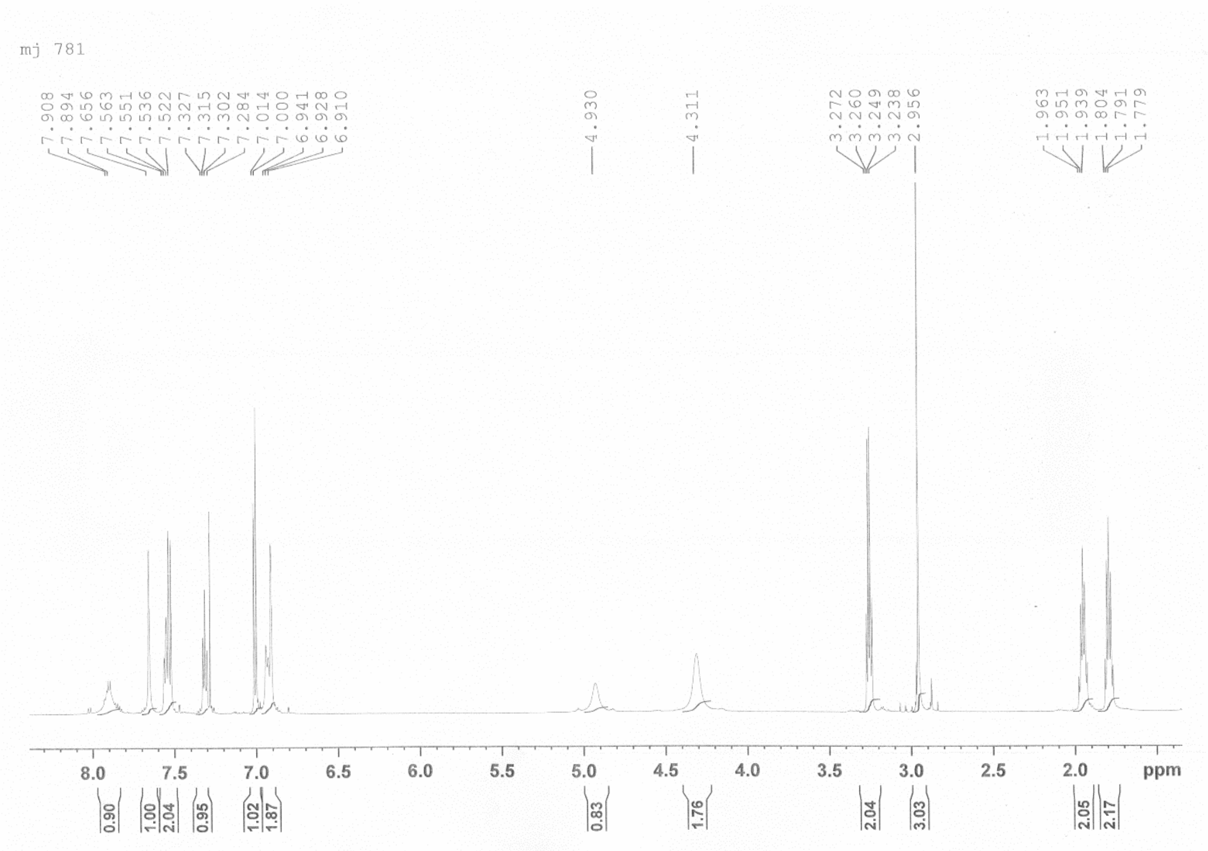

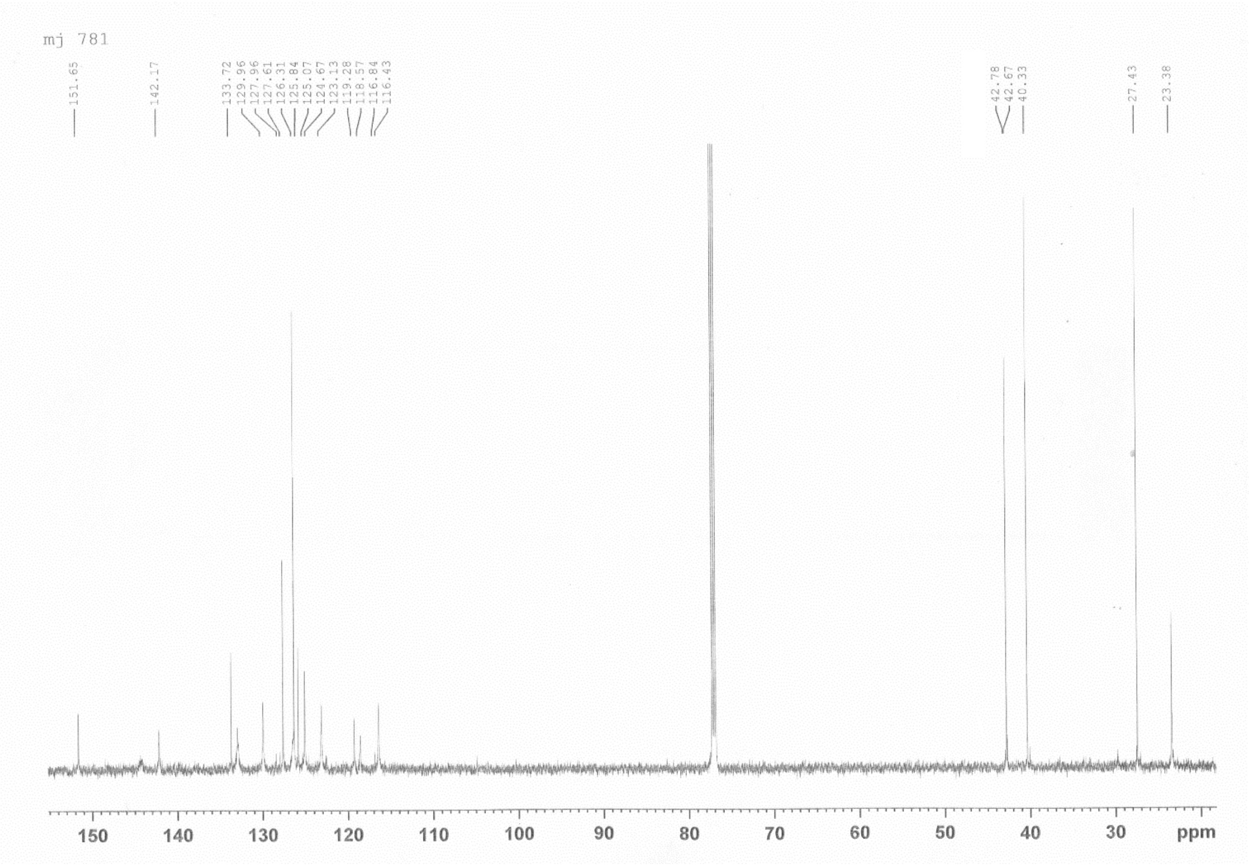

Acquisition Parameter

Source Type	ESI	Ion Polarity	Positive	Set Nebulizer	0.3 Bar
Focus	Not active	Set Capillary	4000 V	Set Dry Heater	200 °C
Scan Begin	100 m/z	Set End Plate Offset	-500 V	Set Dry Gas	3.0 l/min
Scan End	1000 m/z	Set Charging Voltage	2000 V	Set Divert Valve	Source
		Set Corona	0 nA	Set APCI Heater	0 °C

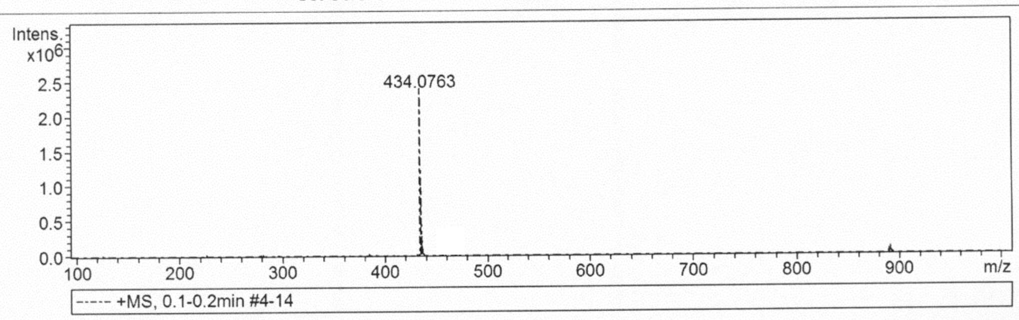

#	m/z	Res.	S/N	I	I %	FWHM
1	434.0763	13896	36384.3	2348999	100.0	0.0312
2	436.0732	13652	15313.9	993562	42.3	0.0319

21. NMR spectra and HR MS of N-(4-(8-chloroquino[3,2-b]benzo[1,4]thiazin-6-yl)butyl)-4-methylbenzenesulfonamide (**25**).

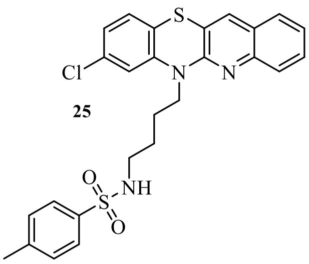

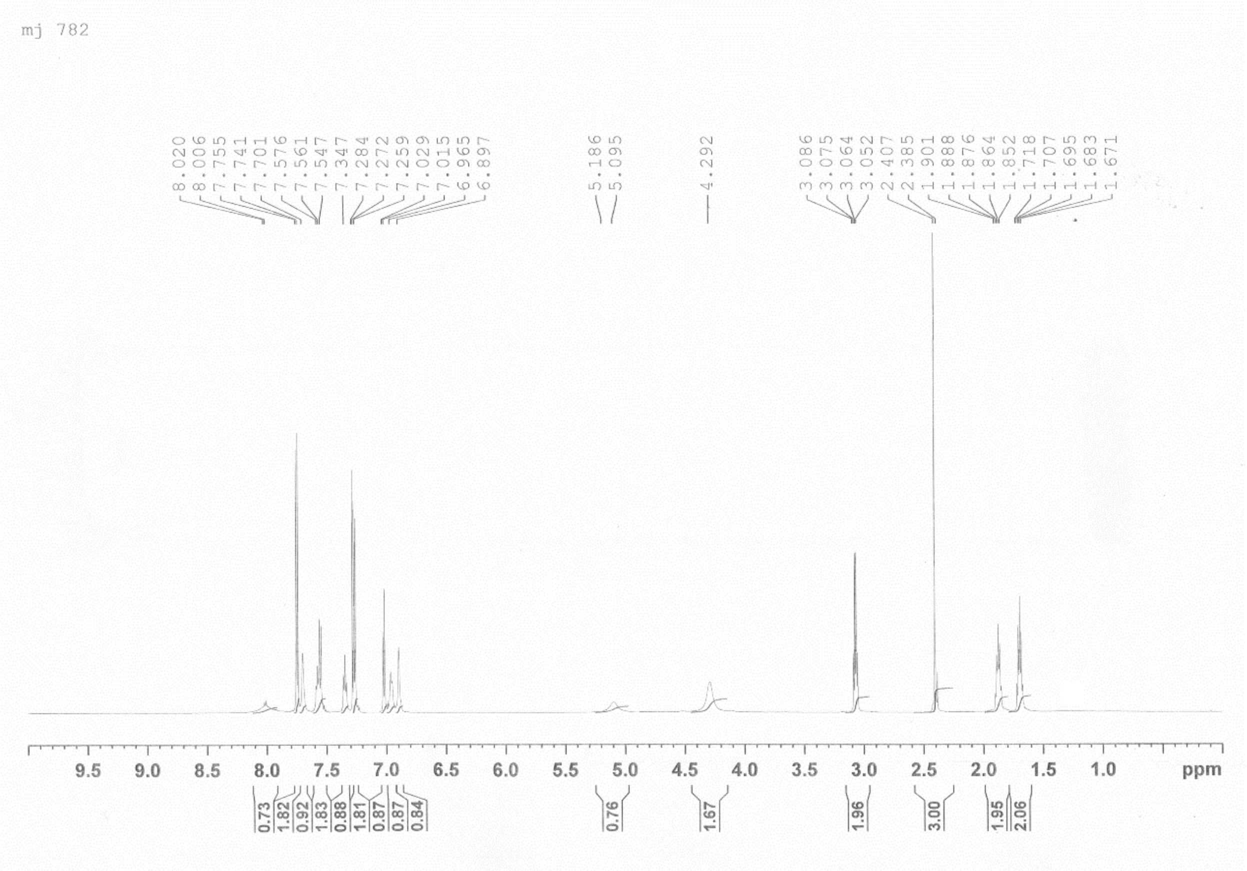

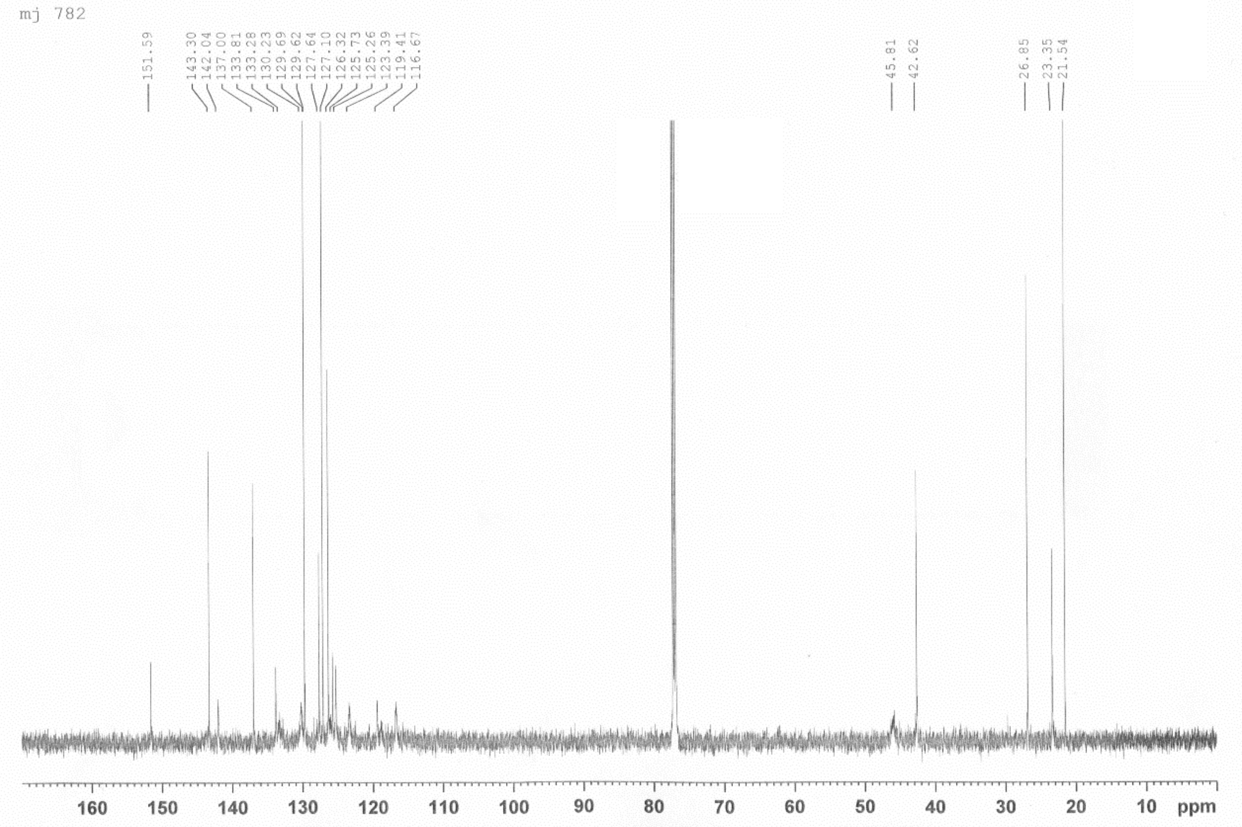

Acquisition Parameter

Source Type	ESI	Ion Polarity	Positive	Set Nebulizer	0.3 Bar
Focus	Not active	Set Capillary	4000 V	Set Dry Heater	200 °C
Scan Begin	100 m/z	Set End Plate Offset	-500 V	Set Dry Gas	3.0 l/min
Scan End	1000 m/z	Set Charging Voltage	2000 V	Set Divert Valve	Source
		Set Corona	0 nA	Set APCI Heater	0 °C

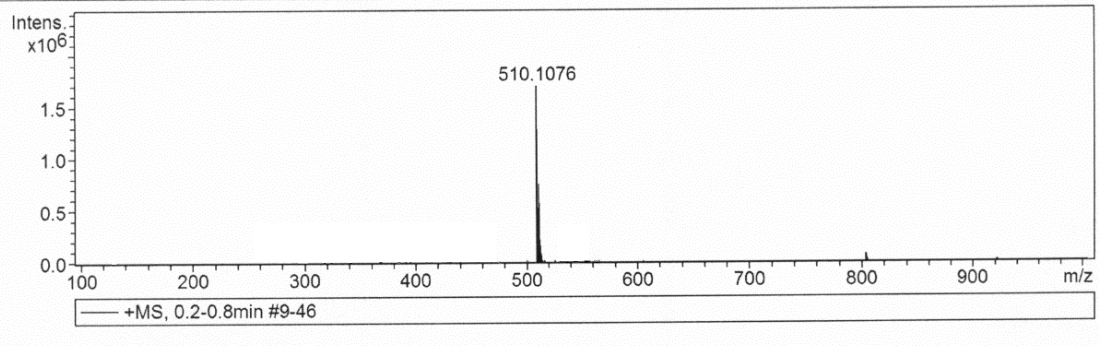

#	m/z	Res.	S/N	I	I %	FWHM
1	510.1076	13939	14844.9	1708201	100.0	0.0366
2	512.1048	13680	6597.7	761488	44.6	0.0374

22. NMR spectra and HR MS of 8-chloro-6-((1-phenyl-1*H*-1,2,3-triazol-4-yl)methyl)-quino[3,2-b]benzo[1,4]thiazine (**27**).

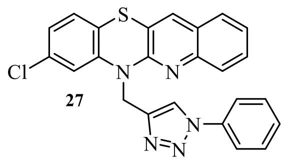

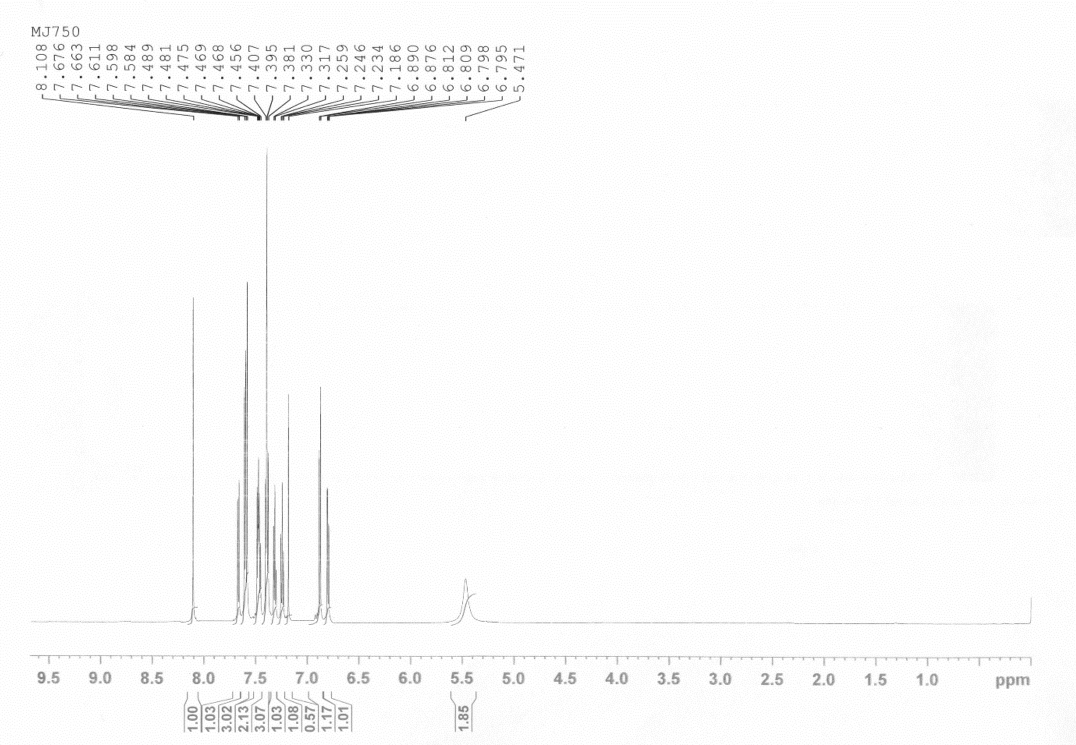

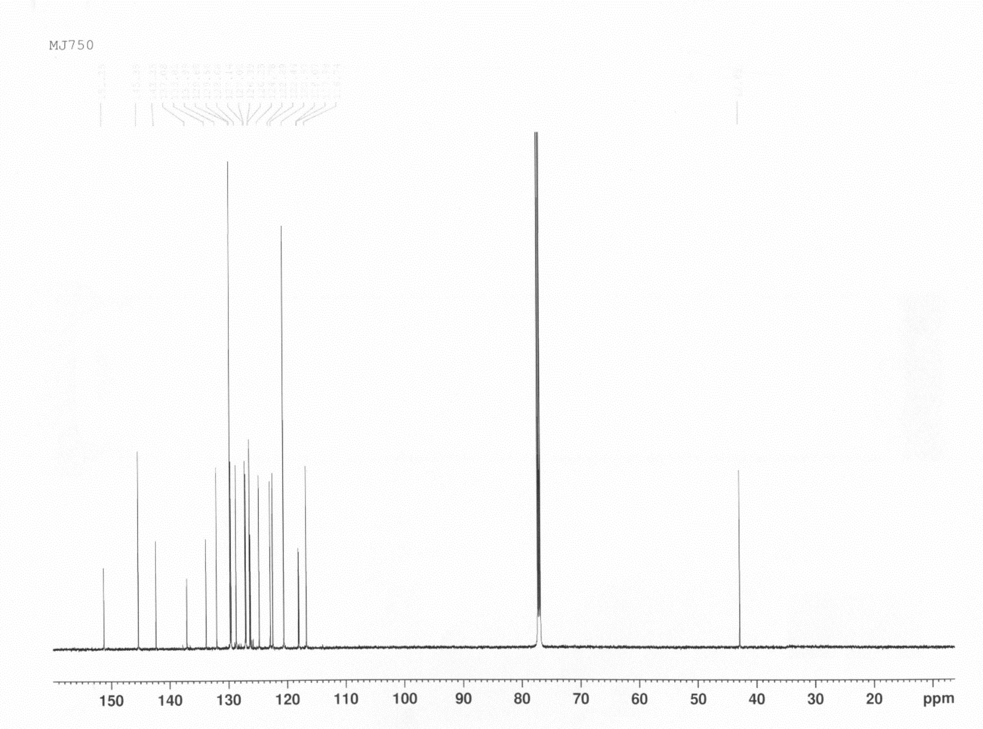

Acquisition Parameter

Source Type	ESI	Ion Polarity	Positive	Set Nebulizer	0.3 Bar
Focus	Active	Set Capillary	4000 V	Set Dry Heater	240 °C
Scan Begin	100 m/z	Set End Plate Offset	-500 V	Set Dry Gas	4.0 l/min
Scan End	1000 m/z	Set Charging Voltage	2000 V	Set Divert Valve	Source
		Set Corona	0 nA	Set APCI Heater	0 °C

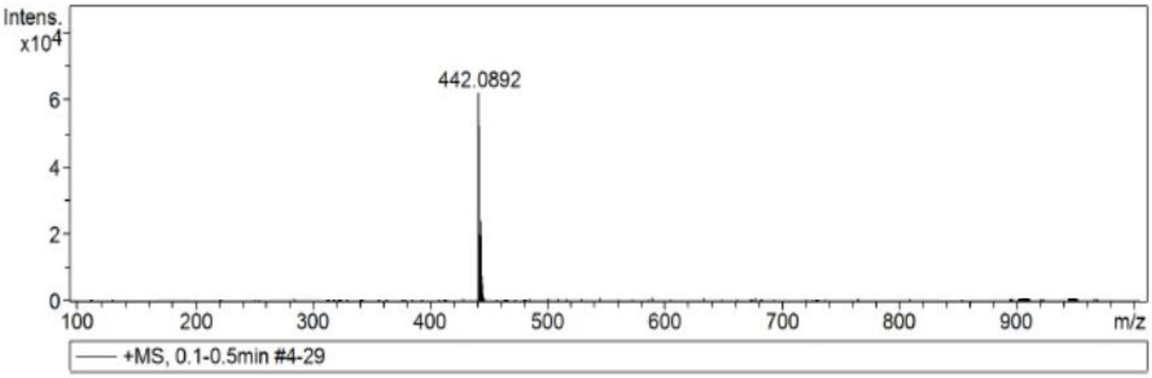

#	m/z	Res.	S/N	I	I %	FWHM
1	442.0892	22688	4657.9	61769	100.0	0.0195
2	444.0868	21031	1818.3	24166	39.1	0.0211

23. NMR spectra and HR MS of 8-chloro-6-((1-(4-(trifluoromethyl)phenyl)-1*H*-1,2,3-triazol-4-yl)methyl)-quino[3,2-b]benzo[1,4]thiazine (**28**).

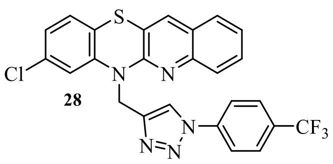

28

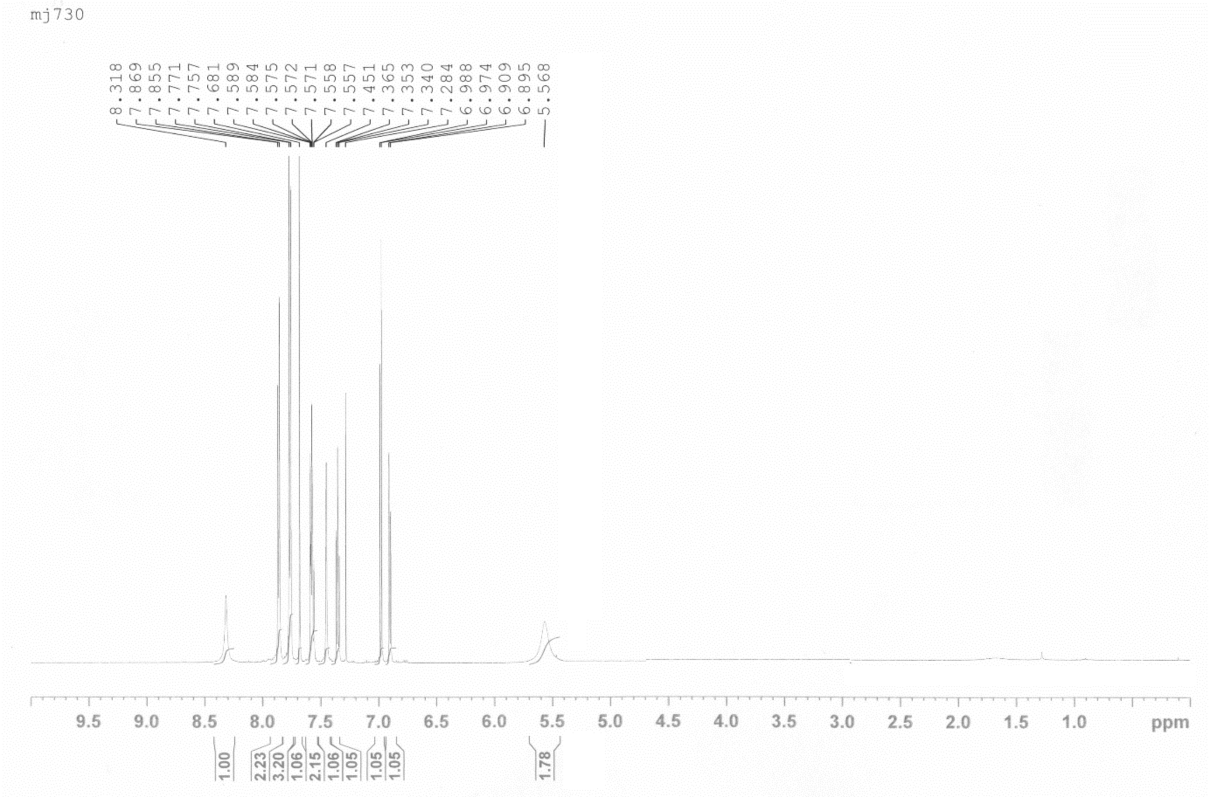

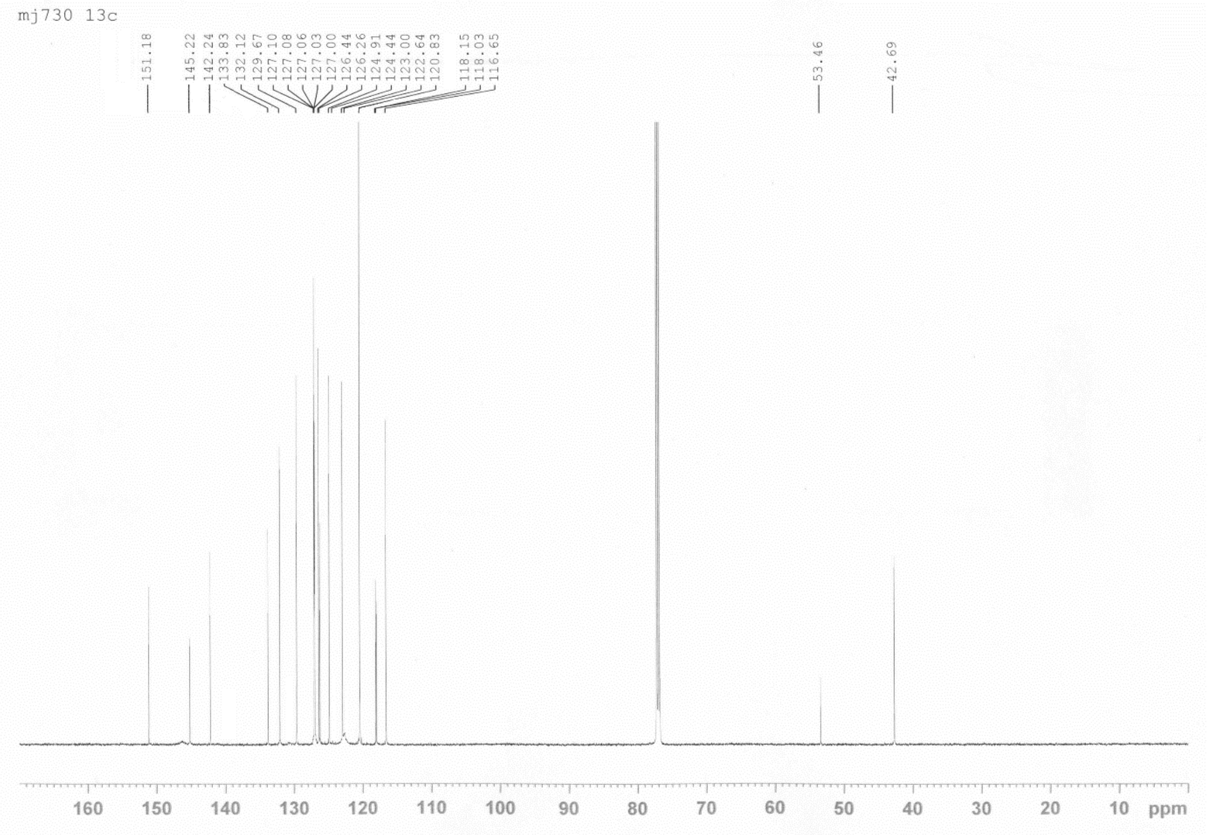

Acquisition Parameter

Source Type	ESI	Ion Polarity	Positive	Set Nebulizer	0.3 Bar
Focus	Active	Set Capillary	4000 V	Set Dry Heater	240 °C
Scan Begin	100 m/z	Set End Plate Offset	-500 V	Set Dry Gas	4.0 l/min
Scan End	1000 m/z	Set Charging Voltage	2000 V	Set Divert Valve	Source
		Set Corona	0 nA	Set APCI Heater	0 °C

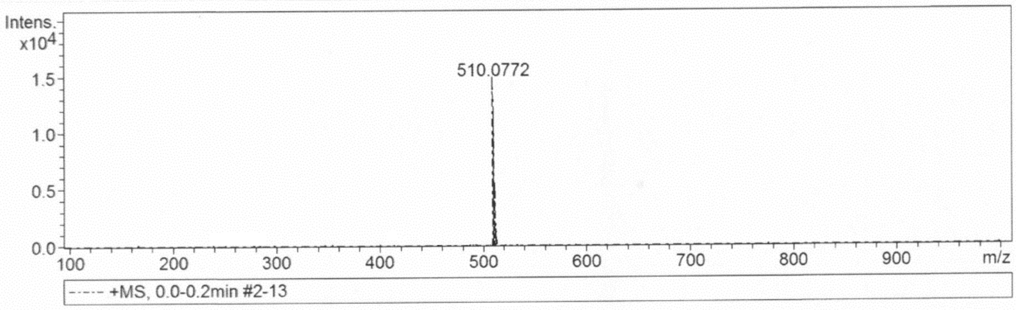

#	m/z	Res.	S/N	I	I %	FWHM
1	510.0772	22849	1149.1	14607	100.0	0.0223

24. NMR spectra and HR MS of 6-((1-benzyl-1*H*-1,2,3-triazol-4-yl)methyl)-8-chloroquino[3,2-b]benzo[1,4]thiazine (**29**).

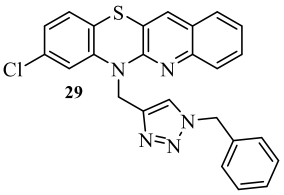

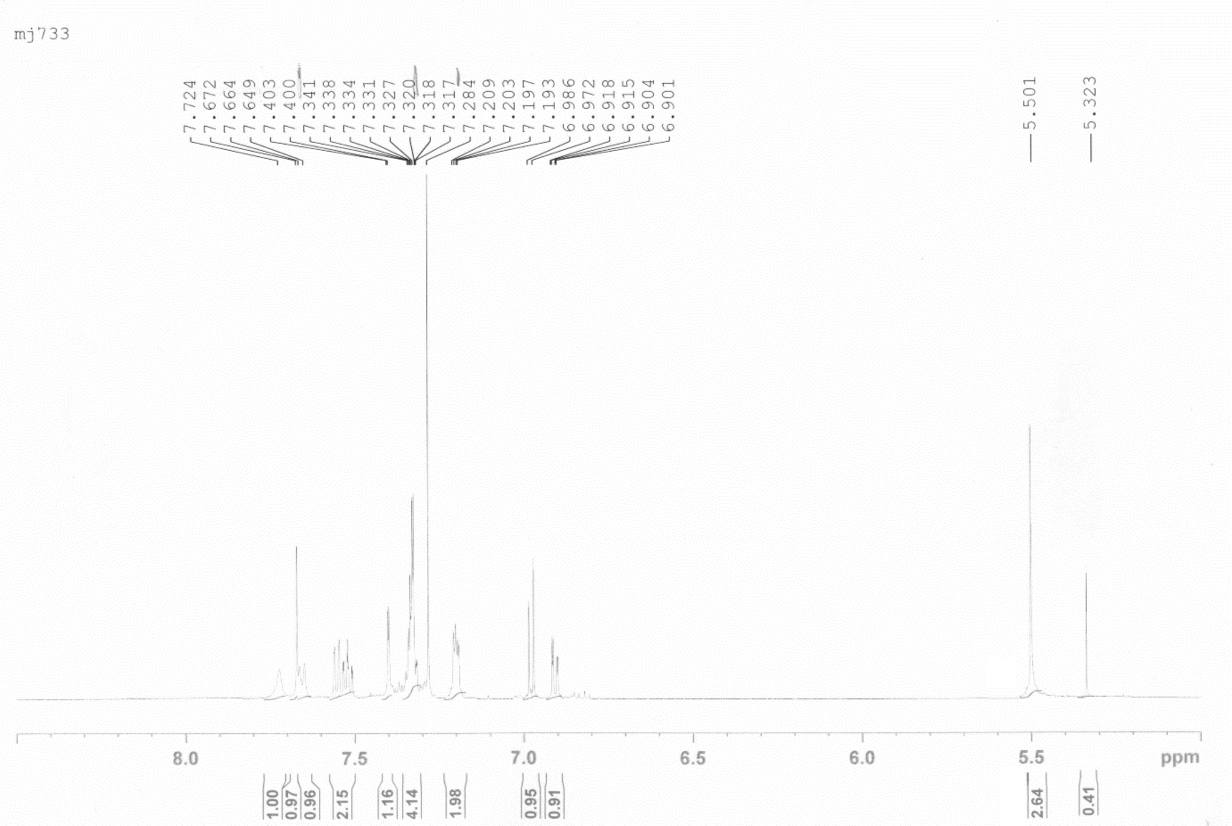

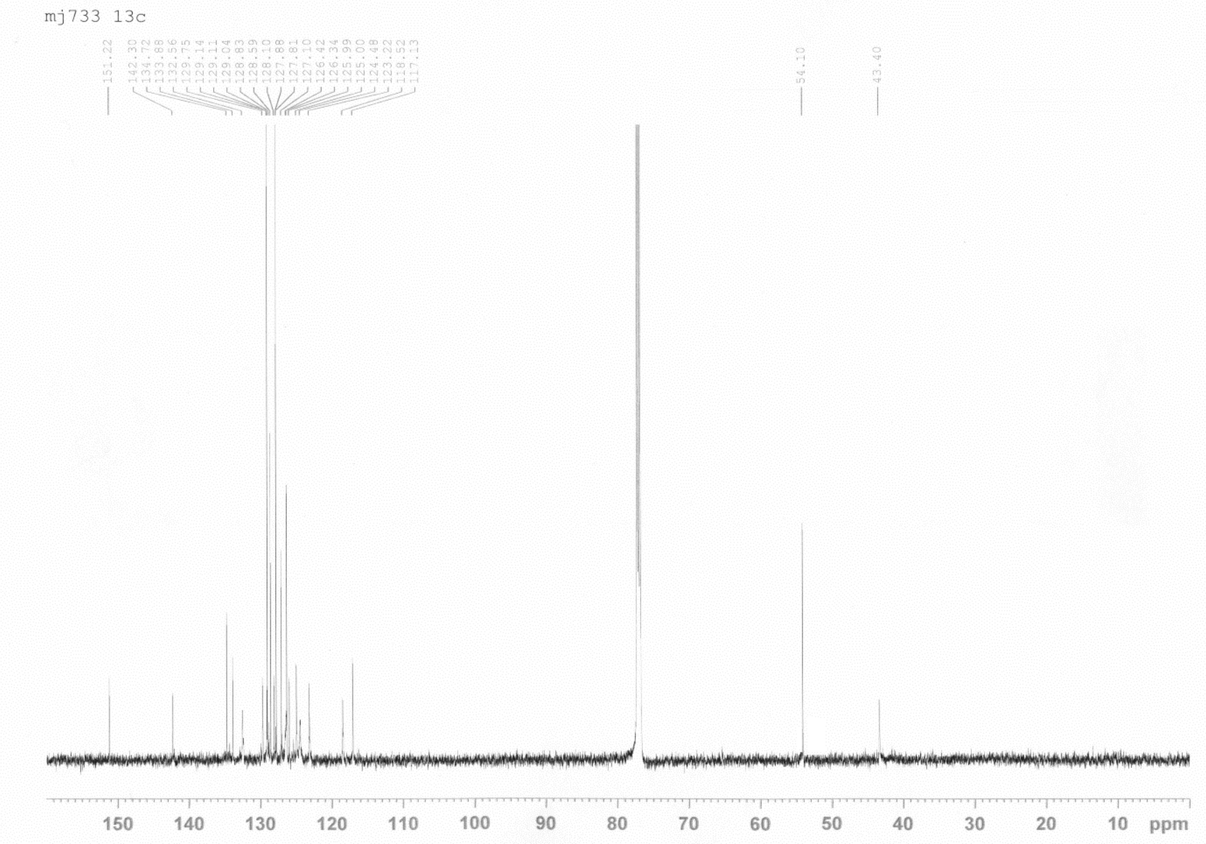

Acquisition Parameter

Source Type	ESI	Ion Polarity	Positive	Set Nebulizer	0.3 Bar
Focus	Active	Set Capillary	4000 V	Set Dry Heater	240 °C
Scan Begin	100 m/z	Set End Plate Offset	-500 V	Set Dry Gas	4.0 l/min
Scan End	1000 m/z	Set Charging Voltage	2000 V	Set Divert Valve	Source
		Set Corona	0 nA	Set APCI Heater	0 °C

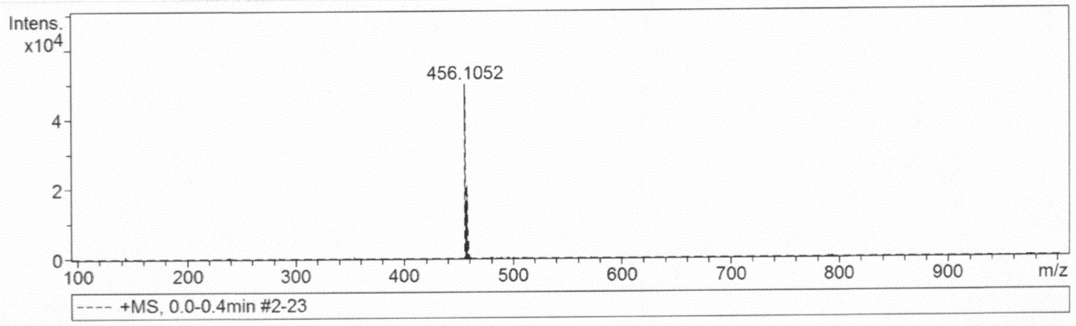

#	m/z	Res.	S/N	I	I %	FWHM
1	456.1052	22222	4335.4	49950	100.0	0.0205
2	458.1028	19880	1723.1	19866	39.8	0.0230

25. NMR spectra and HR MS of 8-chloro-6-((1-(4-fluorobenzyl)-1*H*-1,2,3-triazol-4-yl)methyl)-quino[3,2-b]benzo[1,4]thiazine (**30**).

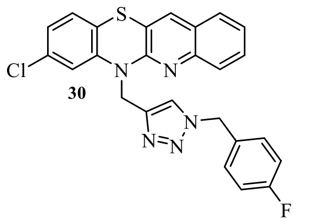

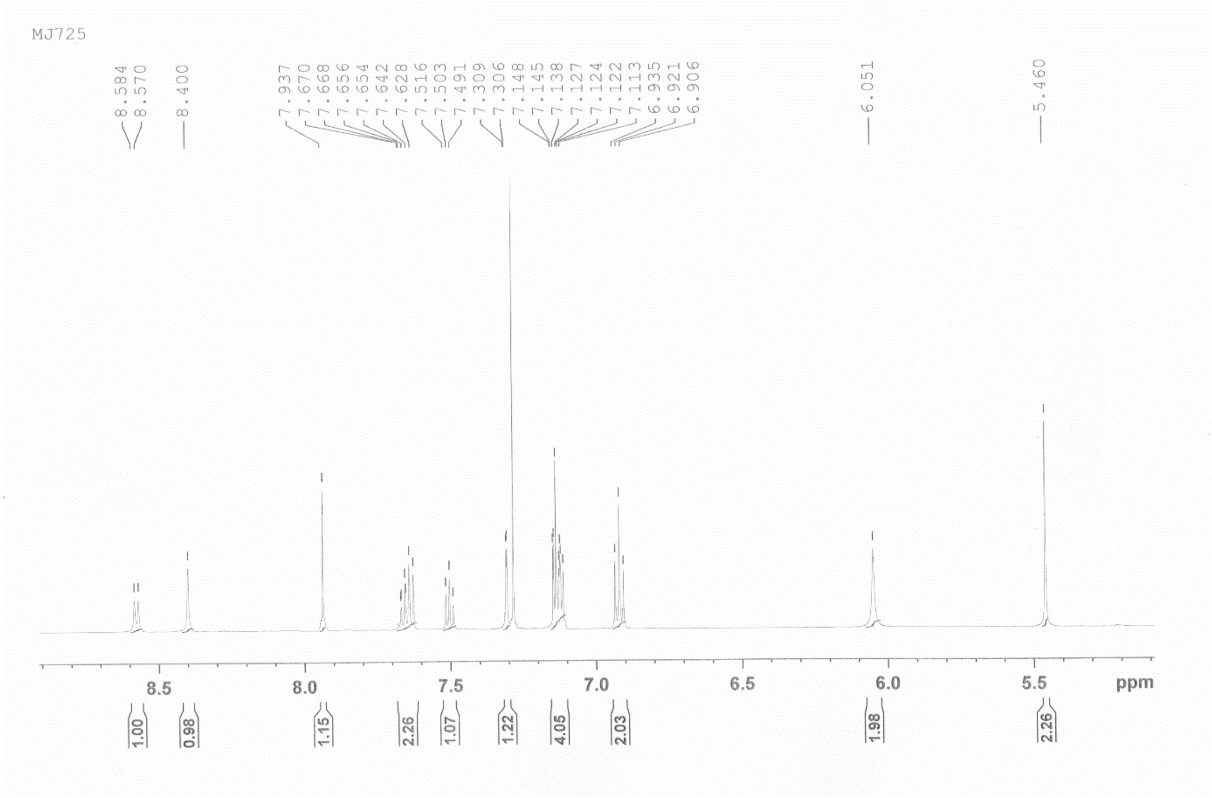

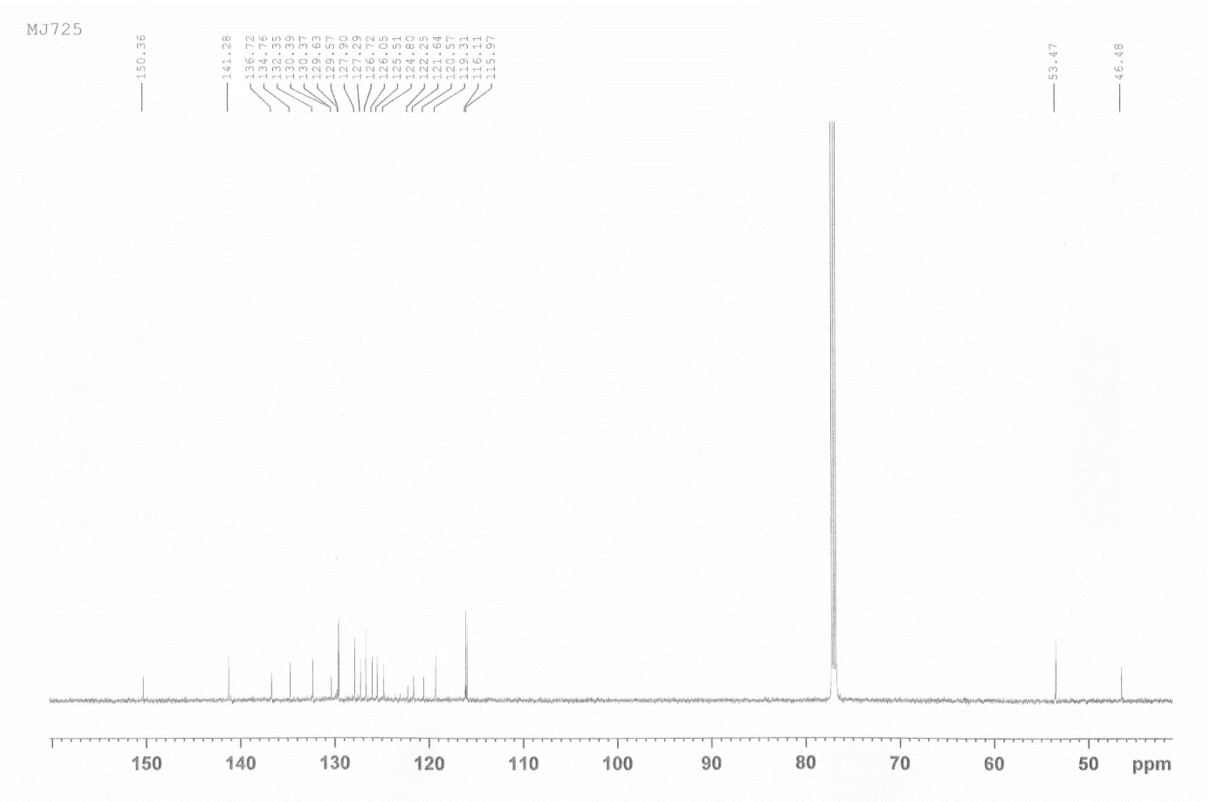

Acquisition Parameter

Source Type	ESI	Ion Polarity	Positive	Set Nebulizer	0.3 Bar
Focus	Active	Set Capillary	4000 V	Set Dry Heater	240 °C
Scan Begin	100 m/z	Set End Plate Offset	-500 V	Set Dry Gas	4.0 l/min
Scan End	1000 m/z	Set Charging Voltage	2000 V	Set Divert Valve	Source
		Set Corona	0 nA	Set APCI Heater	0 °C

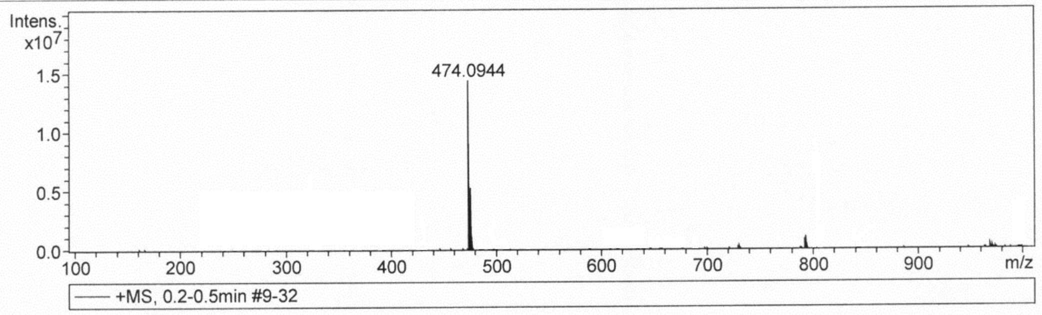

#	m/z	Res.	S/N	I	I %	FWHM
1	474.0944	48156	15401.2	14340540	100.0	0.0098
2	476.0916	46723	5583.8	5264247	36.7	0.0102

26. NMR spectra and HR MS of 8-chloro-6-((1-(4-chlorobenzyl)-1H-1,2,3-triazol-4-yl)methyl)-quino[3,2-b]benzo[1,4]thiazine (**31**).

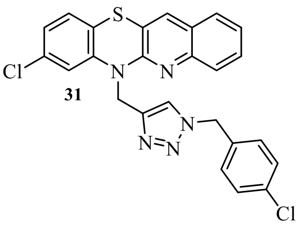

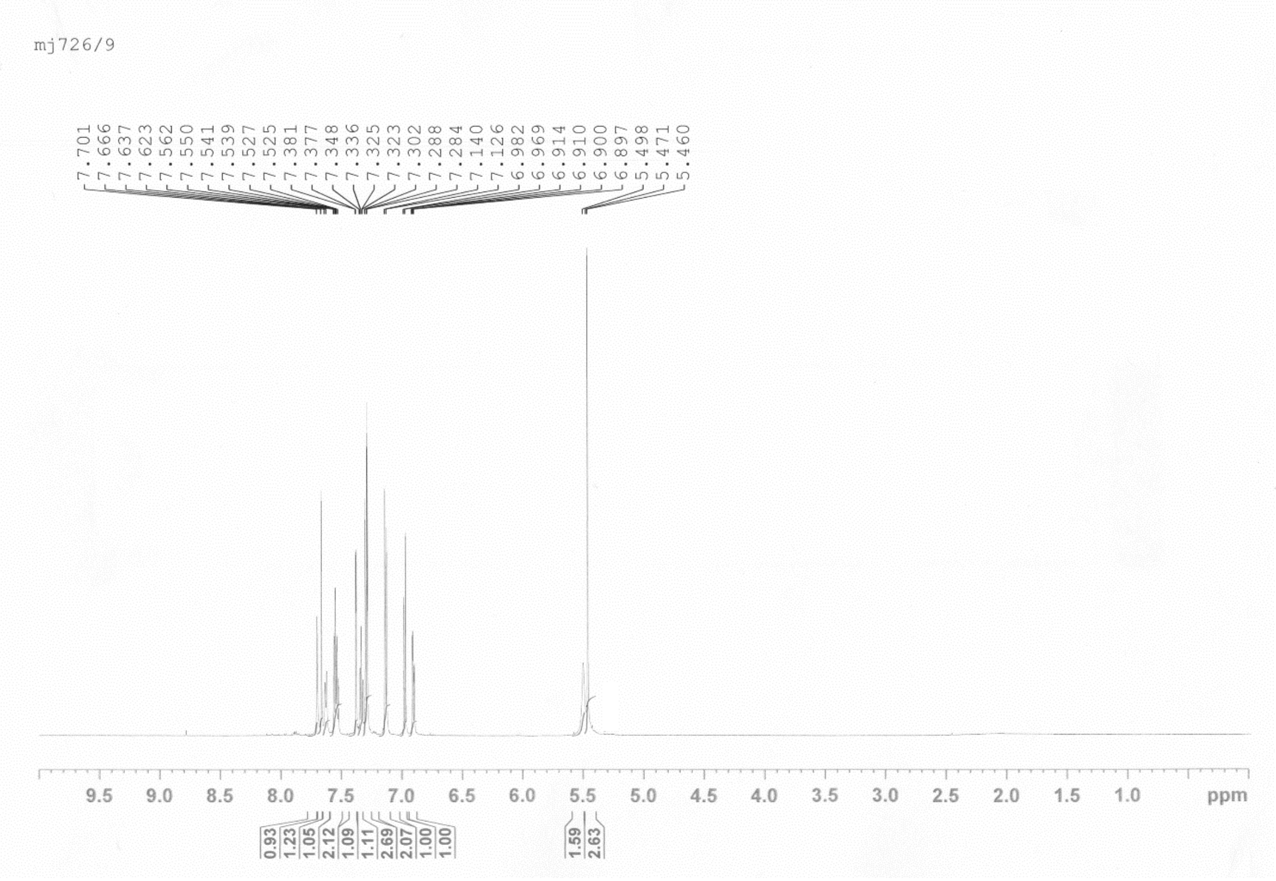

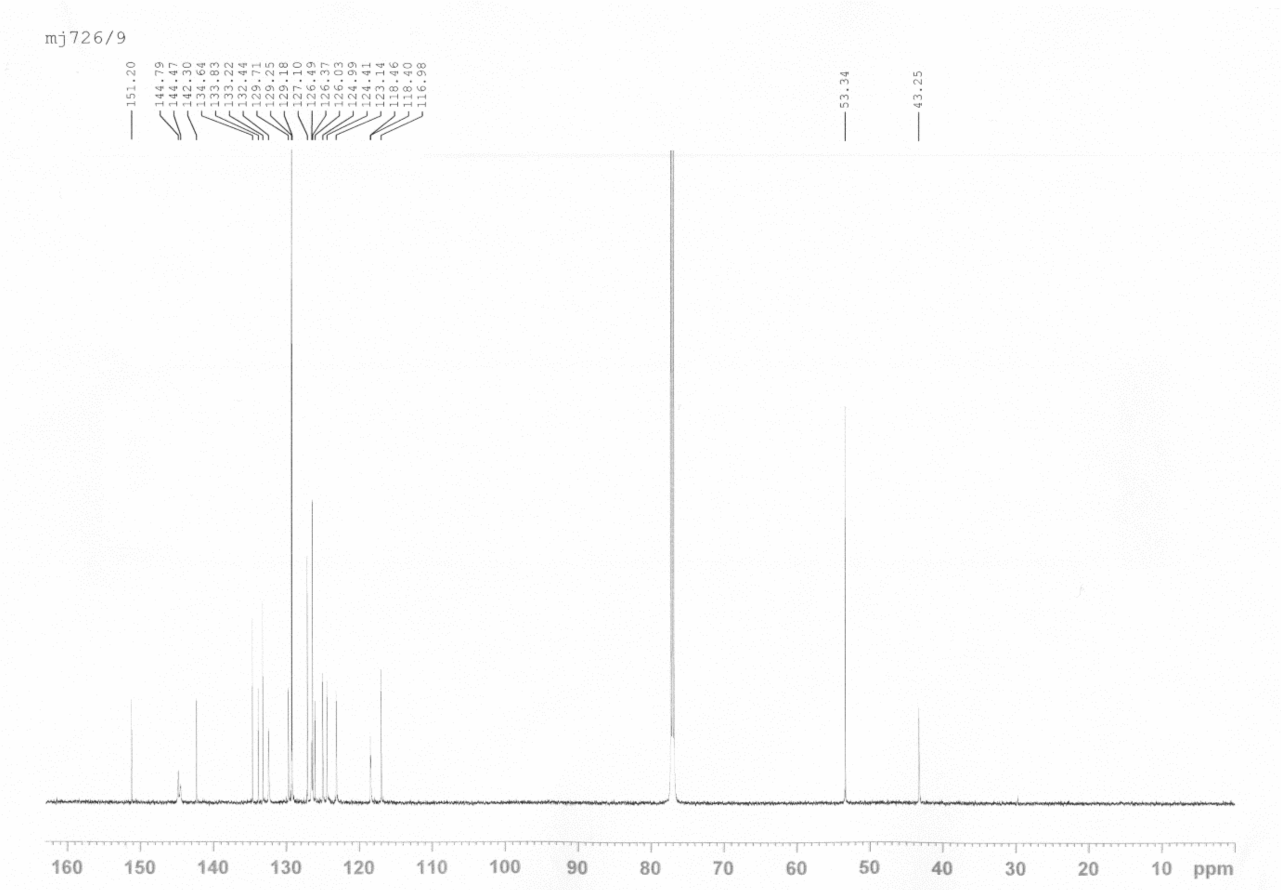

Acquisition Parameter

Source Type	ESI	Ion Polarity	Positive	Set Nebulizer	0.3 Bar
Focus	Active	Set Capillary	4000 V	Set Dry Heater	240 °C
Scan Begin	100 m/z	Set End Plate Offset	-500 V	Set Dry Gas	4.0 l/min
Scan End	1000 m/z	Set Charging Voltage	2000 V	Set Divert Valve	Source
		Set Corona	0 nA	Set APCI Heater	0 °C

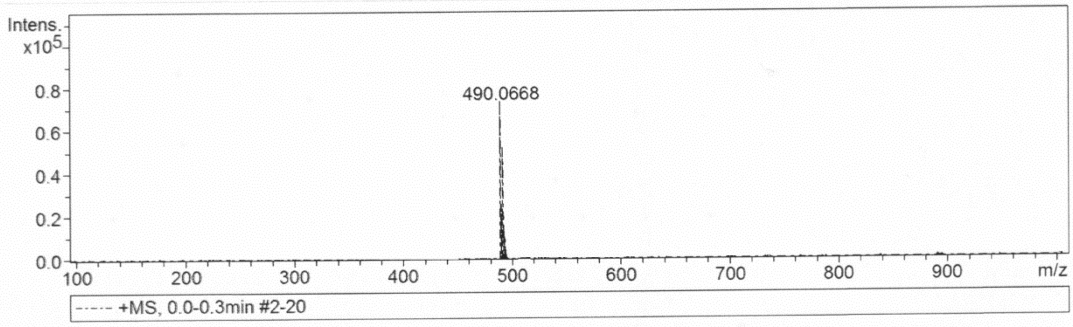

#	m/z	Res.	S/N	I	I %	FWHM
1	490.0668	22910	1750.7	71717	100.0	0.0214
2	492.0641	20719	1225.1	50371	70.2	0.0237

27. NMR spectra and HR MS of 4-((4-((8-chloroquino[3,2-b]benzo[1,4]thiazin-6-yl)methyl)-1*H*-1,2,3-triazol-1-yl)methyl)benzonitrile (**32**).

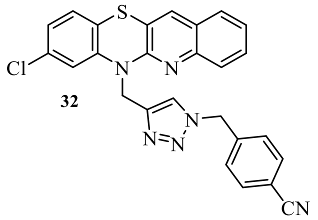

32

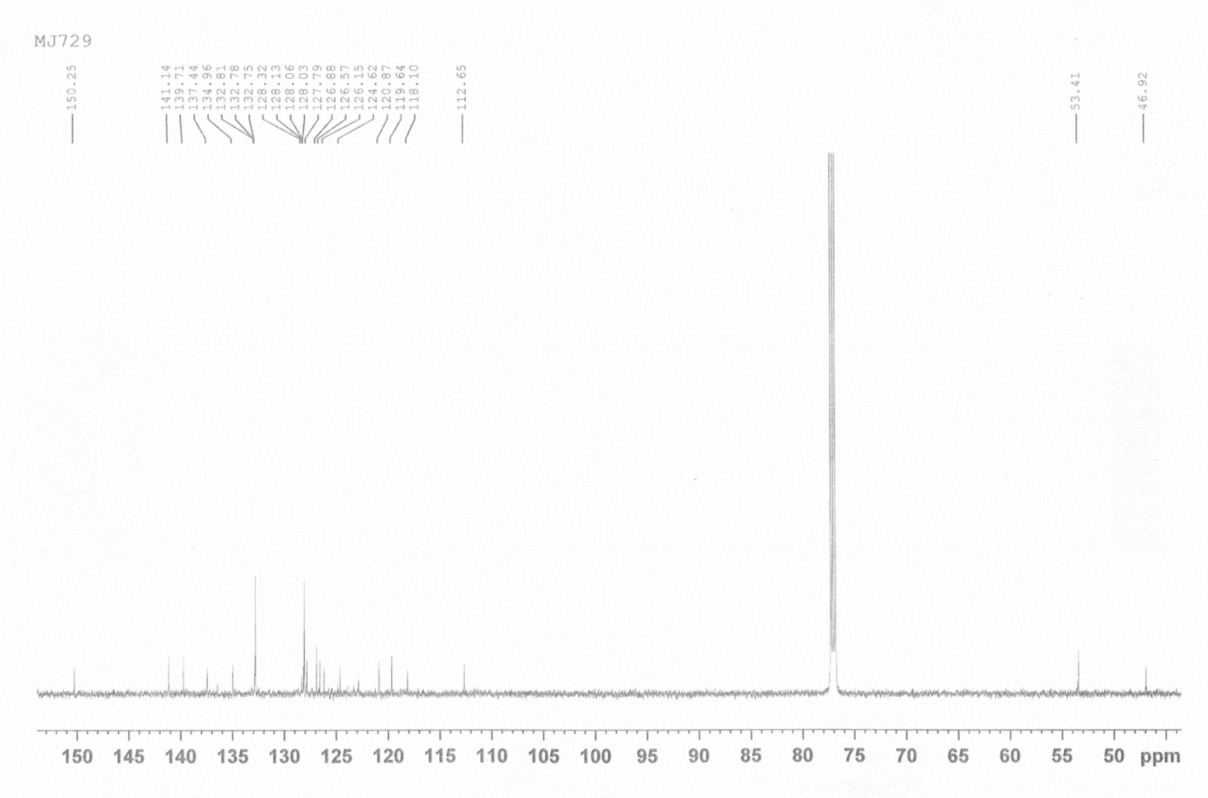

Acquisition Parameter

Source Type	ESI	Ion Polarity	Positive	Set Nebulizer	0.3 Bar
Focus	Active	Set Capillary	4000 V	Set Dry Heater	240 °C
Scan Begin	100 m/z	Set End Plate Offset	-500 V	Set Dry Gas	4.0 l/min
Scan End	1000 m/z	Set Charging Voltage	2000 V	Set Divert Valve	Source
		Set Corona	0 nA	Set APCI Heater	0 °C

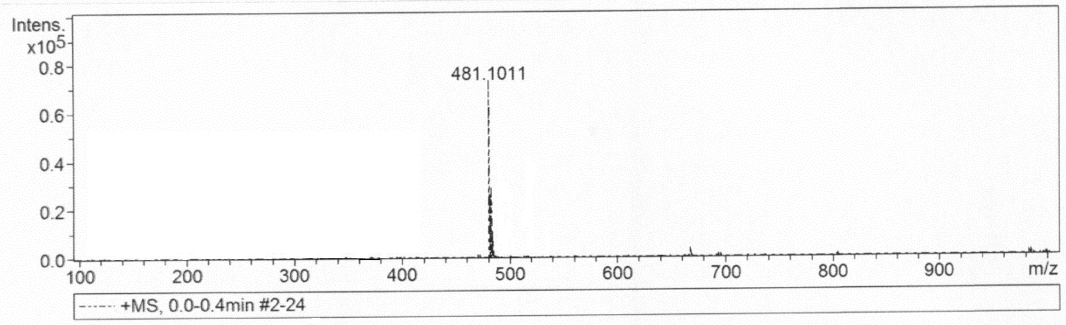

#	m/z	Res.	S/N	I	I %	FWHM
1	481.1011	22002	2483.2	71369	100.0	0.0219

28. NMR spectra and HR MS of 8-chloro-6-((1-((phenylthio)methyl)-1*H*-1,2,3-triazol-4-yl)methyl)-quino[3,2-b]benzo[1,4]thiazine (**33**).

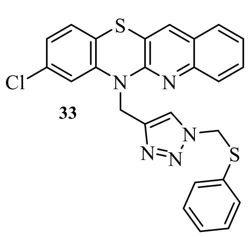

33

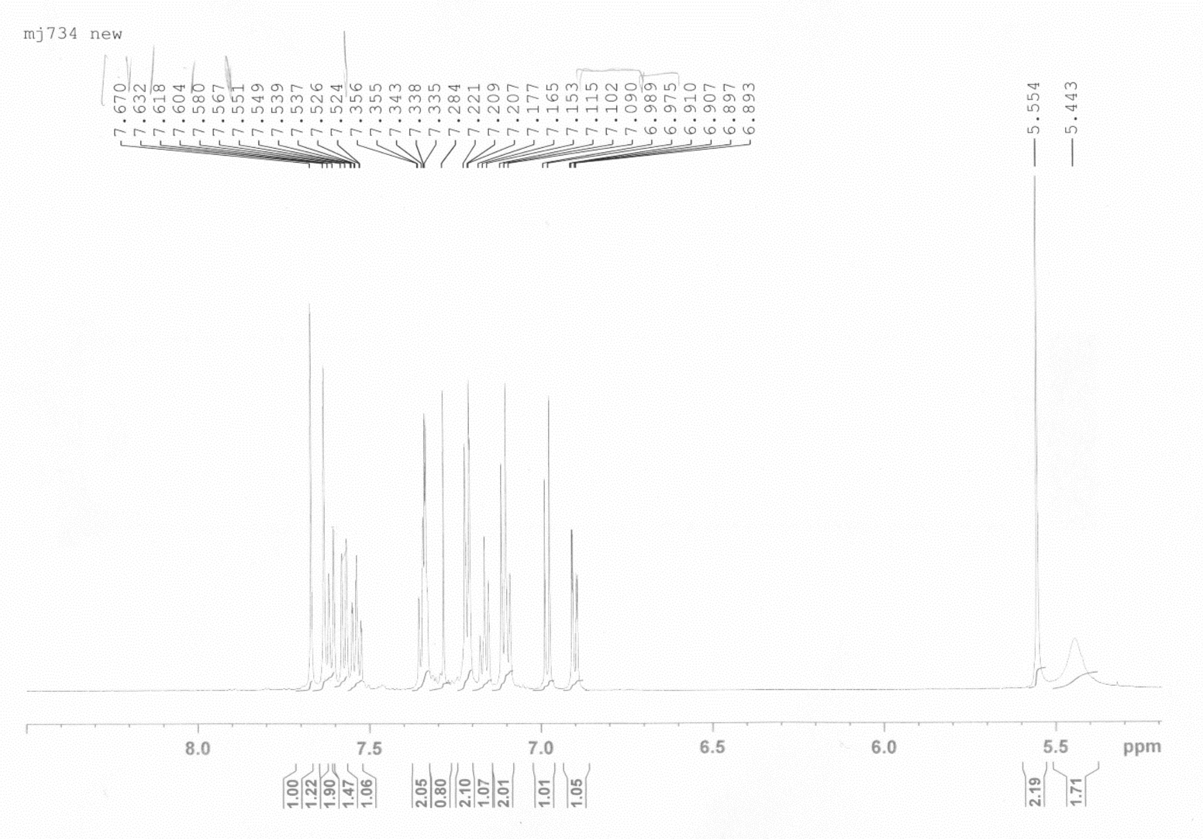

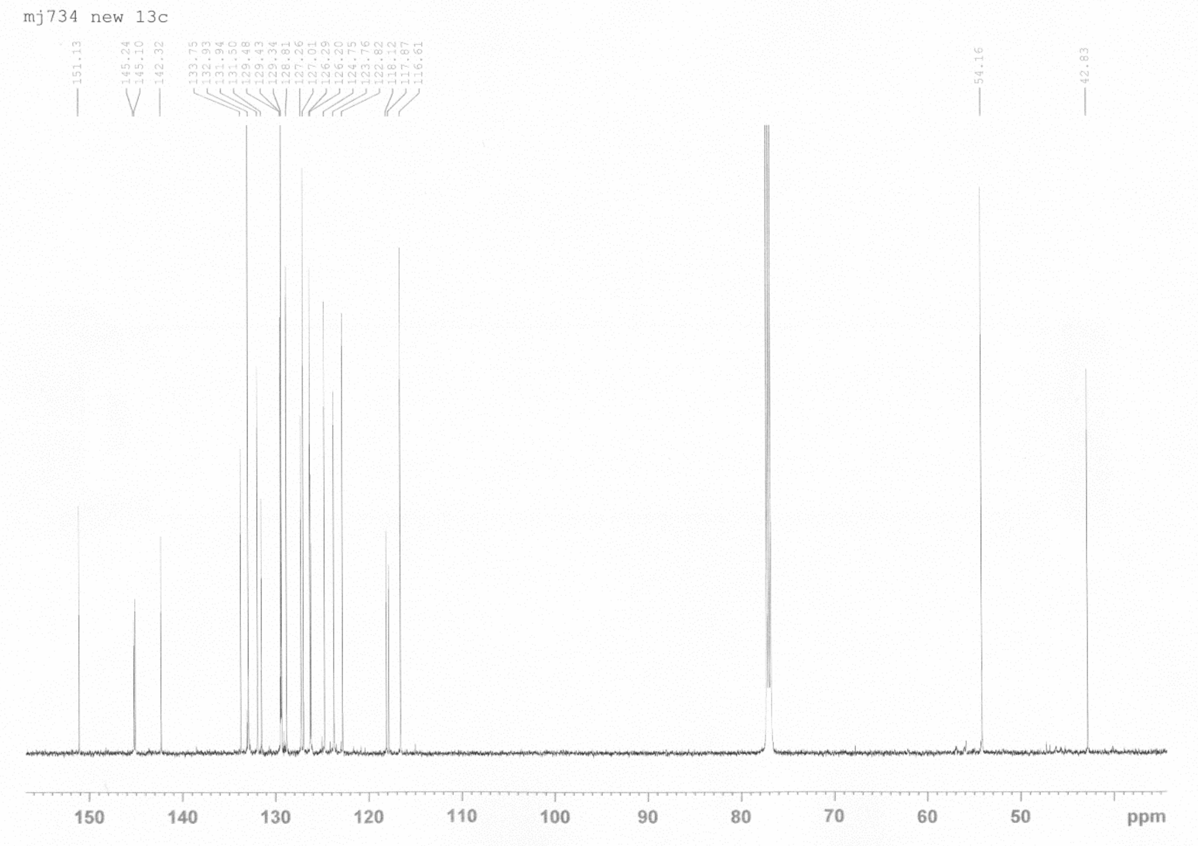

Acquisition Parameter

Source Type	ESI	Ion Polarity	Positive	Set Nebulizer	0.3 Bar
Focus	Active	Set Capillary	4000 V	Set Dry Heater	240 °C
Scan Begin	100 m/z	Set End Plate Offset	-500 V	Set Dry Gas	4.0 l/min
Scan End	1000 m/z	Set Charging Voltage	2000 V	Set Divert Valve	Source
		Set Corona	0 nA	Set APCI Heater	0 °C

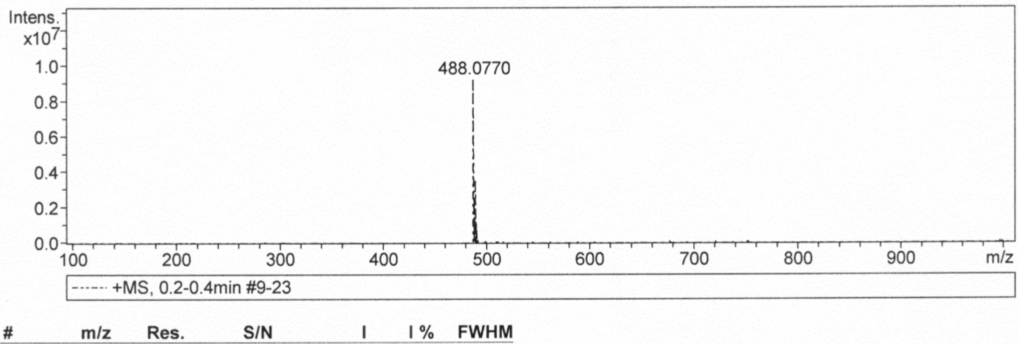

#	m/z	Res.	S/N	I	I %	FWHM
1	488.0770	48345	16381.6	9268264	100.0	0.0101
2	490.0740	43308	6352.0	3594898	38.8	0.0113
